# Practical synthesis of isocoumarins via Rh(III)-catalyzed C–H activation/annulation cascade

**DOI:** 10.3762/bjoc.19.10

**Published:** 2023-01-30

**Authors:** Qian-Ci Gao, Yi-Fei Li, Jun Xuan, Xiao-Qiang Hu

**Affiliations:** 1 Key Laboratory of Catalysis and Energy Materials Chemistry of Ministry of Education & Hubei Key Laboratory of Catalysis and Materials Science, School of Chemistry and Materials Science, South-Central Minzu University, Wuhan 430074, People’s Republic of China; 2 Anhui Province Key Laboratory of Chemistry for Inorganic/Organic Hybrid Functionalized Materials, College of Chemistry & Chemical Engineering, Anhui University, Hefei, Anhui 230601, People’s Republic of China

**Keywords:** C–H activation, enaminone, iodonium ylide, isocoumarin, rhodium catalysis

## Abstract

Herein, we report an unprecedented Rh(III)-catalyzed C–H activation/annulation cascade of readily available enaminones with iodonium ylides towards the convenient synthesis of isocoumarins. This coupling system proceeds in useful chemical yields (up to 93%) via a cascade C–H activation, Rh-carbenoid migratory insertion and acid-promoted intramolecular annulation. The success of gram-scale reaction and diverse functionalization of isocoumarins demonstrated the synthetic utility of this protocol.

## Introduction

**Scheme 1:**
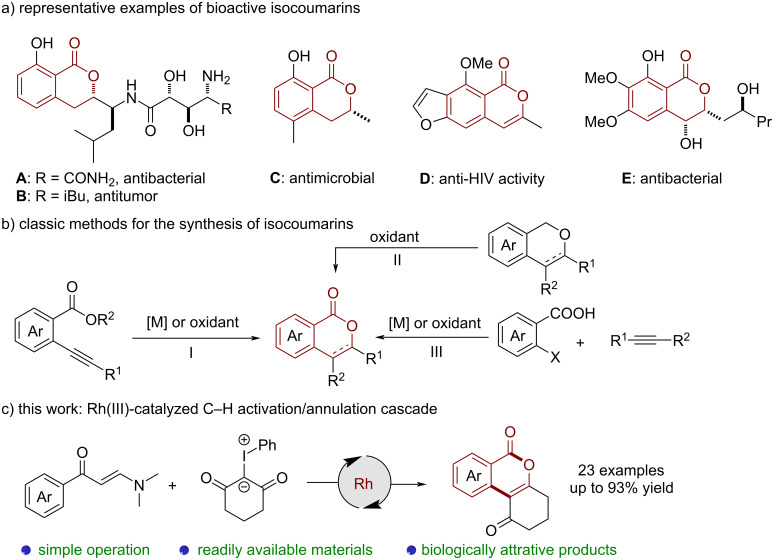
Significance of isocoumarins (a), classic methods for the synthesis of isocoumarins (b) and reaction design (c).

Isocoumarins are an important structural motif in many naturally occurring lactones isolated from bacterial strains, molds, and plants, exhibiting a wide range of pharmacological properties such as antibacterial, antitumor, and anti-HIV activities (Scheme 1a) [1–5]. Fascinated by their versatile properties, researchers were prompted to develop efficient methods for the synthesis of isocoumarin scaffolds. Traditional synthetic strategies including 1) intramolecular cyclization of 2-alkenyl benzoic acids or *o*‑alkynylbenzoates (Scheme 1b, I) [6–10], 2) oxidation of isochromans (Scheme 1b, II) [11–12], or 3) metal-catalyzed cross-coupling/cyclization of 1,2-difunctionalized arenes with alkynes or carbon monoxide (Scheme 1b, III) [13–16], have been widely applied for the assembly of isocoumarins over the past decades. Recently, the transition-metal-catalyzed ortho C–H activation/annulation of benzoic acids has emerged as an attractive approach towards isocoumarins [17–18]. Pioneering examples relying on the Pd, Ru, and Ir-catalyzed C–H cross coupling of benzoic acids with alkenes and alkynes were realized by the groups of Miura [19], Lee [20], Ackermann [21], Zhang [22], Jiang [23], and Jeganmohan [24] et al. Despite these impressive achievements, established methods often require the use of stoichiometric oxidants or harsh conditions, thus limiting their broad applicability. Consequently, it is still highly desirable to exploit innovative and convenient methods for the rapid construction of isocoumarins.

Enaminones are bench stable and easily available, which have been established as versatile synthetic building blocks for the synthesis of cyclic scaffolds [25]. In 2016, Zhu et al. reported the first example of a Rh-catalyzed C–H functionalization of enaminones with alkynes and α-diazo-β-ketoesters to access naphthalenes [26]. Very recently, the same group developed an efficient Rh(III)-catalyzed C–H cross-coupling of enaminones with diazodicarbonyls for the divergent construction of isocoumarins and naphthalenes [27]. Moreover, Loh et al. disclosed a Rh-catalyzed formal [4 + 2] cycloaddition of enaminones with diazocarbonyls [28]. Compared with highly sensitive diazo compounds, iodonium ylides are known to show ready availability and good stability [29–30]. Our group has recently demonstrated that iodonium ylides can be used as carbene precursors in the Rh-catalyzed [4 + 2] cyclization of pyrazolidinones [31]. During the preparation of manuscript, the group of Li reported a similar Rh(III)-catalyzed [3 + 3] annulation of enaminones with iodonium ylides [32]. Inspired by the collected contributions [26–28] and based on our ongoing research in C–H activation [33–35], we recently wondered whether it might be possible to couple iodonium ylides with enaminones in a Rh(III)-catalyzed C–H activation/annulation cascade reaction for the rapid construction of isocoumarins (Scheme 1c).

## Results and Discussion

Our initial experiment was performed with enaminone **1a** and iodonium ylide **1b** in the presence of [Cp*RhCl_2_]_2_ (5 mol %) as the catalyst, AgSbF_6_ (10 mol %) and KOAc (50 mol %) as additives in 1,2-dichloroethane (DCE) at 100 °C for 16 hours. To our delight, the desired isocoumarin **3aa** was obtained in 42% yield (Table 1, entry 1). Then, the influence of solvents has been subsequently investigated. As a result, DCE proved to be the optimal solvent, while other commonly used solvents such as toluene, dioxane, and ethanol gave inferior yields (Table 1, entries 2–4, 14–39%). Further screening of bases did not improve the outcome of the product, whereas 63% yield of **3aa** was obtained when acetic acid was added into the reaction system (Table 1, entry 8). Increasing the amount of acetic acid significantly improved the reaction efficiency delivering product **3aa** in 80% yield (Table 1, entry 9). The choice of a suitable catalyst was the key factor for the success of this reaction since only a trace amount of **3aa** can be obtained by using [{Ru(*p*-cymene)Cl_2_}]_2_ as a catalyst (Table 1, entry 10).

**Table 1:** Optimization of the reaction conditions^a^.

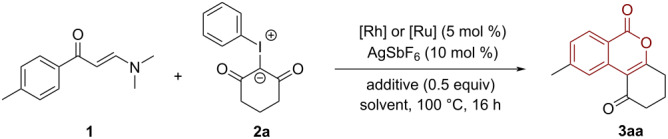				

entry	catalyst	additive	solvent	yield^b^

1	[Cp*RhCl_2_]_2_	KOAc	DCE	42
2	[Cp*RhCl_2_]_2_	KOAc	toluene	39
3	[Cp*RhCl_2_]_2_	KOAc	dioxane	16
4	[Cp*RhCl_2_]_2_	KOAc	EtOH	14
5	[Cp*RhCl_2_]_2_	Cs(OPiv)_2_	DCE	47
6	[Cp*RhCl_2_]_2_	Cs(OAc)_2_	DCE	51
7	[Cp*RhCl_2_]_2_	K_2_CO_3_	DCE	27
8	[Cp*RhCl_2_]_2_	AcOH	DCE	63
9^c^	[Cp*RhCl_2_]_2_	AcOH	DCE	80
10	[{Ru(*p*-cymene)Cl_2_}]_2_	AcOH	DCE	trace

^a^Standard conditions: **1a** (0.2 mmol), **2a** (0.6 mmol), catalyst (5 mol %), AgSbF_6_ (10 mol %), additive (0.5 equiv), solvent (2.0 mL) at 100 °C for 16 h. ^b^Isolated yields. ^c^AcOH (5.0 equiv) was used.

**Scheme 2:**
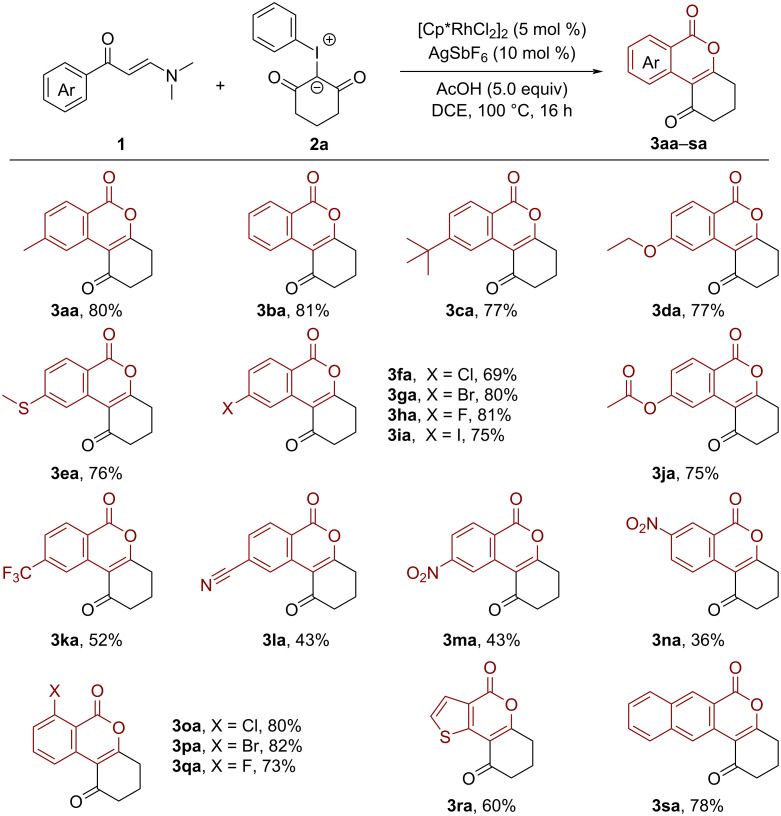
Scope of enaminones.

With the optimal conditions in hand, we then investigated the scope of this Rh-catalyzed C–H activation/annulation cascade reaction. As shown in Scheme 2, a range of functionalized enaminones were compatible with this Rh-catalytic system, furnishing the corresponding isocoumarin products in satisfying yields. For example, enaminones bearing electron-donating (Me, OEt and *t*-Bu), as well as electron-withdrawing groups (F, Cl, Br, I, CF_3_, CN and NO_2_) at *ortho*, *meta* or *para*-positions were well tolerated in this transformation, delivering a variety of structurally diverse isocoumarins in an efficient manner (**3aa**–**qa**, 43–82%). It is worth mentioning that the tolerance of halogen substituents (Cl, Br and I) may open up a new opportunity for further transition-metal-catalyzed cross-coupling reactions. Sensitive groups, such as ester, trifluoromethyl and nitro substituents, were retained unchanged in the final products (**3ja**, **3ka**, **3ma,** and **3na**). Also the strongly coordinating thioether substituent proved to be suitable for this protocol, providing the desired product **3ea** in 76% yield. Moreover, under the standard conditions, 3-thienyl and 2-naphthyl-substituted enaminones were smoothly coupled with iodonium ylide **1b** to give the expected isocoumarins **3ra** and **3sa** in 60% and 78%, respectively.

**Scheme 3:**
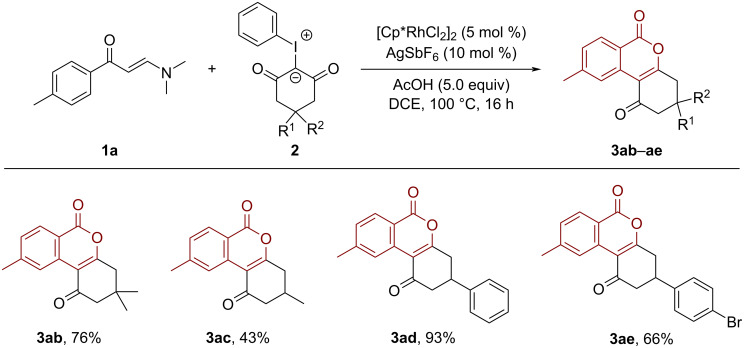
Scope of iodonium ylides.

Next, we sought to test the generality of this reaction with respect to iodonium ylides. As outlined in Scheme 3, iodonium ylides featuring dimethyl, methyl, and phenyl groups underwent the current reaction efficiently, delivering the desired products **3ab**–**ae** in moderate to good yields (43–93%).

**Scheme 4:**
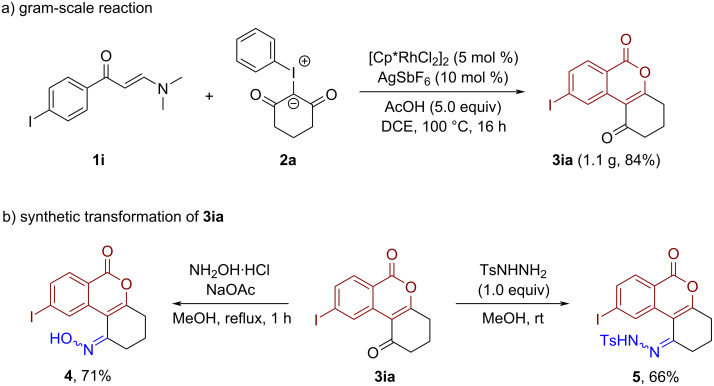
Gram-scale reaction (a) and synthetic transformation (b).

To demonstrate the synthetic utility of this methodology, a gram-scale synthesis of isocoumarin **3ia** was firstly performed. Under the optimal conditions, the desired product **3ia** was successfully obtained in 84% yield (1.1 g) via a simple recrystallization from the reaction mixture (Scheme 4a). In the presence of hydroxylamine hydrochloride, the carbonyl group of the ketone can be selectively converted into an oxime product **4** (Scheme 4b, 71% yield). In addition, the reaction of the isocoumarin **3ia** with *p*-toluenesulfonyl hydrazide proceeded smoothly to deliver hydrazone **5** in 66% yield (Scheme 4b, right). Of note, oxime and hydrazone compounds are versatile synthetic building blocks, which have been widely applied in transition-metal-catalyzed cross-coupling reactions and radical transformations [36–38].

**Scheme 5:**
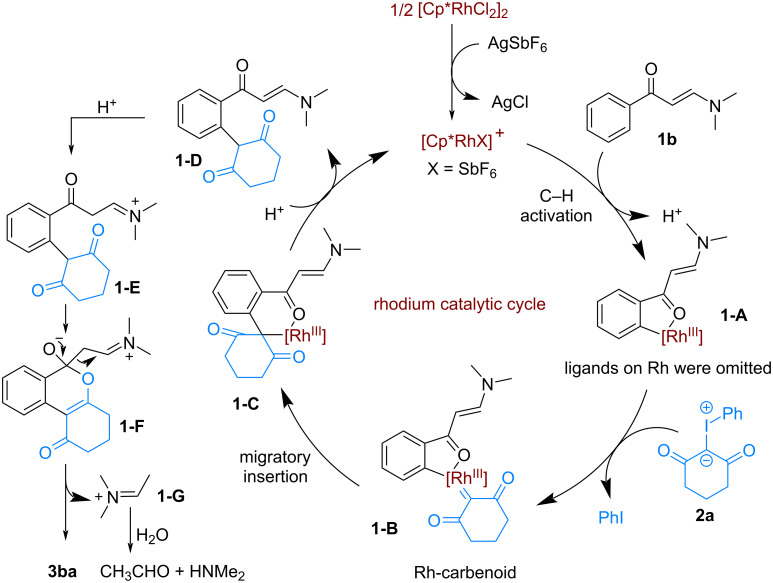
Proposed mechanism.

Based on the literature precedents [27] and our previous work [33–35], a mechanism for this Rh-catalyzed C–H activation/annulation reaction was proposed and depicted in Scheme 5. In the presence of AgSbF_6_, dimeric [Cp*RhCl_2_]_2_ transforms into the active Rh catalyst. Subsequently, the oxygen atom of the enaminone is coordinated to the Rh catalyst, following by a Rh(III)-promoted ortho C–H activation to form a five-membered ruthenacycle **1-A**. Then, the reaction of the iodonium ylide with intermediate **1-A** generates a Rh-carbenoid intermediate **1-B**, which undergoes a rapid migratory insertion to give intermediate **1-C**. The protonation of **1-C** produces the intermediate **1-D** with the regeneration of the active Rh catalyst for the next catalytic cycle. Under acidic conditions, the further protonation of compound **1-D** delivers an imine intermediate **1-E**, which undergoes an intramolecular annulation to give **1-F**. The final isocoumarin product **3ba** can be generated from **1-F** by elimination of imine **1-G** [39]. Finally, the rapid hydrolysis of the resulting **1-G** gives rise to acetaldehyde and dimethylamine as byproducts.

## Conclusion

In summary, an efficient Rh-catalyzed C–H activation/annulation reaction of enaminones with iodonium ylides has been developed. This reaction features simple operation and readily available substrates, enabling the rapid construction of biologically attractive isocoumarins in useful to good yields. The success of a gram-scale reaction and diverse functionalization of the isocoumarin products highlight the tremendous synthetic potential of this methodology in chemical synthesis and drug discovery.

## Experimental

A 10 mL screw-cap vial was charged with enaminone **1** (0.2 mmol), iodonium ylide **2** (0.6 mmol), [Cp*RhCl_2_]_2_ (6.2 mg, 5 mol %), AgSbF_6_ (6.9 mg, 10 mol %), HOAc (60.0 mg, 1.0 mmol) and DCE (2 mL) under N_2_ atmosphere. Then, the reaction mixture was stirred at 100 °C for 16 h. The crude product was purified by flash chromatography on silica gel (petroleum ether/ethyl acetate 5:1) directly to give the desired products **3**. (Note: a heating module was used for the preparation of isocoumarin products **3**.)

## Supporting Information

File 1 Experimental and copies of spectra.
