# Supplementary material for: Practical synthesis of isocoumarins via Rh(III)-catalyzed C–H activation/annulation cascade
Source: Beilstein J Org Chem. 2023 Jan 30;19:100–6. doi: 10.3762/bjoc.19.10 (PMC9907013; doi:10.3762/bjoc.19.10)

BEILSTEIN JOURNAL OF ORGANIC CHEMISTRY

Supporting Information

for

Practical synthesis of isocoumarins via Rh(III)-catalyzed C–H activation/annulation cascade

Qian-Ci Gao, Yi-Fei Li, Jun Xuan and Xiao-Qiang Hu

Beilstein J. Org. Chem. **2023,** *19,* 100–106. doi:10.3762/bjoc.19.10

Experimental and copies of spectra

License and Terms: This is a supporting information file under the terms of the Creative Commons Attribution License (https://creativecommons.org/licenses/by/4.0). Please note that the reuse, redistribution and reproduction in particular requires that the author(s) and source are credited and that individual graphics may be subject to special legal provisions.
The license is subject to the *Beilstein Journal of Organic Chemistry* terms and conditions: (https://www.beilstein-journals.org/bjoc/terms)

Table of contents

1. General information.....S2

2. Preparation of substrates.....S3

2.1 General procedure for preparation of enaminones **1a–s**.....S3

2.2 General procedure for preparation of iodonium ylides **2b–e**.....S3

3. Gram-scale reaction, synthesis of compounds **4** and **5** and spectral data of the products.....S4

3.1 Gram-scale reaction.....S4

3.2 Synthesis of compounds **4** and **5**.....S4

3.3 Spectral data of the products **3aa–sa**, **3ab–ae**, **4** and **5**.....S5

4. NMR Spectra of products **3aa–sa**, **3ab–ae**, **4** and **5**.....S14

1. General information

Unless otherwise noted, materials were purchased from commercial suppliers (Alfa, TCI and Sigma-Aldrich etc.), and used without further purification. All the solvents were treated according to general methods. All reactions were monitored by thin-layer chromatography (TLC) on silica gel plates using UV light as visualizing agent (if applicable). Flash column chromatography was performed using 200–300 mesh silica gel. ^{1}H NMR spectra were recorded on 400 and 600 MHz spectrometers. Chemical shifts are reported in delta (δ (ppm)) units in parts per million (ppm) relative to the singlet (0 ppm) for tetramethylsilane (TMS). Data are reported as follows: chemical shift, multiplicity (s = singlet, d = doublet, t = triplet, q = quartet, dd = doublet of doublets, m = multiplet), coupling constants (Hz) and integration. ^{13}C NMR spectra were recorded on a Varian Mercury 100 MHz spectrometer with complete proton decoupling ($CDCl_3$: 77.0 ppm, DMSO: 39.6 ppm). The high resolution mass spectra (HRMS) were measured on a Shimadzu LCMS-IT-TOF mass spectrometer or a DIONEX UltiMate 3000 & Bruker Compact TOF mass spectrometer by ESI. Measured values are reported to 4 decimal places of the calculated value. The calculated values are based on the most abundant isotope. An oil bath was used for the synthesis of phenethylamine and a heating module was used for preparation of compounds **3aa–sa**, **3ab–ae**, **4**, and **5**.

2. Preparation of substrates

2.1 General procedure for preparation of enaminones 1a–s

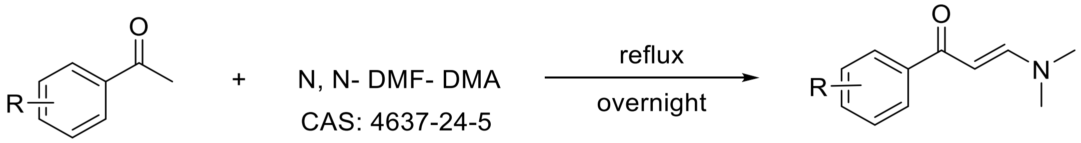

To a round-bottomed flask were added acetophenone derivatives (5.0 mmol) and *N,N*-DMF-DMA (2.0 equiv). The reaction mixture was heated to reflux overnight. Then, the volatiles were removed under reduced pressure. The residue was purified by flash column chromatography (PE/EA 2:1) to give the enaminone substrates **1a**–**s**. [1-3]

2.2 General procedure for preparation of iodonium ylides 2b–e

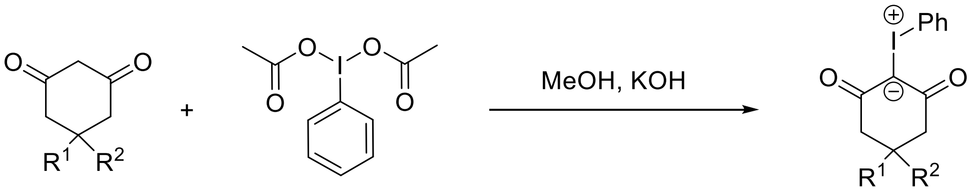

To a solution of the 1,3-dione (14 mmol) in 30 mL methanol, were added 20 mL of a 10% aq solution of KOH, followed by the addition of a solution of diacetoxy iodobenzene (15 mmol) in 40 mL methanol. The reaction mixture was stirred for 2 h at room temperature and then quenched with ice cold water. The resulting white precipitate was filtered, the mother liquor was extracted with dichloromethane, then washed with water, dried over anhydrous sodium sulfate, filtered, and concentrated in vacuo. The resultant white solid was mixed with the first crop and recrystallized from DCM/hexane. [4-6]

3. Gram-scale reaction, synthesis of compounds 4 and 5 and spectral data of the products

3.1 Gram-scale reaction

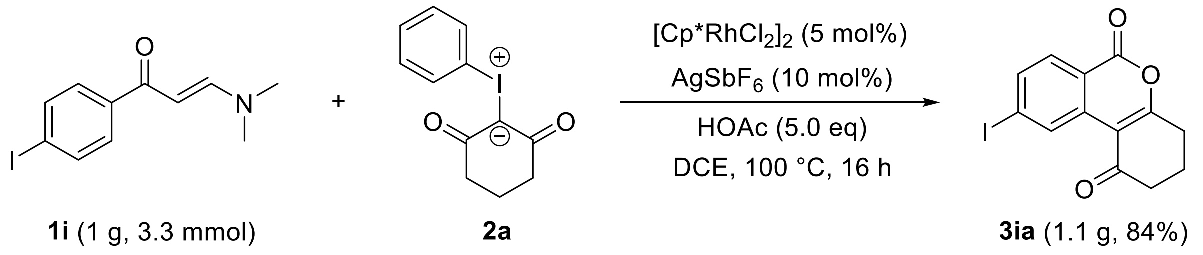

Compounds **1i** (1 g, 3.3 mmol), **2a** (3.1 g, 9.9 mmol), $[Cp^*RhCl_2]_2$ (102.0 mg, 5 mol %), $AgSbF_6$ (110 mg, 10 mol %), and HOAc (996.8 mg, 1.0 mmol) were dissolved in DCE (33 mL) under N_2 atmosphere. Then, the reaction mixture was stirred at 100 °C for 16 h. The desired product **3ia** was successfully obtained in 84% yield (1.1 g) via a simple recrystallization from the reaction mixture.

3.2 Synthesis of compounds 4 and 5

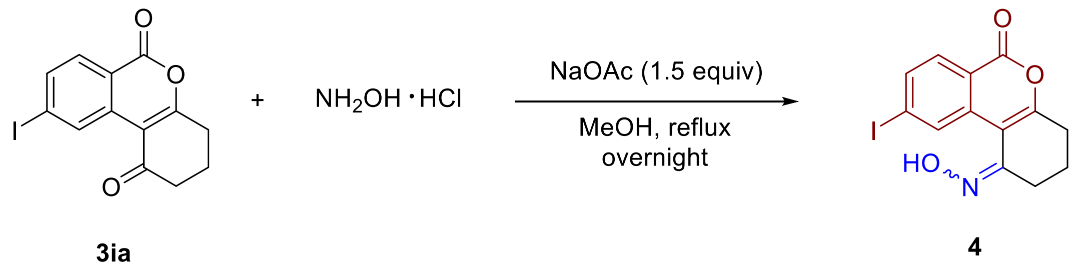

Compound **3ia** (68.0 mg, 0.2 mmol), NaOAc (41.0 mg, 0.5 mmol), and hydroxylamine hydrochloride (20.8 mg, 0.3 mmol) were dissolved in MeOH/H_2O (0.6 mL/1.0 mL). Then, the reaction mixture was heated to reflux overnight. The crude product was purified by flash chromatography on silica gel to afford pure product **4** as a white solid in 71% yield.

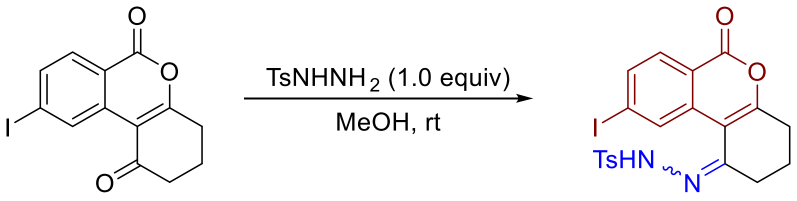

3ia **5**

Compound **3ia** (68.0 mg, 0.2 mmol) and tosylhydrazide (186.2 mg, 1.0 mmol) were dissolved in MeOH (2 mL). Then, the reaction mixture was stirred at room temperature overnight. The crude product was purified by flash chromatography on silica gel to afford compound **5** as a white solid in 66% yield.

3.3 Spectral data of the products 3aa–sa, 3ab–ae, 4 and 5

Product 3aa (known compound, CAS: 2072821-65-7)

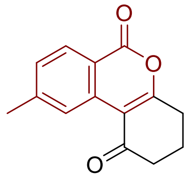

The crude product was purified by column chromatography (SiO_2, petroleum ether/ethyl acetate 10:1 to 5:1), yielding **3aa** as a white solid (36.52 mg, 80% yield).

The analytical data of compound **3aa** is in agreement with the literature report.[7] ^{1}H NMR (400 MHz, chloroform-d) δ = 8.77 (s, 1H), 8.07 (d, J = 8.1 Hz, 1H), 7.27 (d, J = 7.8 Hz, 1H), 2.85 (t, J = 6.3 Hz, 2H), 2.58 (t, J = 7.1 Hz, 2H), 2.43 (s, 3H), 2.06 – 2.12 (m, 2H). ^{13}C NMR (101 MHz, chloroform-d) δ = 197.0, 169.5, 160.4, 146.9, 133.9, 129.6, 129.4, 126.0, 117.2, 111.4, 38.9, 28.9, 22.4, 19.9.

Product 3ba (known compound, CAS: 5651-59-2)

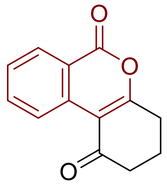

The crude product was purified by column chromatography (SiO_2, petroleum ether/ethyl acetate 10:1 to 5:1), yielding **3ba** as a white solid (34.9 mg, 81% yield).

The analytical data of compound **3ba** is in agreement with the literature report.[7] ^{1}H NMR (400 MHz, chloroform-d) δ = 9.04 (d, J = 8.3 Hz, 1H), 8.27 (d, J = 7.9 Hz, 1H), 7.79 (t, J = 8.0 Hz, 1H), 7.53 (t, J = 7.6 Hz, 1H), 2.94 (t, J = 6.3 Hz, 2H), 2.66 (t, J = 7.0 Hz, 2H), 2.15 - 2.21 (m, 2H). ^{13}C NMR (101 MHz, chloroform-d) δ = 196.8, 169.4, 160.4, 135.5, 134.0, 129.5, 128.3, 126.0, 119.8, 111.5, 38.9, 28.9, 19.9.

Product 3ca (known compound, CAS: 2676136-71-1)

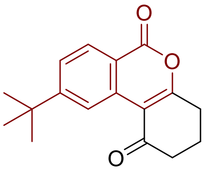

The crude product was purified by column chromatography (SiO_2, petroleum ether/ethyl acetate 10:1 to 5:1), yielding **3ca** as a white solid (41.5 mg, 77% yield). The analytical data of compound **3ca** is in agreement with the literature report.[7] ^{1}H NMR (400 MHz, chloroform-d) δ = 9.15 (s, 1H), 8.20 (d, J = 8.2 Hz, 1H), 7.58 (d, J = 8.8 Hz, 1H), 2.94 (t, J = 6.3 Hz, 2H), 2.67 (t, J = 7.0 Hz, 2H), 2.14 - 2.21 (m, 2H), 1.40 (s, 9H). ^{13}C NMR (101 MHz, chloroform-d) δ = 197.1, 169.5, 160.4, 159.8, 134.0, 129.4, 126.0, 122.7, 117.3, 111.8, 39.1, 35.8, 31.0, 29.0, 20.0.

Product 3da (known compound, CAS: 2759083-08-2)

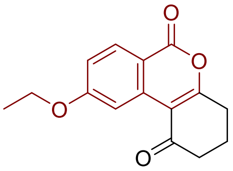

The crude product was purified by column chromatography (SiO_2, petroleum ether/ethyl acetate 10:1 to 5:1), yielding **3da** as a white solid (40.0 mg, 77% yield). The analytical data of compound **3da** is in agreement with the literature report.[8] ^{1}H NMR (400 MHz, chloroform-d) δ = 8.58 (d, J = 2.5 Hz, 1H), 8.18 (d, J = 8.9 Hz, 1H), 7.02 - 7.05 (dd, J = 8.8, 2.5 Hz, 1H), 4.17- 4.22 (m, 2H), 2.93 (t, J = 6.3 Hz, 2H), 2.65 (t, J = 7.1 Hz, 2H), 2.14 - 2.20 (m, 2H), 1.63 (s, 1H), 1.48 (t, J = 7.0 Hz, 3H). ^{13}C NMR (101 MHz, chloroform-d) δ = 197.2, 170.3, 164.8, 160.2, 136.4, 131.6, 117.2, 112.5, 111.3, 108.8, 64.1, 39.0, 29.1, 20.0, 14.5.

Product 3ea

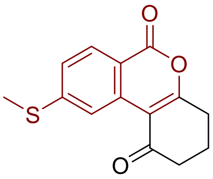

The crude product was purified by column chromatography (SiO_2, petroleum ether/ethyl acetate 10:1 to 5:1), yielding **3ea** as a white solid (39.7 mg, 76% yield). ^{1}H NMR (400 MHz, chloroform-d) δ = 8.90 (s, 1H), 8.11 (d, J = 8.4 Hz, 1H), 7.33 (t, J = 8.0 Hz, 1H), 2.93 (t, J = 6.3 Hz, 2H), 2.65 (t, J = 7.0 Hz, 2H), 2.59 (s, 3H), 2.14 - 2.21 (m, 2H). ^{13}C NMR (101 MHz, chloroform-d) δ = 197.0, 170.4, 160.2, 150.0, 134.2, 129.4, 125.3, 120.5, 115.7, 111.0, 39.0, 29.1, 19.9, 14.6. M.P.: 150.0 - 152.0 °C HRMS (ESI): m/z $[M+Na]^+$ calcd for $C_{14}H_{12}O_3S$: 283.0399; found: 283.0393.

Product 3fa (known compound, CAS: 2249868-02-6)

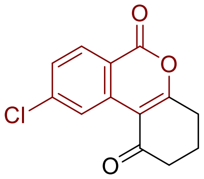

The crude product was purified by column chromatography (SiO_2, petroleum ether/ethyl acetate 10:1 to 5:1), yielding **3fa** as a white solid (34.7 mg, 69% yield). The analytical data of compound **3fa** is in agreement with the literature report.[7] ^{1}H NMR (400 MHz,

chloroform-d) δ = 9.11 (d, J = 2.0 Hz, 1H), 8.19 (d, J = 8.5 Hz, 1H), 7.48 (dd, J = 8.8, 2.5 Hz, 1H), 2.95 (t, J = 6.3 Hz, 2H), 2.67 (t, J = 7.2 Hz, 2H), 2.15 - 2.21 (m, 2H). ^{13}C NMR (101 MHz, chloroform-d) δ = 196.4, 170.6, 159.6, 142.7, 135.2, 131.0, 128.9, 126.0, 118.2, 110.7, 38.7, 29.0, 19.8.

Product 3ga (known compound, CAS: 2072821-66-8)

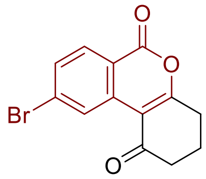

The crude product was purified by column chromatography (SiO_2, petroleum ether/ethyl acetate 10:1 to 5:1), yielding **3ga** as a white solid (46.7 mg, 80% yield). The analytical data of compound **3ga** is in agreement with the literature report.[7] ^{1}H NMR (400 MHz, chloroform-d) δ = 9.29 (s, 1H), 8.12 (d, J = 8.4 Hz, 1H), 7.66 (d, J = 8.1 Hz, 1H), 2.95 (t, J = 6.3 Hz, 2H), 2.66 (t, J = 7.0 Hz, 2H), 2.15 - 2.21 (m, 2H). ^{13}C NMR (101 MHz, chloroform-d) δ = 196.4, 170.5, 159.8, 135.3, 131.9, 131.7, 131.0, 129.0, 118.6, 110.6, 38.8, 29.0, 19.9.

Product 3ha (known compound, CAS: 2676136-73-3) [7]

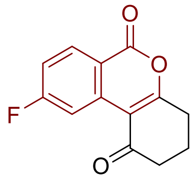

The crude product was purified by column chromatography (SiO_2, petroleum ether/ethyl acetate 10:1 to 5:1), yielding **3ha** as a white solid (37.6 mg, 81% yield).
The analytical data of compound **3ha** is in agreement with the literature report.[7] ^{1}H NMR (400 MHz, chloroform-d) δ = 8.81 (dd, J = 11.3, 2.6 Hz, 1H), 8.28 - 8.32 (m, 1H), 7.20 - 7.25 (m, 1H), 2.95 (t, J = 6.3 Hz, 2H), 2.67 (t, J = 7.2 Hz, 2H), 2.16 - 2.22 (m, 2H). ^{13}C NMR (101 MHz, chloroform-d) δ = 196.6, 170.6, 167.2 (d, J = 254.4 Hz), 159.5, 136.7 (d, J = 12.3 Hz), 132.7 (d, J = 10.5 Hz), 116.6 (d, J = 23.4 Hz), 116.3 (d, J = 2.2 Hz), 112.8 (d, J = 25.9 Hz), 112.6 (d, J = 2.9 Hz), 38.8, 29.0, 19.9.

Product 3ia

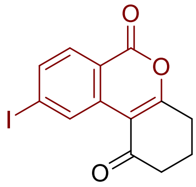

The crude product was purified by column chromatography (SiO_2, petroleum ether/ethyl acetate 10:1 to 5:1), yielding **3ia** as a white solid (51.1 mg, 75% yield). 1H NMR (400 MHz, chloroform-d) δ = 9.50 (s, 1H), 7.94 (d, J = 8.3 Hz, 1H), 7.88 (d, J = 8.3 Hz, 1H), 2.94 (t, J = 6.3 Hz, 2H), 2.66 (t, J = 6.7 Hz, 2H), 2.14 - 2.21 (m, 2H). ^{13}C NMR (101 MHz, chloroform-d) δ = 196.4, 170.3, 160.1, 137.7, 135.1, 134.9, 130.6, 119.0, 110.4, 105.0, 38.8, 29.0, 19.9. M.P.: 195.0 - 197.0 °CHRMS (ESI): m/z [M+Na]$^+$ calcd for $C_{13}H_9IO_3$: 362.9498; found: 362.9469.

Product 3ja

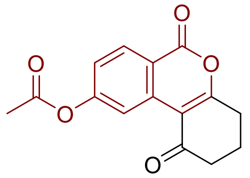

The crude product was purified by column chromatography (SiO_2, petroleum ether/ethyl acetate 10:1 to 5:1), yielding **3ja** as a white solid (41.0 mg, 75% yield). ^{1}H NMR (400 MHz, chloroform-d) δ = 8.60 (d, J = 2.6 Hz, 1H), 8.18 (d, J = 8.8 Hz, 1H), 7.04 (dd, J = 8.5, 2.6 Hz, 1H), 3.95 (s, 3H), 2.93 (t, J = 6.3 Hz, 2H), 2.65 (t, J = 6.5 Hz, 2H), 2.14 - 2.20 (m, 2H). ^{13}C NMR (101 MHz, chloroform-d) δ = 197.2, 170.3, 164.8, 160.2, 136.4, 131.6, 117.2, 112.6, 111.3, 108.8, 64.1, 39.0, 29.1, 20.0, 14.5. M.P.: 200.0 - 202.0 °C HRMS (APCI): m/z $[M+H]^+$ calcd for $C_{15}H_{12}O_5$: 273.0757; found: 273.0768.

Product 3ka (known compound, CAS: 2072821-67-9)

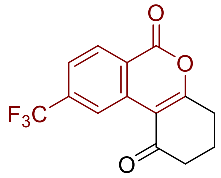

The crude product was purified by column chromatography (SiO_2, petroleum ether/ethyl acetate 10:1 to 5:1), yielding **3ka** as a white solid (29.5 mg, 52% yield). The analytical data of compound **3ka** is in agreement with the literature report.[8] ^{1}H NMR (400 MHz, chloroform-d) δ = 9.43 (d, J = 1.7 Hz, 1H), 8.40 (d, J = 8.3 Hz, 1H), 7.76 (d, J = 8.2 Hz, 1H), 2.98 (t, J = 6.3 Hz, 2H), 2.69 (t, J = 6.5 Hz, 2H)), 2.18 - 2.24 (m, 2H). ^{13}C NMR (101 MHz, chloroform-d) δ = 196.4, 170.5, 159.3, 136.8 (q, J = 32.5 Hz) 134.6, 130.3, 130.0, 124.7 (q, J = 3.3 Hz), 123.6 (q, J = 4.3 Hz), 122.7 (q, J = 165.3 Hz), 111.0, 38.6, 29.0, 19.8.

Product 3la (known compound, CAS: 2759083-11-7)

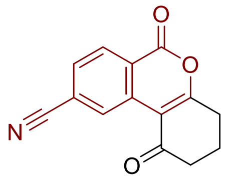

The crude product was purified by column chromatography (SiO_2, petroleum ether/ethyl acetate 10:1 to 5:1), yielding **3la** as a white solid (20.6 mg, 43% yield). The analytical data of compound **3la** is in agreement with the literature report.[8] ^{1}H NMR (400 MHz, chloroform-d) δ = 9.49 (d, J = 1.6 Hz, 1H), 8.37 (d, J = 8.2 Hz, 1H), 7.76 (dd, J = 8.2, 1.6 Hz, 1H), 2.98 (t, J = 6.3 Hz, 2H), 2.70 (t, J = 6.7 Hz, 2H), 2.18 -2.24 (m, 2H). ^{13}C NMR (101 MHz, chloroform-d) δ = 196.2, 171.0, 158.9, 134.6, 130.8, 130.6, 130.3, 122.6, 119.1, 117.6, 110.4, 38.6, 29.0, 19.8.

Product 3ma (known compound, CAS: 106015-76-3) [7]

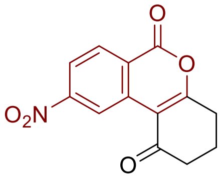

The crude product was purified by column chromatography (SiO_2, petroleum ether/ethyl acetate 10:1 to 5:1), yielding **3ma** as a white solid (22.4 mg, 43% yield).

The analytical data of compound **3ma** is in agreement with the literature report.[7] ^{1}H NMR (400 MHz, chloroform-d) δ = 9.95 (d, *J* = 2.3 Hz, 1H), 8.45 (d, *J* = 8.7 Hz, 1H), 8.30 (dd, *J* = 8.7, 2.3 Hz, 1H), 3.00 (t, *J* = 6.3 Hz, 2H), 2. 69 (t, *J* = 6.7 Hz, 2H), 2.20 - 2.26 (m, 2H). ^{13}C NMR (101 MHz, chloroform-d) δ = 196.0, 171.1, 158.7, 152.2, 135.3, 131.2, 123.9, 122.4, 121.6, 110.7, 38.5, 28.9, 19.8.

Product 3na

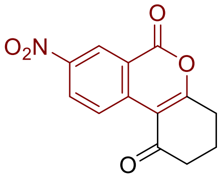

The crude product was purified by column chromatography (SiO_2, petroleum ether/ethyl acetate 10:1 to 5:1), yielding **3na** as a white solid (18.7 mg, 36% yield). ^{1}H NMR (400 MHz, chloroform-d) δ = 9.33 (d, *J* = 9.1 Hz, 1H), 9.12 (s, 1H), 8.58 (d, *J* = 7.9 Hz, 1H), 3.01 (t, *J* = 6.3 Hz, 2H), 2.71 (t, *J* = 6.6 Hz, 2H), 2.20 - 2.26 (m, 2H). ^{13}C NMR (101 MHz, chloroform-d) δ = 196.1, 172.3, 158.6, 146.9, 139.0, 129.4, 127.9, 125.2, 120.8, 110.9, 38.7, 29.2, 19.7. M.P.: 206.0 - 208.0 °C HRMS (ESI): m/z $[M+Na]^+$ calcd for $C_{13}H_9NO_5$: 282.0373; found: 282.0375.

Product 3oa (known compound, CAS: 2072821-68-0)

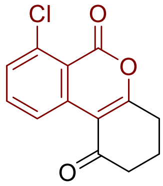

The crude product was purified by column chromatography (SiO_2, petroleum ether/ethyl acetate 10:1 to 5:1), yielding **3oa** as a white solid (39.9 mg, 80% yield).

The analytical data of compound **3oa** is in agreement with the literature report.[9] ^{1}H NMR (400 MHz, chloroform-d) δ = 9.49 (s, 1H), 7.93 (d, *J* = 8.3 Hz, 1H), 7.87 (d, *J* = 8.3 Hz, 1H), 2.94 (t, *J* = 6.3 Hz, 2H), 2.69 - 2.62 (t, *J* = 6.5 Hz, 2H), 2.15 - 2.21 (m, 2H). ^{13}C NMR (101 MHz, chloroform-d) δ = 196.4, 170.3, 160.0, 137.7, 135.1, 134.9, 130.5, 119.0, 110.4, 104.9, 38.8, 29.0, 19.8.

Product 3pa (known compound, CAS: 2759083-10-6)

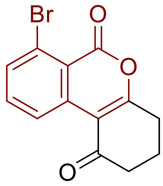

The crude product was purified by column chromatography (SiO_2, petroleum ether/ethyl acetate 4:1 to 2:1), yielding **3pa** as a white solid (48.2 mg, 82% yield).

The analytical data of compound **3pa** is in agreement with the literature report.[8] ^{1}H NMR (400 MHz, chloroform-d) δ = 9.11 (d, *J* = 8.3 Hz, 1H), 7.81 (d, *J* = 7.8 Hz, 1H), 7.55 (t, *J* =

8.1 Hz, 1H), 2.93 (t, J = 6.3 Hz, 2H), 2.71 - 2.63 (t, J = 6.7 Hz, 2H), 2.14 - 2.20 (m, 2H). ^{13}C NMR (101 MHz, chloroform-d) δ = 196.2, 170.0, 157.0, 137.1, 135.3, 135.2, 125.4, 124.6, 118.3, 111.1, 39.1, 28.9, 19.8.

Product 3qa (known compound, CAS: 2676136-67-5)

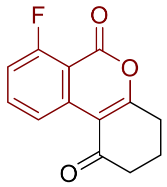

The crude product was purified by column chromatography (SiO_2, petroleum ether/ethyl acetate 10:1 to 5:1), yielding **3qa** as a white solid (34.0 mg, 73% yield).

The analytical data of compound **3la** is in agreement with the literature report.[7] ^{1}H NMR (400 MHz, chloroform-d) δ = 8.89 (d, J = 8.3 Hz, 1H), 7.73 - 7.79 (m, 1H), 7.19 - 7.24 (m, 1H), 2.94 (t, J = 6.3 Hz, 2H), 2.65 - 2.69 (t, J = 6.7 Hz, 2H), 2.15 - 2.21 (m, 2H). ^{13}C NMR (101 MHz, chloroform-d) δ = 196.3, 170.4, 162.7 (d, J = 265.3 Hz), 155.6 (d, J = 5.6 Hz), 137.1 (d, J = 10.0 Hz), 136.2, 121.8 (d, J = 4.5 Hz), 115.8 (d, J = 20.5 Hz), 110.9 (d, J = 2.8 Hz), 108.9 (d, J = 6.4 Hz), 39.0, 28.9, 19.8.

Product 3ra (known compound, CAS: 2759083-14-0)

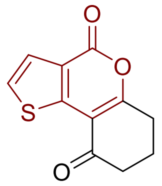

The crude product was purified by column chromatography (SiO_2, petroleum ether/ethyl acetate 10:1 to 5:1), yielding **3ra** as a white solid (26.4 mg, 60% yield).

The analytical data of compound **3ra** is in agreement with the literature report.[8] ^{1}H NMR (400 MHz, chloroform-d) δ 8.17 (d, J = 5.2 Hz, 1H), 7.84 (d, J = 5.2 Hz, 1H), 2.90 (t, J = 6.3 Hz, 2H), 2.57 (t, J = 6.6 Hz, 2H), 2.10 - 2.16 (m, 2H). ^{13}C NMR (101 MHz, chloroform-d) δ = 195.1, 170.8, 156.5, 143.7, 137.7, 126.2, 123.0, 112.4, 37.6, 28.2, 20.3.

Product 3sa (known compound, CAS: 2072821-77-1)

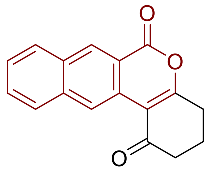

The crude product was purified by column chromatography (SiO_2, petroleum ether/ethyl acetate 10:1 to 5:1), yielding **3sa** as a white solid (41.1 mg, 78% yield). The analytical data of compound **3sa** is in agreement with the literature report.[8] ^{1}H NMR (400 MHz, chloroform-d) δ = 9.51 (s, 1H), 8.87 (s, 1H), 7.98 (t, J = 9.8 Hz, 2H), 7.65 (t, J = 7.8 Hz, 1H), 7.57 (t, J = 7.6 Hz, 1H), 2.95 (t, J = 6.3 Hz, 2H), 2.70 (t, J = 6.7 Hz, 1H), 2.17 - 2.24 (m, 2H). ^{13}C NMR (101 MHz, chloroform-d) δ = 197.2, 168.3, 136.8, 131.9, 131.7, 129.4, 129.2, 129.0, 127.9, 127.2, 125.6, 118.1, 111.6, 39.0, 29.0, 20.0.

Product 3ab (known compound, CAS: 2072821-41-9)

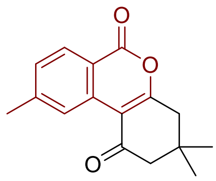

The crude product was purified by column chromatography (SiO_2, petroleum ether/ethyl acetate 10:1 to 5:1), yielding **3ab** as a white solid (38.8 mg, 76% yield). The analytical data of compound **3ab** is in agreement with the literature report.[9] ^1H NMR (400 MHz, chloroform-d) δ = 8.86 (s, 1H), 8.17 (d, J = 8.1 Hz, 1H), 7.34 (d, J = 8.0 Hz, 1H), 2.79 (s, 2H), 2.52 (s, 2H), 2.51 (s, 3H), 1.18 (s, 6H). ^{13}C NMR (101 MHz, chloroform-d) δ = 197.0, 168.0, 160.7, 147.0, 133.8, 129.6, 129.6, 125.9, 117.3, 110.5, 52.9, 42.6, 31.9, 28.1, 22.4.

Product 3ac (known compound, CAS: 2072821-51-1)

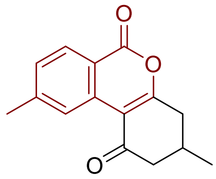

The crude product was purified by column chromatography (SiO_2, petroleum ether/ethyl acetate 10:1 to 5:1), yielding **3ac** as a white solid (20.9 mg, 43% yield). The analytical data of compound **3ac** is in agreement with the literature report.[10] ^1H NMR (400 MHz, chloroform-d) δ = 8.87 (s, 1H), 8.17 (d, J = 8.1 Hz, 1H), 7.35 (d, J = 8.1 Hz, 1H), 2.92 - 2.97 (m, 1H), 2.62 - 2.73 (m, 2H), 2.51 (s, 3H), 2.36 - 2.44 (m, 2H), 1.19 (d, J = 6.2 Hz, 3H). ^{13}C NMR (101 MHz, chloroform-d) δ = 197.0, 169.0, 160.6, 147.0, 134.0, 129.6, 129.6, 126.0, 117.4, 111.1, 47.2, 36.9, 27.7, 22.5, 20.8.

Product 3ad (known compound, CAS: 2072821-59-9)

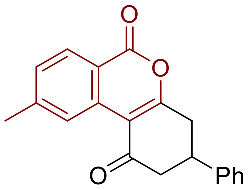

The crude product was purified by column chromatography (SiO_2, petroleum ether/ethyl acetate 10:1 to 5:1), yielding **3ad** as a white solid (60.9 mg, 93% yield). The analytical data of compound **3ad** is in agreement with the literature report.[10] ^1H NMR (400 MHz, chloroform-d) δ = 8.87 (s, 1H), 8.15 (d, J = 8.1 Hz, 1H), 7.29 - 7.41 (m, 6H), 3.51 - 3.59 (m, 1H), 3.15 – 3.12 (m, 2H), 2.91 - 2.88 (m, 2H), 2.51 (s, 3H). ^{13}C NMR (101 MHz, chloroform-d) δ = 196.1, 168.7, 160.4, 147.0, 141.4, 133.7, 129.8, 129.6, 129.0, 127.4, 126.5, 125.9, 117.2, 111.2, 46.0, 37.9, 36.3, 22.5.

Product 3ae

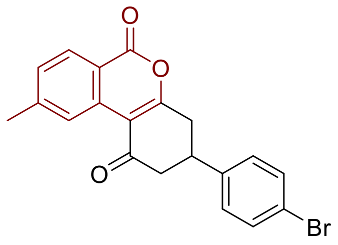

The crude product was purified by column chromatography (SiO_2, petroleum ether/ethyl acetate 10:1 to 5:1), yielding **3ae** as a white solid (51.2 mg, 66% yield). ^{1}H NMR (400 MHz, chloroform-d) δ = 8.88 (s, 1H), 8.18 (d, *J* = 8.1 Hz, 1H), 7.51 (d, *J* = 8.2 Hz, 2H), 7.37 (d, *J* = 8.1 Hz, 1H), 7.18 (d, *J* = 8.1 Hz, 2H), 3.49 - 3.57 (m, 1H), 3.13 (d, *J* = 8.3 Hz, 2H), 2.91 - 2.88 (m, 2H), 2.53 (s, 3H). ^{13}C NMR (101 MHz, chloroform-d) δ = 195.6, 168.3, 160.3, 147.2, 140.4, 133.6, 132.2, 130.1, 129.9, 128.3, 126.0, 121.3, 117.4, 111.4, 45.7, 37.5, 36.2, 22.5. M.P.: 216.0 – 218.0 °C. HRMS (EI): m/z $[M]^+$ calcd for $C_{20}H_{15}BrO_3$: 382.0210; found: 382.0199.

Product 4

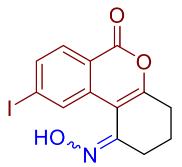

The crude product was purified by column chromatography (SiO_2, petroleum ether /ethyl acetate 3:1 to 1:1)), yielding **4** as a white solid (50.5 mg, 71% yield). ^{1}H NMR (400 MHz, DMSO-d_6) δ = 11.89 (s, 1H), 9.68 (s, 1H), 7.99 (d, *J* = 8.5 Hz, 1H), 7.87 (d, *J* = 8.4 Hz, 1H), 3.13 (t, *J* = 6.2 Hz, 2H), 2.56 (t, *J* = 6.7 Hz, 2H), 2.02 - 2.09 (m, 2H). ^{13}C NMR (101 MHz, DMSO) δ = 196.1, 158.1, 155.7, 135.2, 134.3, 134.1, 128.5, 122.6, 106.5, 102.4, 38.3, 25.4, 20.0. M.P.: 238.0 – 239.0 °C HRMS (ESI): m/z $[M+Na]^+$ calcd for $C_{13}H_{10}NO_3$: 377.9598; found: 377.9598.

Product 5

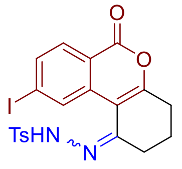

The crude product was purified by column chromatography (SiO_2, petroleum ether/ethyl acetate 3:1 to 1:1), yielding **5** as a white solid (66.7 mg, 66% yield). ^{1}H NMR (400 MHz, DMSO-d_6) δ = 10.64 (s, 1H), 9.53 (s, 1H), 7.95 (d, *J* = 8.1 Hz, 3H), 7.85 (d, *J* = 8.2 Hz, 1H), 7.39 (d, *J* = 8.0 Hz, 2H), 2.67 (t, *J* = 6.2 Hz, 2H), 2.59 (t, *J* = 6.5 Hz, 2H), 2.34 (s, 3H), 1.82 - 1.85 (m, 2H). ^{13}C NMR (101 MHz, DMSO) δ = 162.2, 160.1, 153.1, 143.6, 136.8, 136.1, 135.5, 135.2, 130.5, 129.6, 127.8, 119.5, 107.0, 104.9, 28.0, 25.6, 21.0, 18.6. M.P.: 250.0 – 252.0 °C HRMS (ESI): m/z $[M+Na]^+$ calcd for $C_{20}H_{17}IN_2O_4S$: 530.9846; found: 530.9840

References:

[1] Wang, F.; Fu, R.; Chen, J.; Rong, J.; Wang, E.; Zhang, J.; Zhang, Z. and Jiang, Y. *Chem. Commun.* **2022**, 58, 3477-3480. doi: 10.1039/d2cc00383j.

[2] Feng, J.; He, T.; Xie, Y.; Yu, Y.; Baell, J. B.; Huang, F. *Org. Biomol. Chem*. **2020**, 18, 9483-9493. doi: 10.1039/d0ob01958e.

[3] Liang, G.; Rong, J.; Sun, W.; Chen, G.; Jiang, Y.; Loh, T. P. *Org. Lett*. **2018**, 20, 7326-7331. doi: 10.1021/acs.orglett.8b03284.

[4] Moriarty, R., M.; Tyagi, S.; Ivanov, D.; Constantinescu, M. *J. Am. Chem. Soc*. **2008**, 130, 7564-7565. doi: 10.1021/ja802735f.

[5] Li, R.; Hou, Y. X.; Xu, J. H.; Gao, Y.; Hu, X.-Q. *Org. Chem. Front.* **2022**, 9, 2181-2186. doi: 10.1039/d2qo00144f.

[6] Mayakrishnan, S.; Tamizmani, M.; Maheswari, N. U. *Chem. Commun*. **2020**, 56, 15462-15465. doi: 10.1039/d0cc06038k.

[7] Dong, Z Z.; Li, P F.; Li, X. W.; Liu, B. X. *Chin. J. Chem.* **2021**, 39, 2489-2494. doi: 10.1002/cjoc.202100203.

[8] Kumar, S.; Nunewar, S.; Kanchupalli, V. Asian *J. Org. Chem*. **2022,** 11, e202100689. doi:10.1002/ajoc.202100689.

[9] Yang, C.; He, X. W.; Zhang, L. L.; Han, G.; Zuo, Y. P.; Shang, Y. J. *J. Org. Chem.* **2017**, 82, 2081-2088. doi: 10.1021/acs.joc.6b02906.

[10] He, X. W.; Han, G.; Zuo, Y. P.; Shang, Y. J. *Tetrahedron.* **2018**, 74, 7082-7088. doi: 10.1016/j.tet.2018.10.045.

4. NMR Spectra of products 3aa–sa, 3ab–ae, 4 and 5

^{1}H NMR (400 MHz, CDCl3) and ^{13}C NMR (100 MHz, CDCl3) spectra of product 3aa

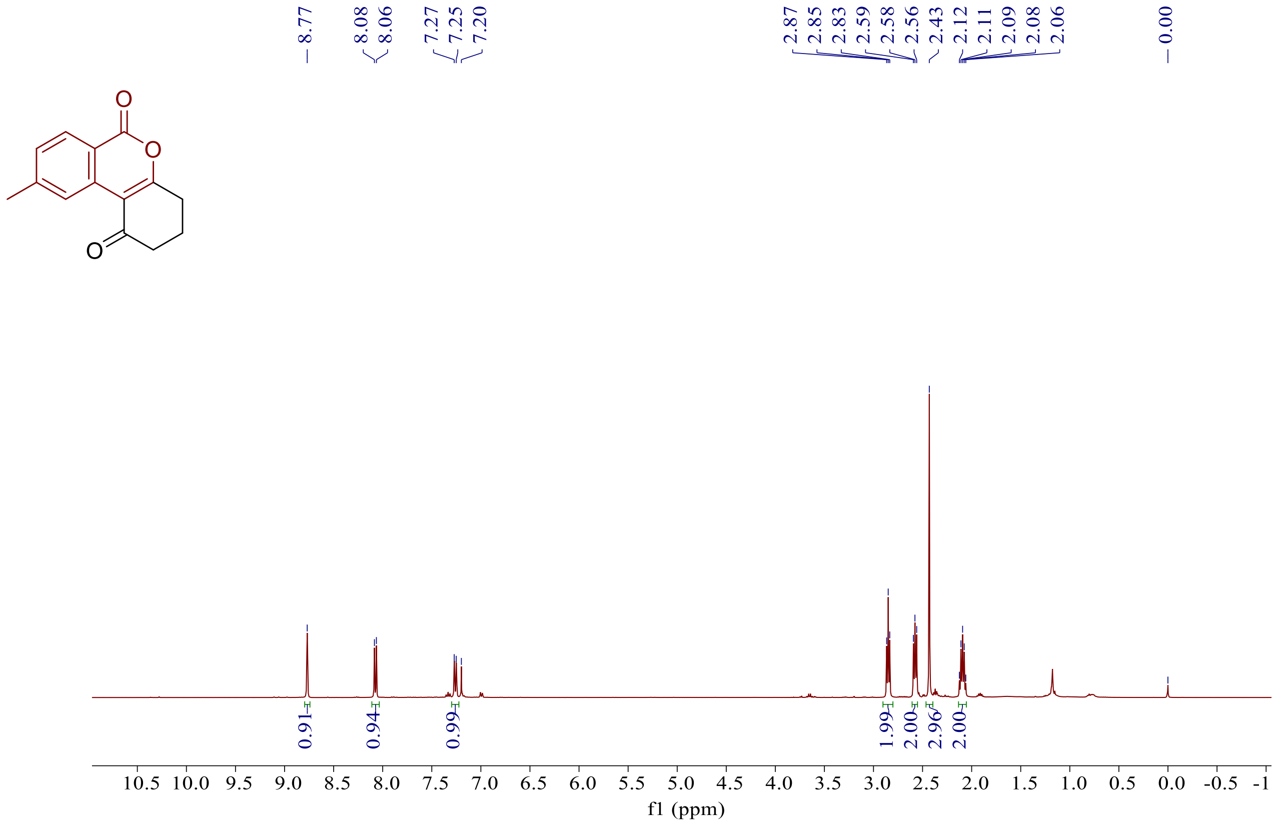

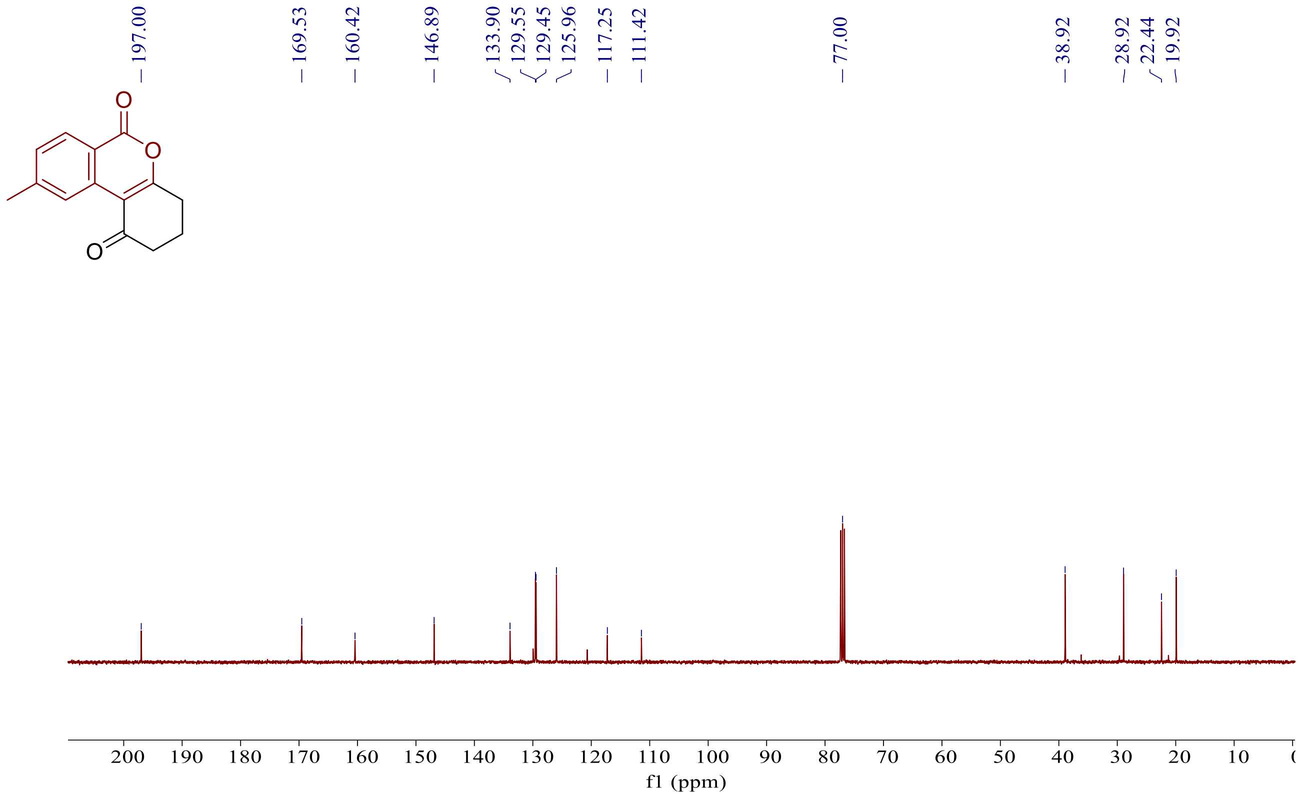

^{1}H NMR (400 MHz, CDCl3) and ^{13}C NMR (100 MHz, CDCl3) spectra of product 3ba

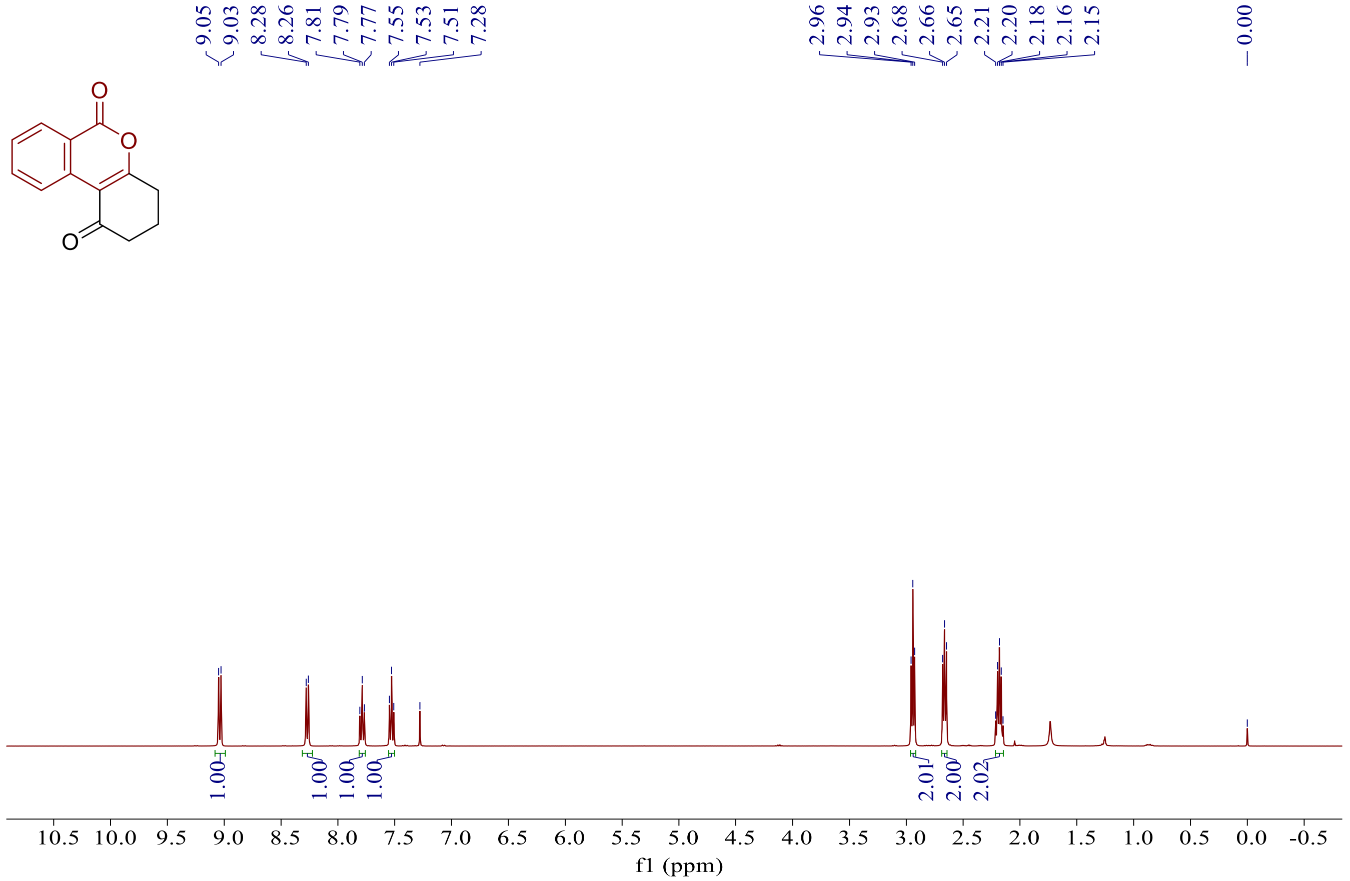

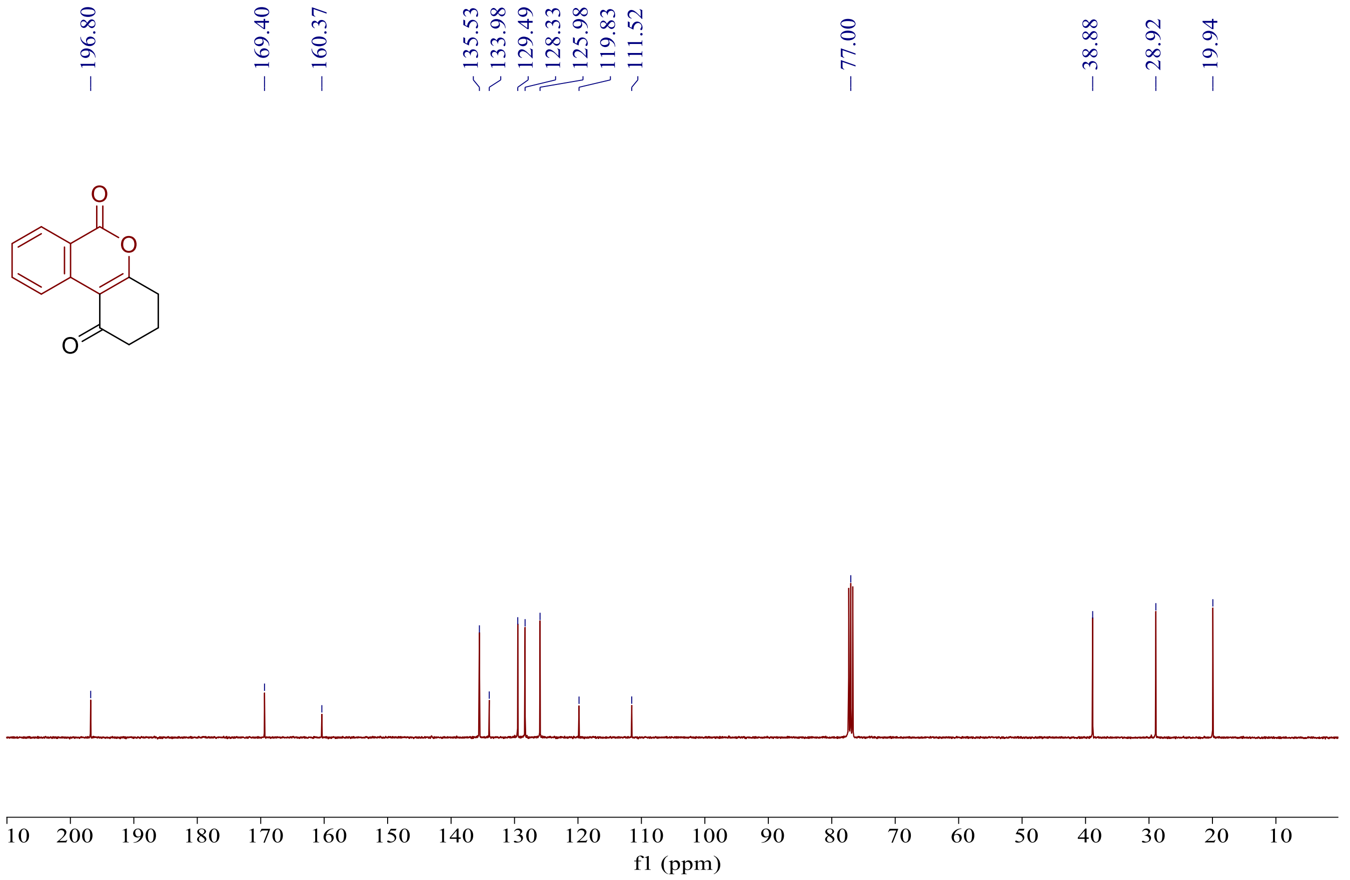

^{1}H NMR (400 MHz, CDCl3) and ^{13}C NMR (100 MHz, CDCl3) spectra of product 3ca

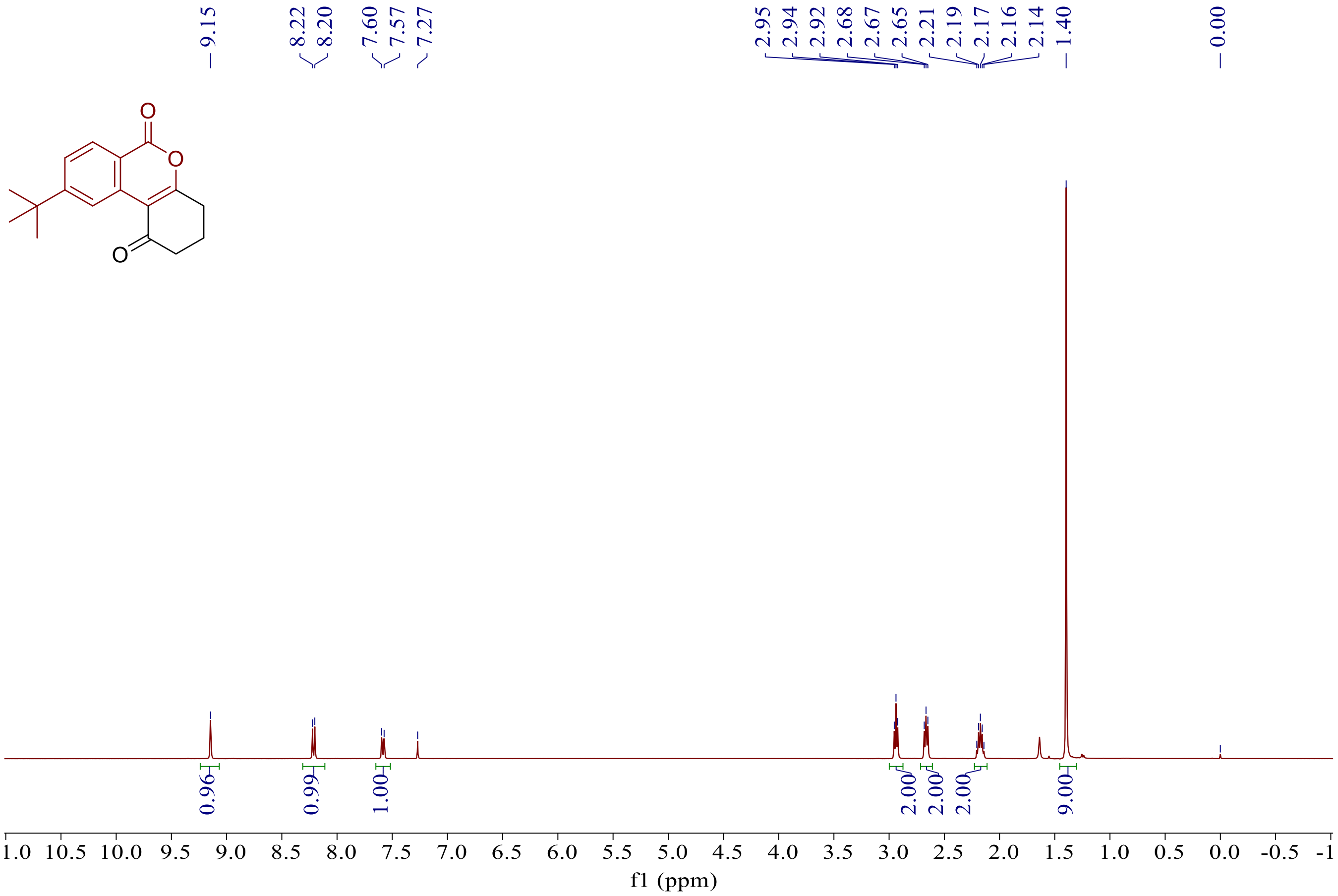

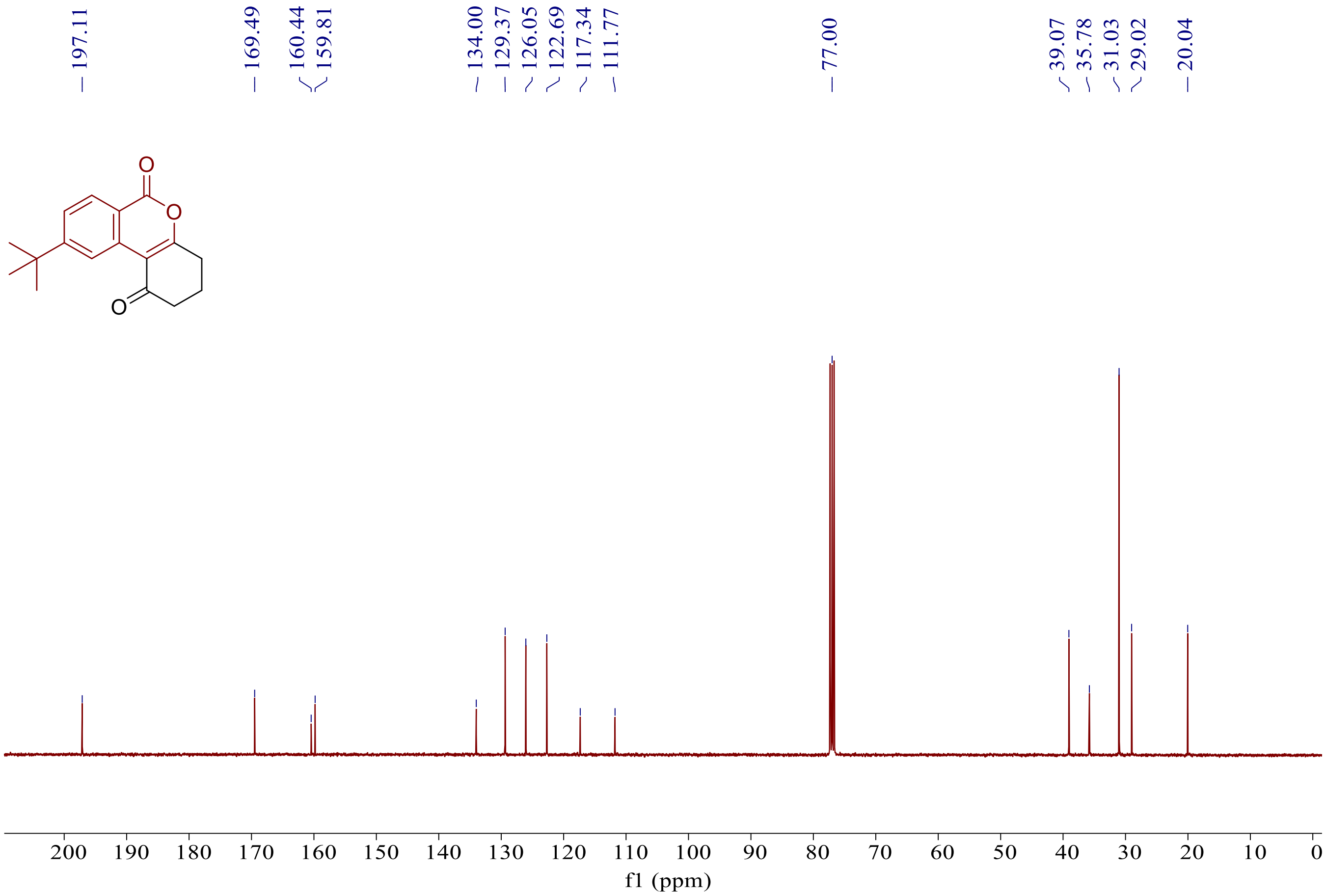

^{1}H NMR (400 MHz, CDCl$_3$) and ^{13}C NMR (100 MHz, CDCl$_3$) spectra of product 3da

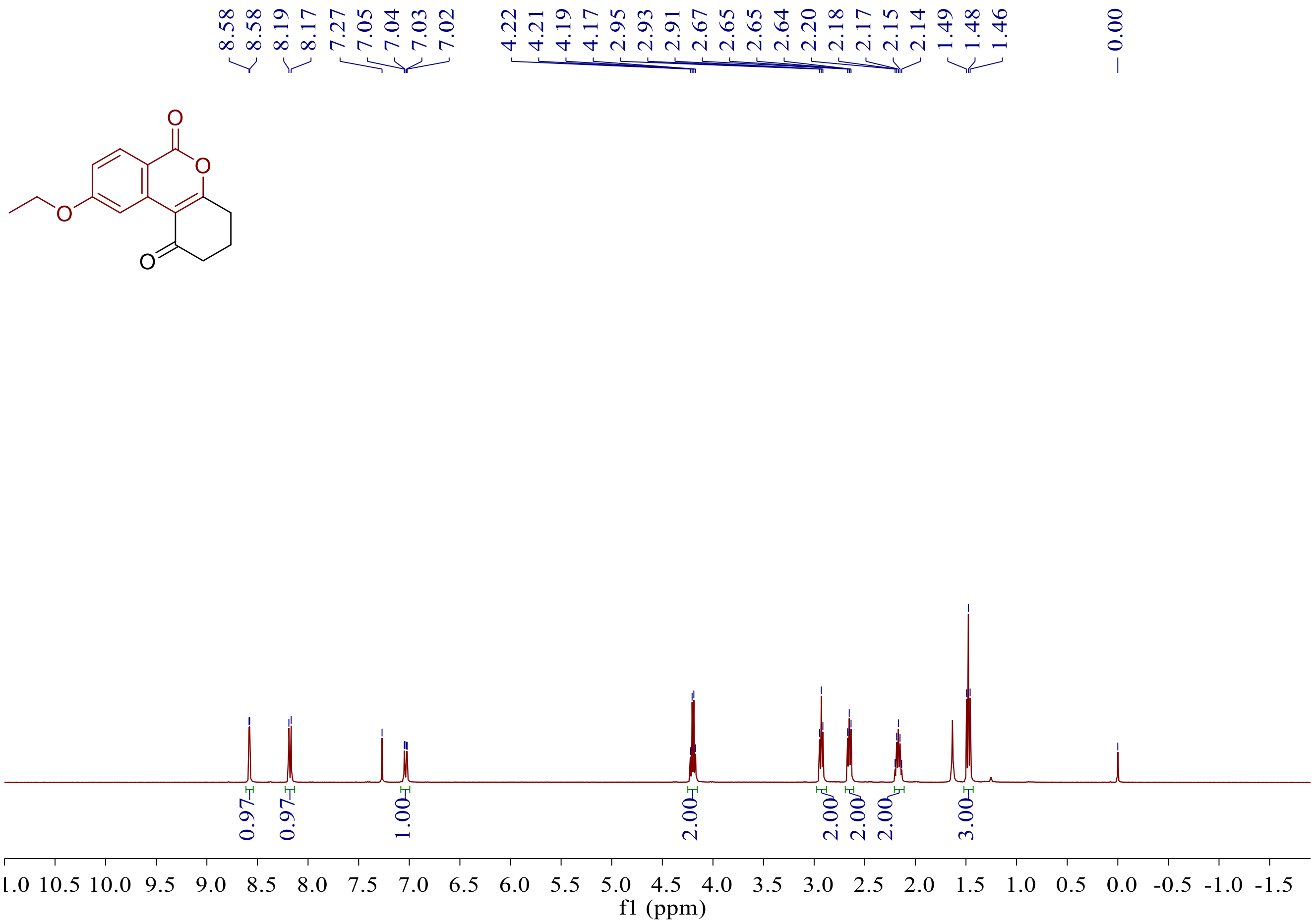

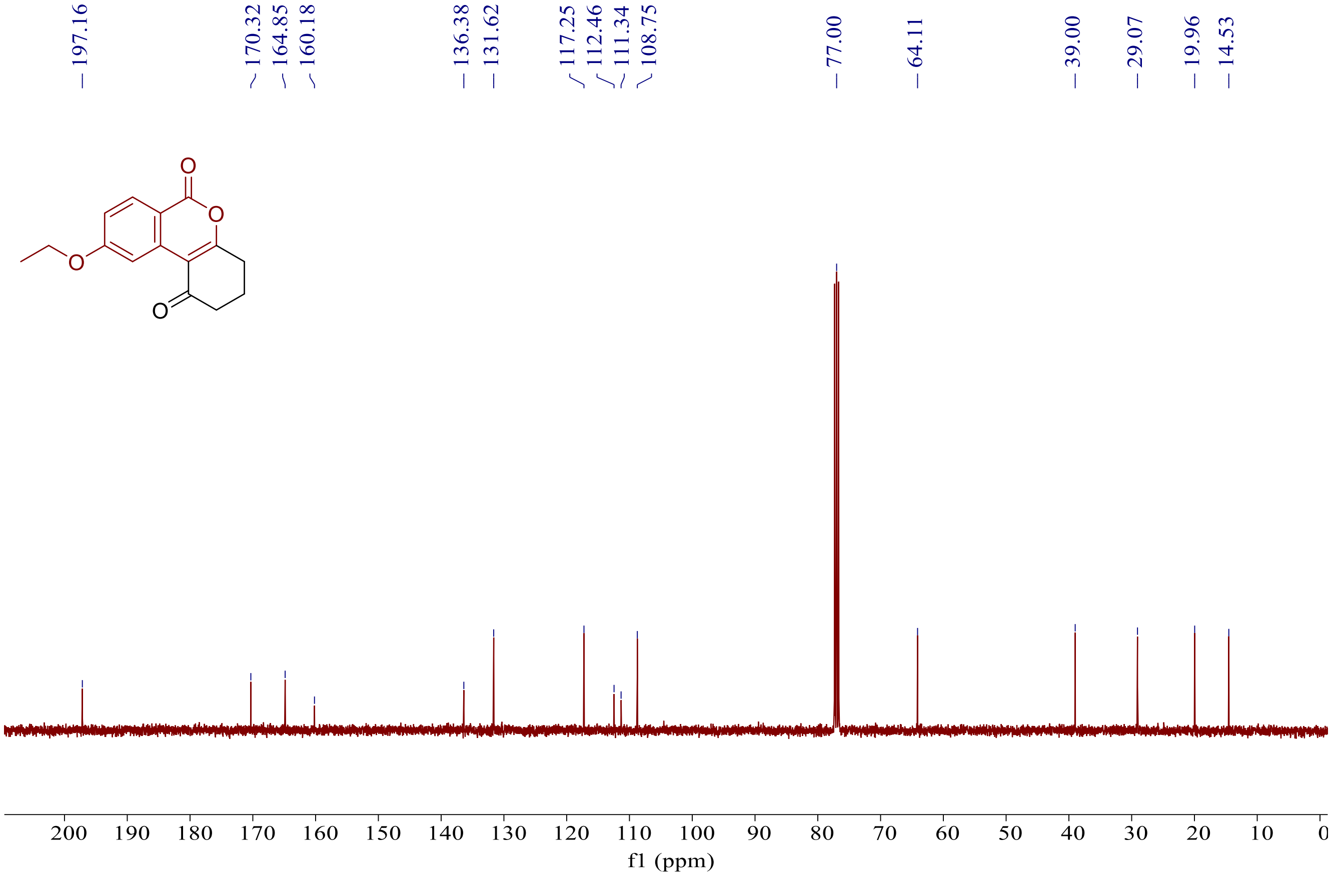

^{1}H NMR (400 MHz, CDCl3) and ^{13}C NMR (100 MHz, CDCl3) spectra of product 3ea

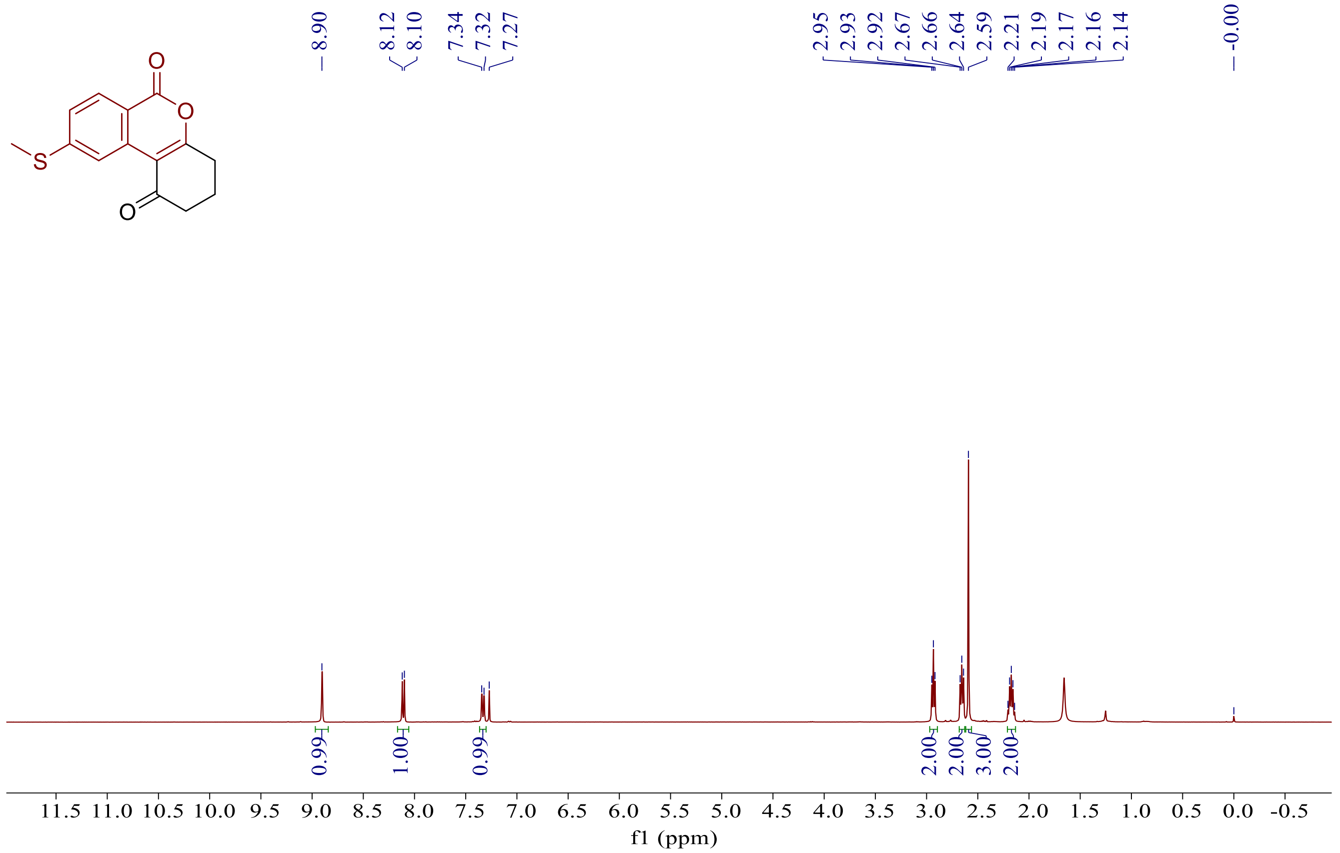

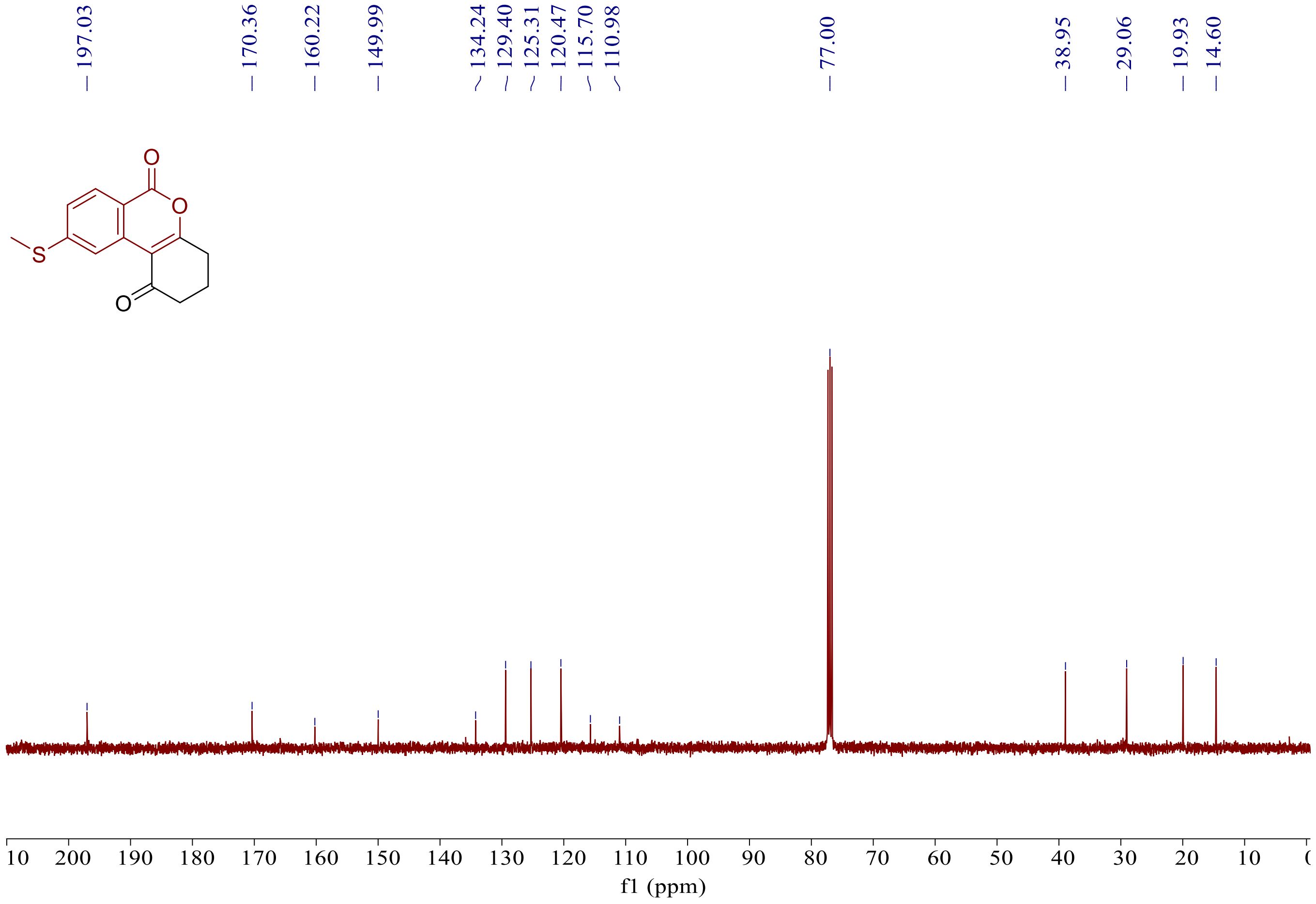

^{1}H NMR (400 MHz, CDCl3) and ^{13}C NMR (100 MHz, CDCl3) spectra of product 3fa

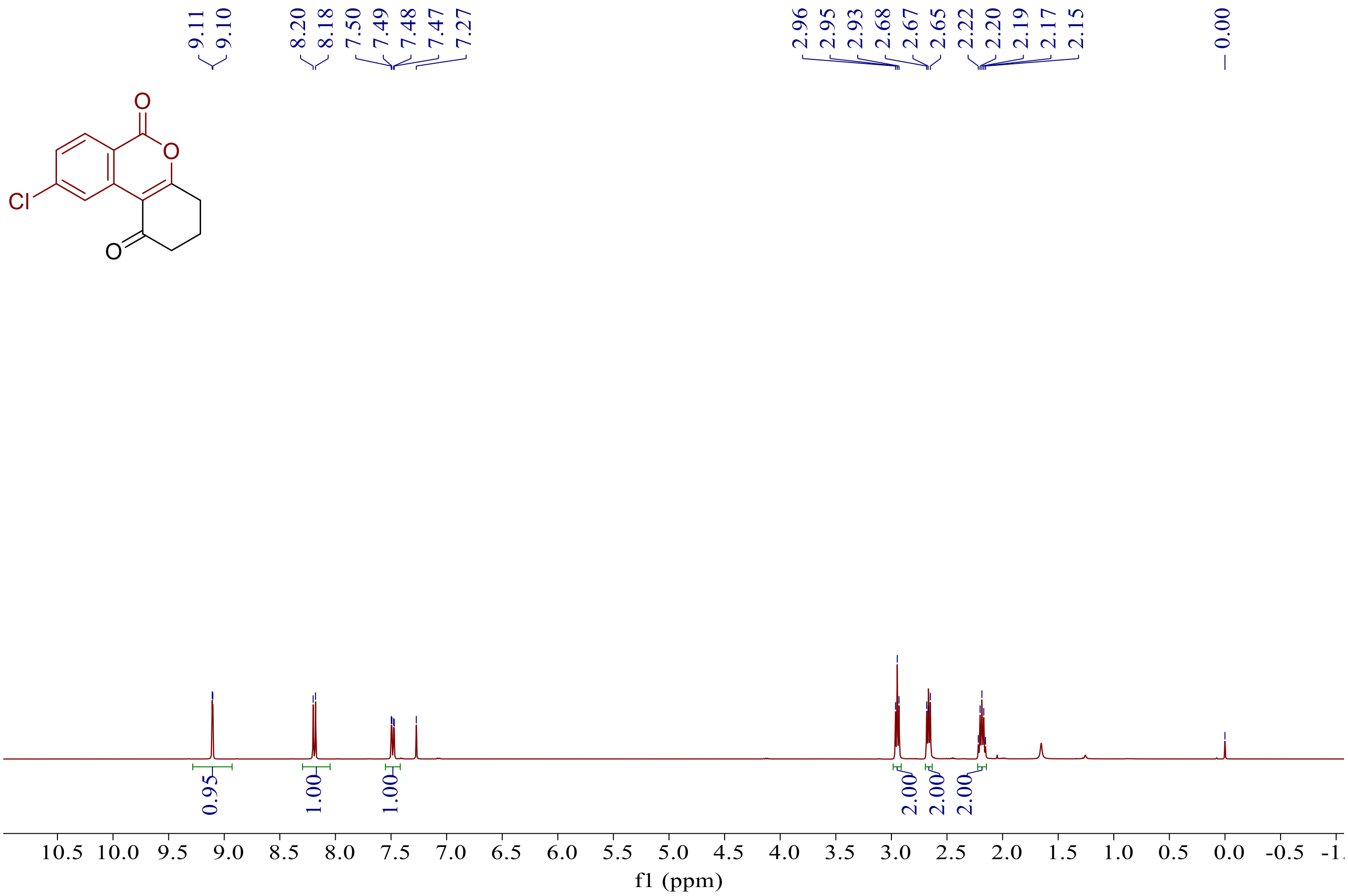

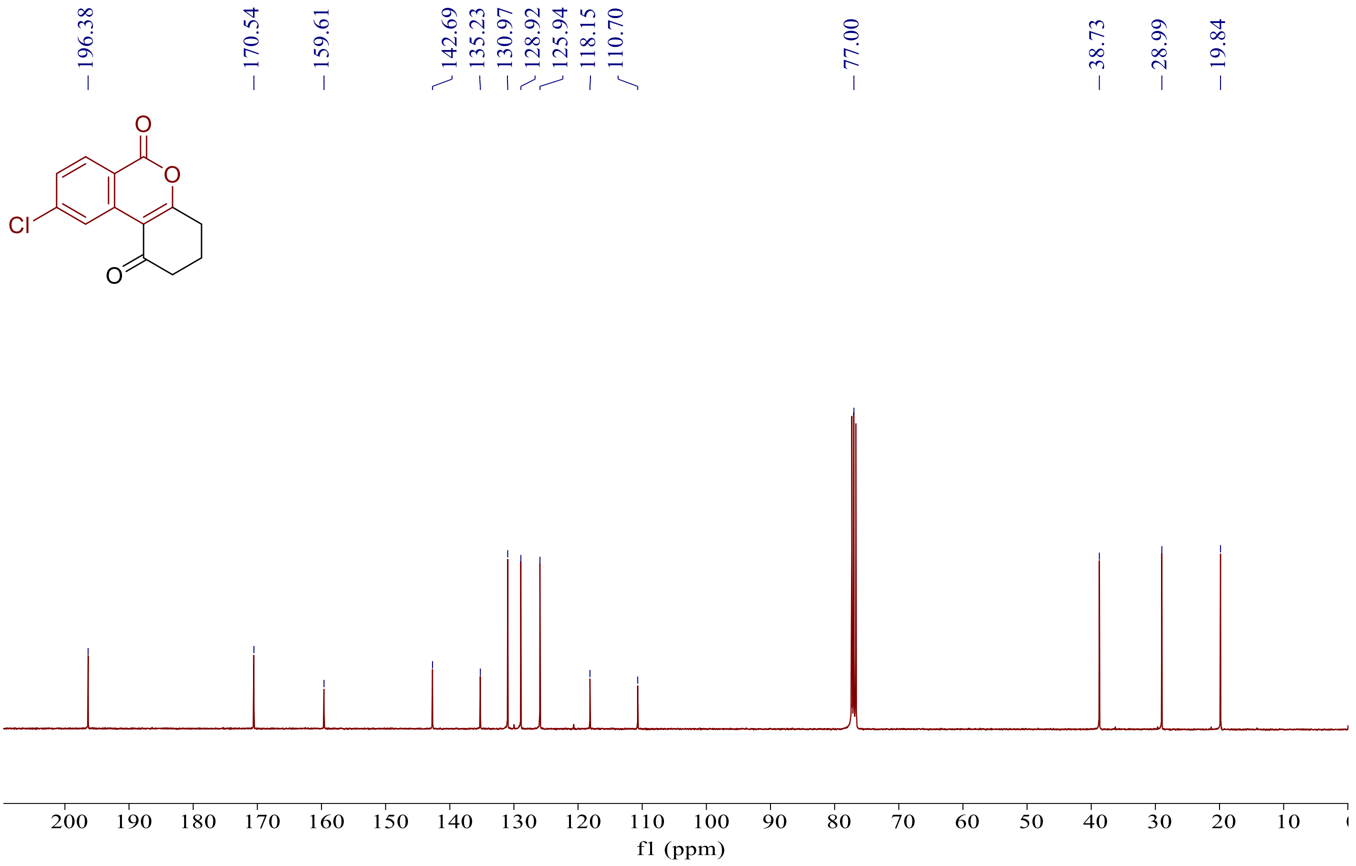

^{1}H NMR (400 MHz, $CDCl_3$) and ^{13}C NMR (100 MHz, $CDCl_3$) spectra of product 3ga

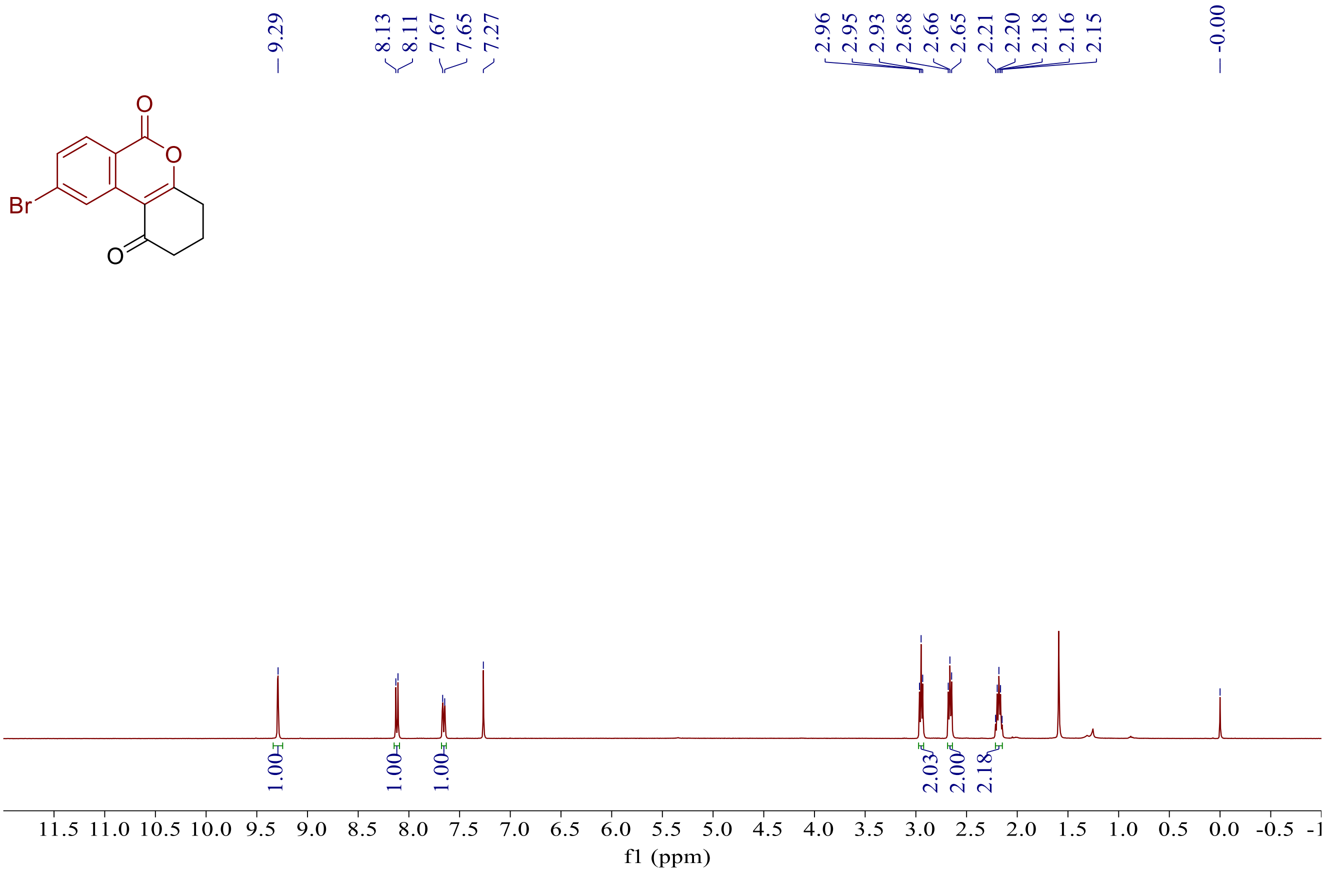

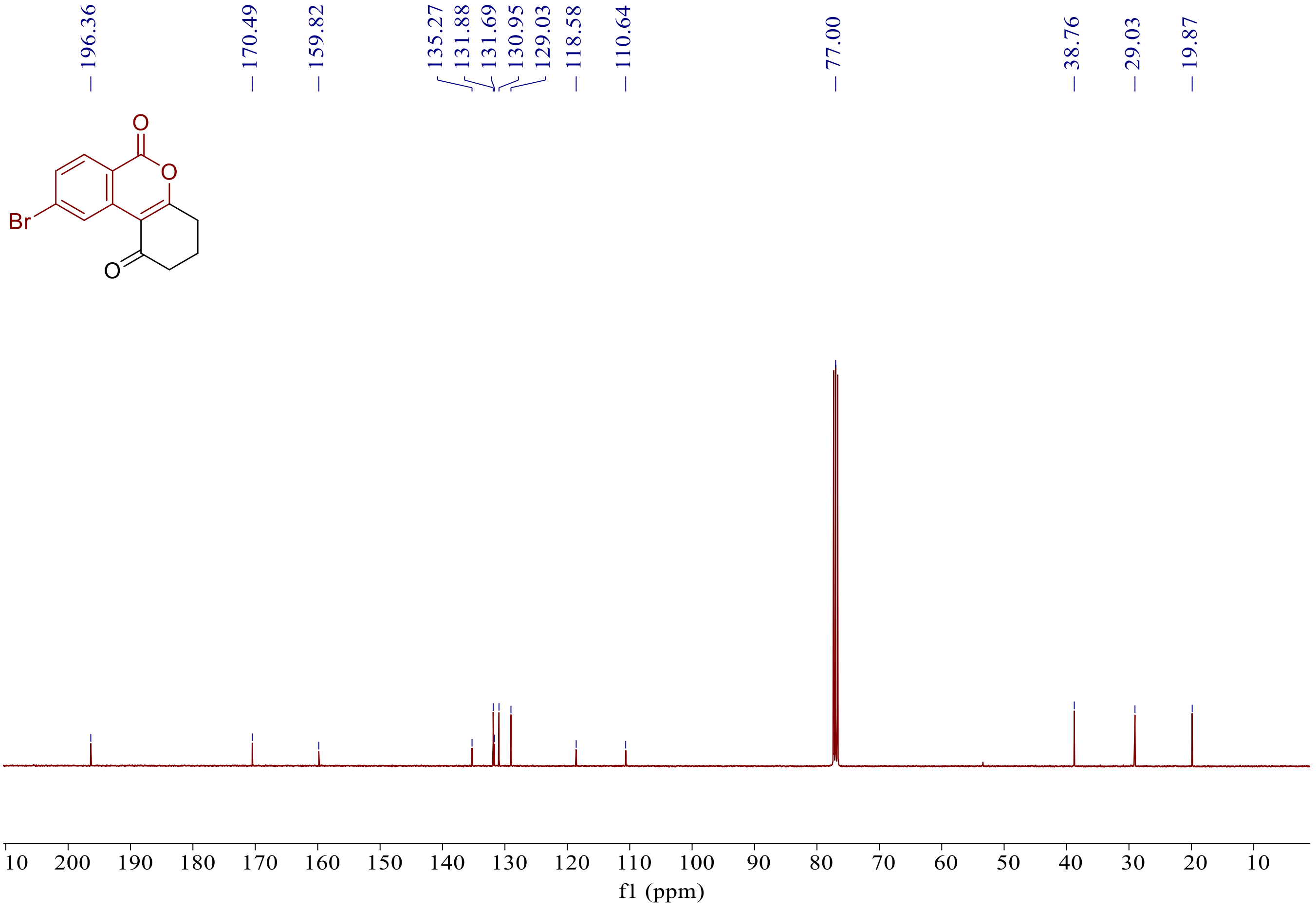

^{1}H NMR (400 MHz, $CDCl_3$) and ^{13}C NMR (100 MHz, $CDCl_3$) spectra of product 3ha

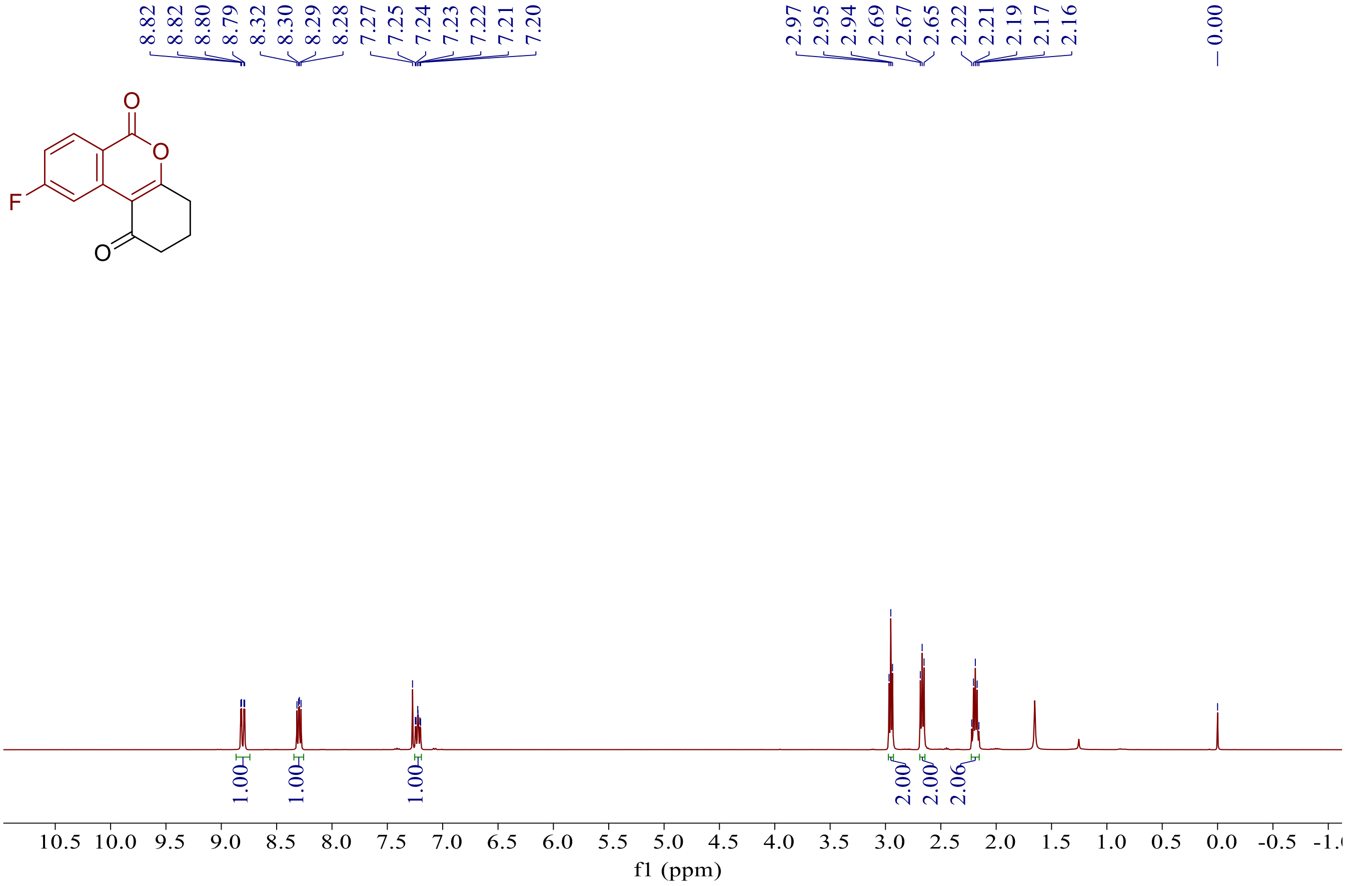

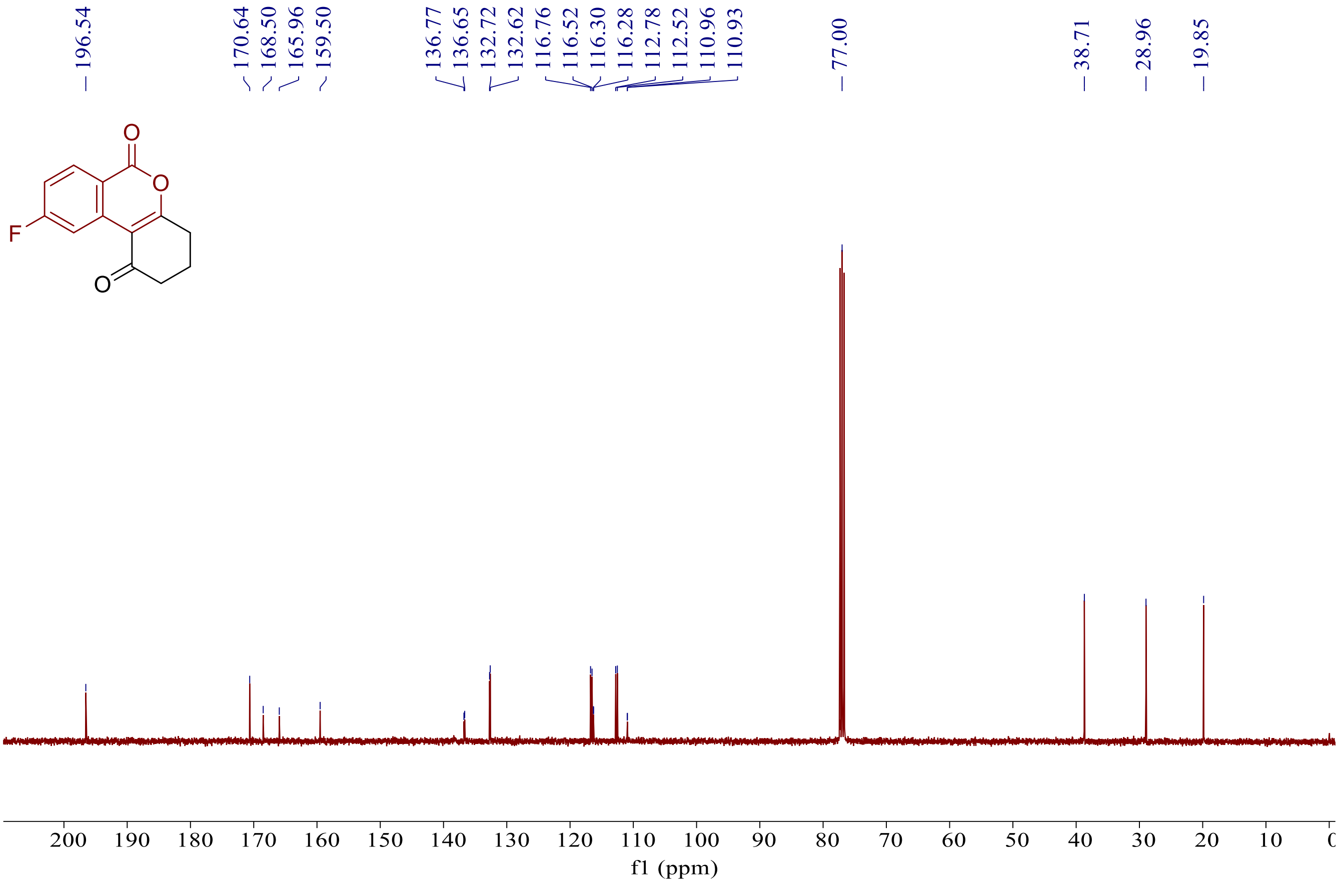

^{1}H NMR (400 MHz, CDCl3) and ^{13}C NMR (100 MHz, CDCl3) spectra of product 3ia

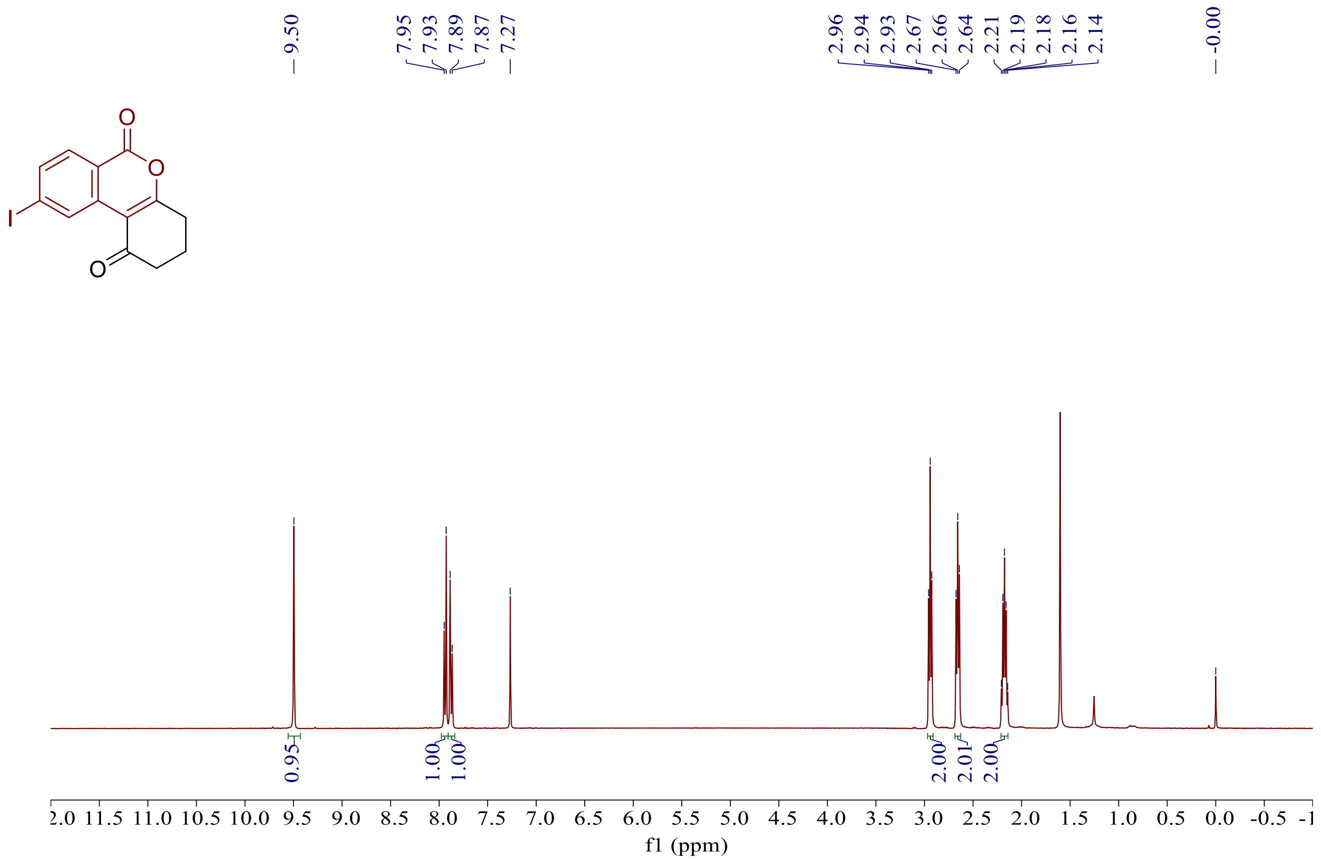

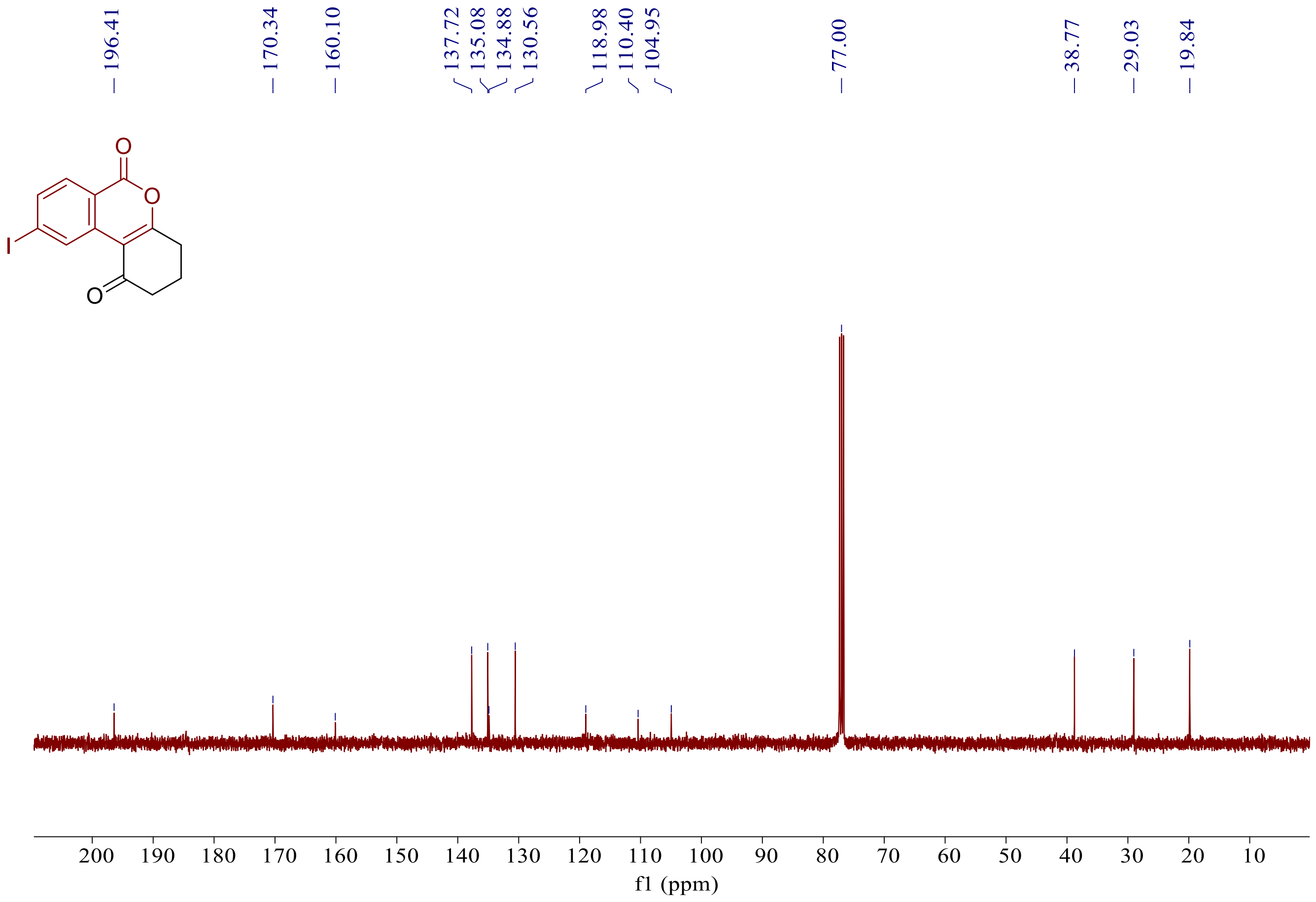

^{1}H NMR (400 MHz, $CDCl_3$) and ^{13}C NMR (100 MHz, $CDCl_3$) spectra of product 3ja

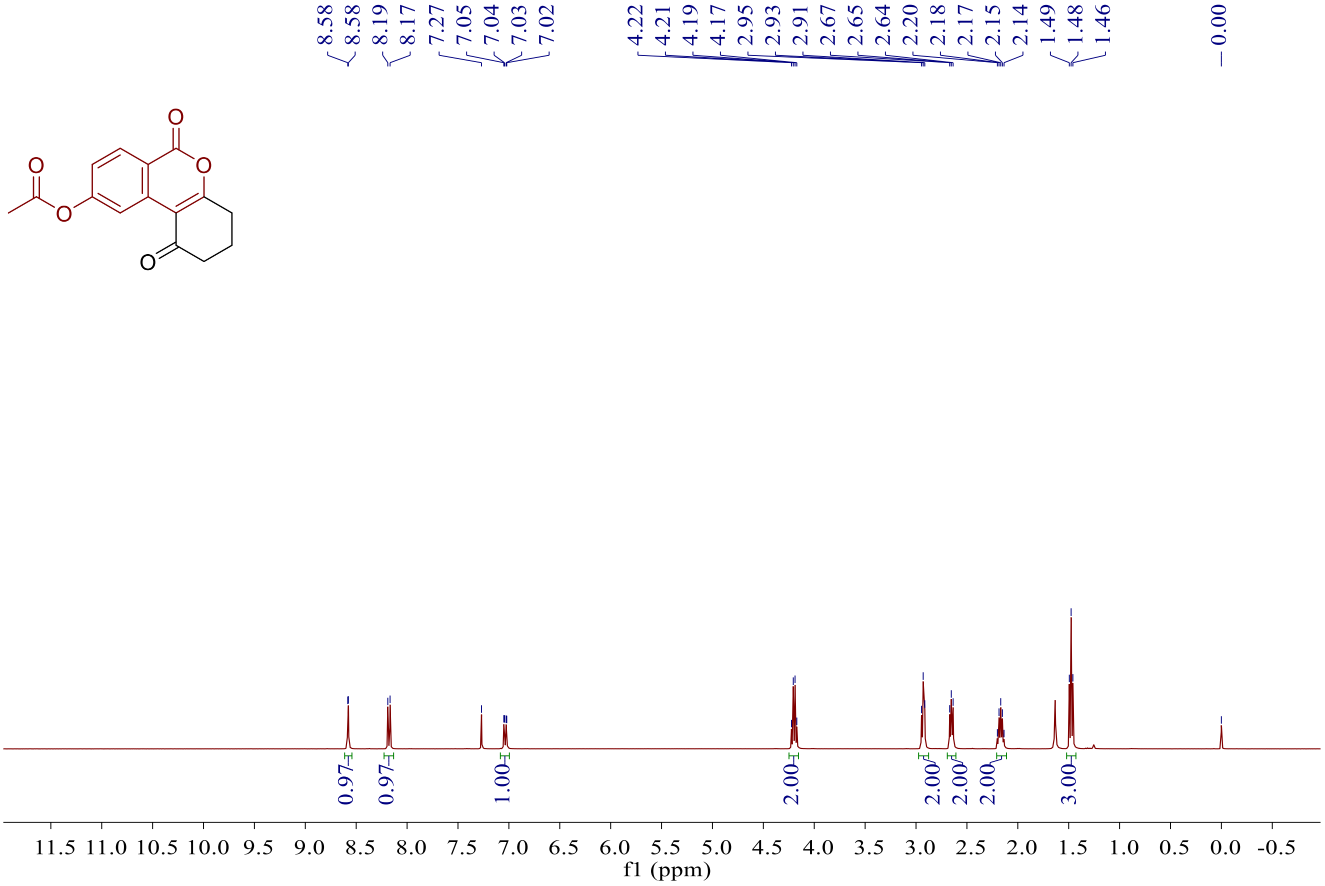

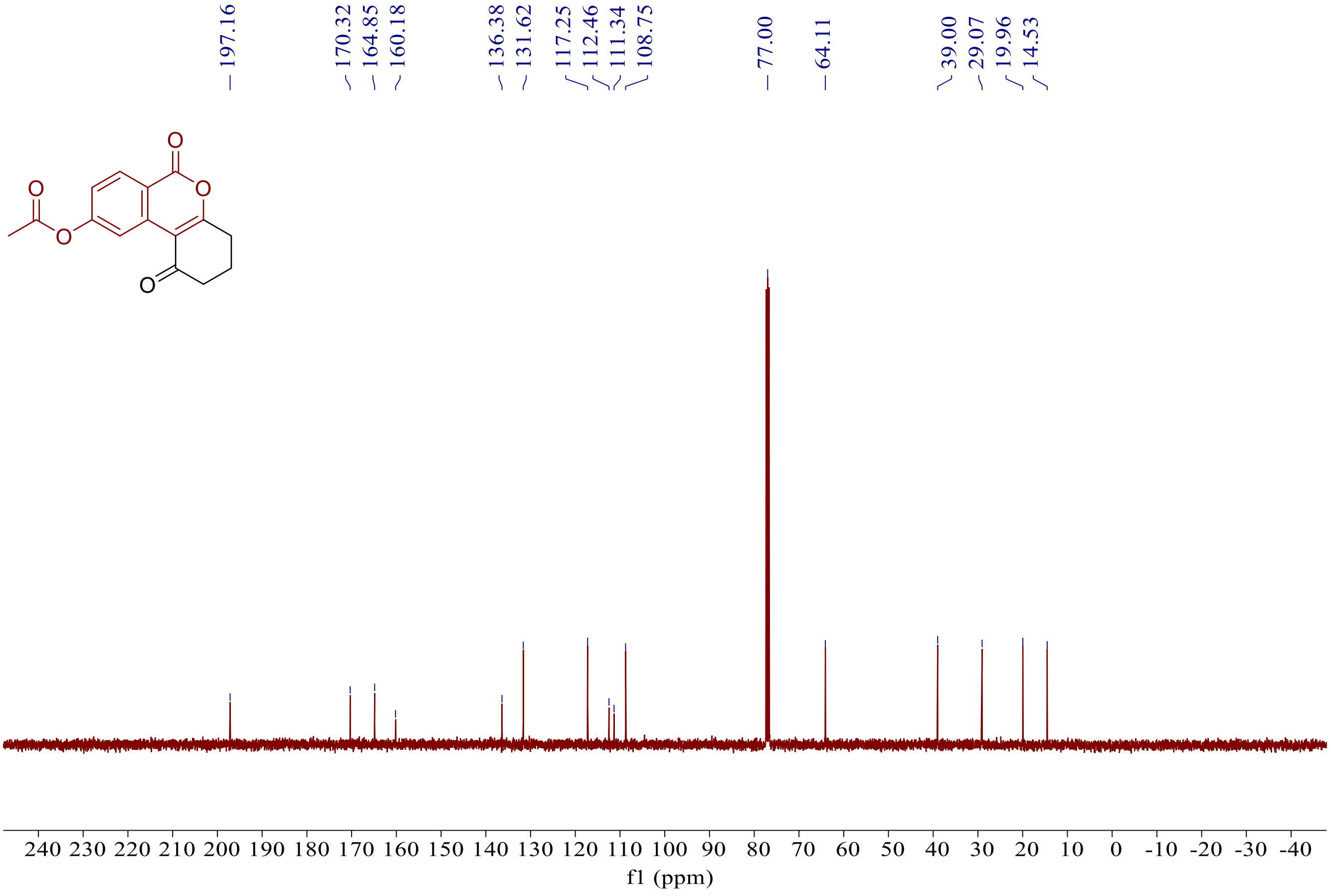

^{1}H NMR (400 MHz, $CDCl_3$) and ^{13}C NMR (100 MHz, $CDCl_3$) spectra of product 3ka

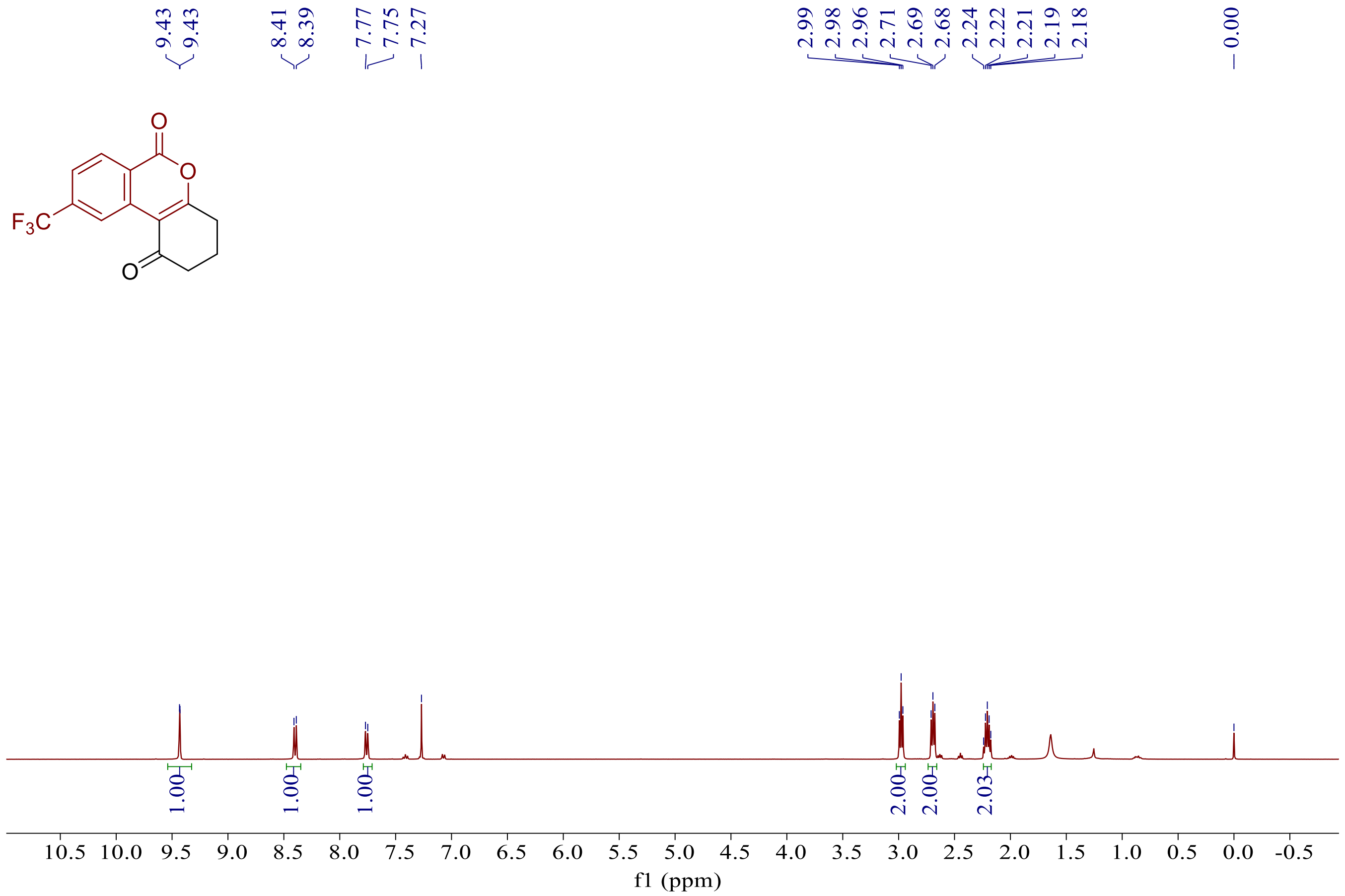

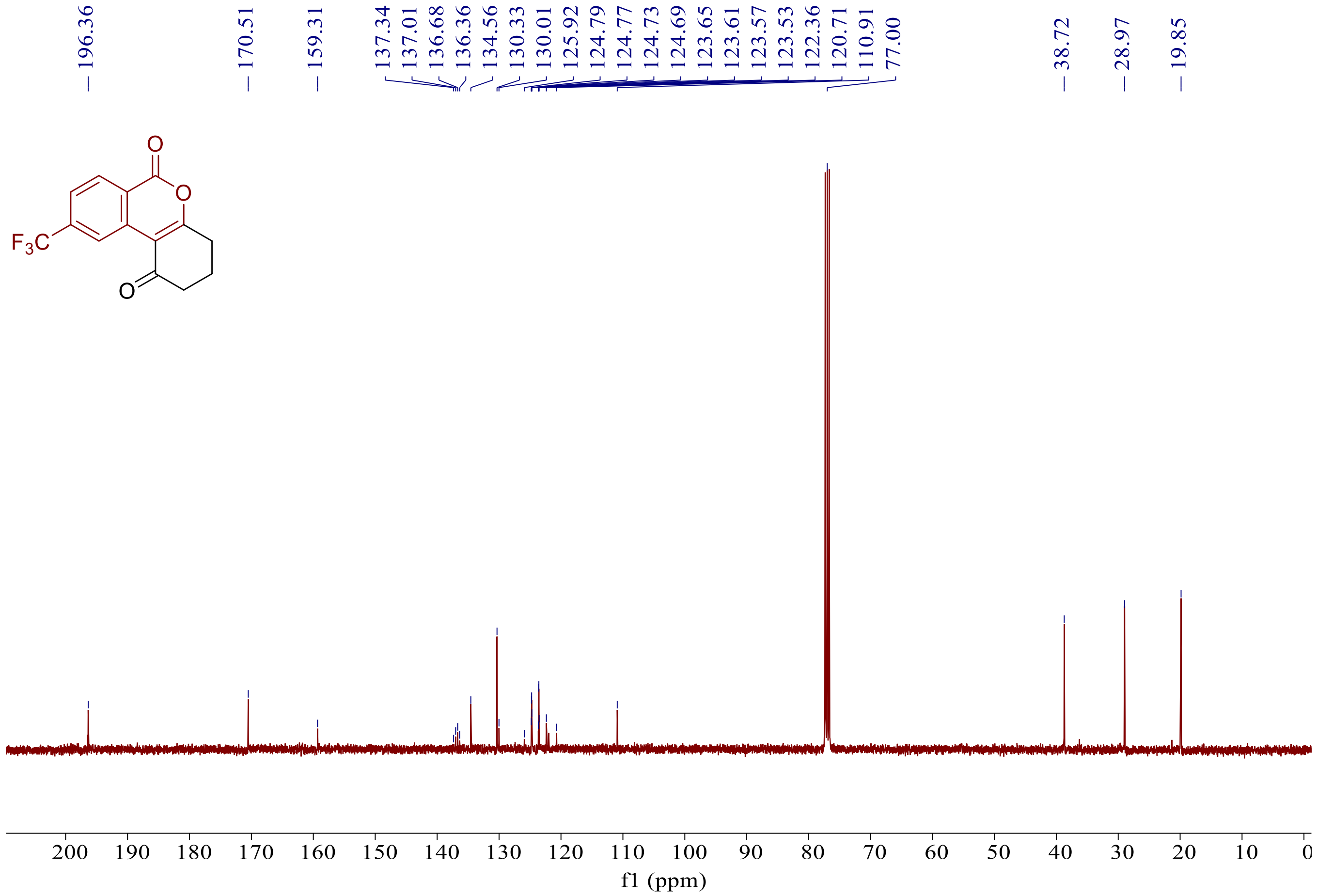

^{1}H NMR (400 MHz, CDCl$_3$) and ^{13}C NMR (100 MHz, CDCl$_3$) spectra of product 3la

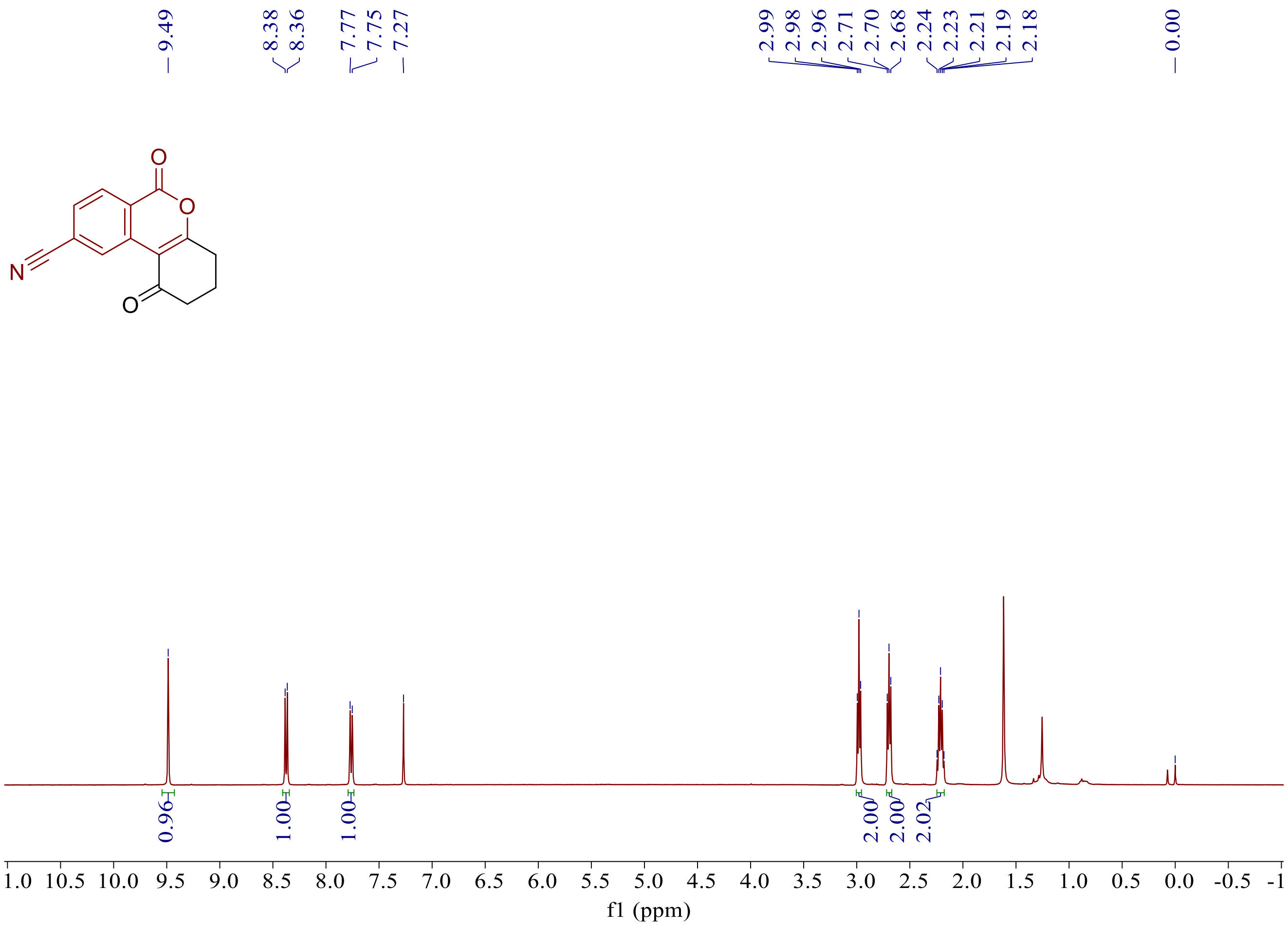

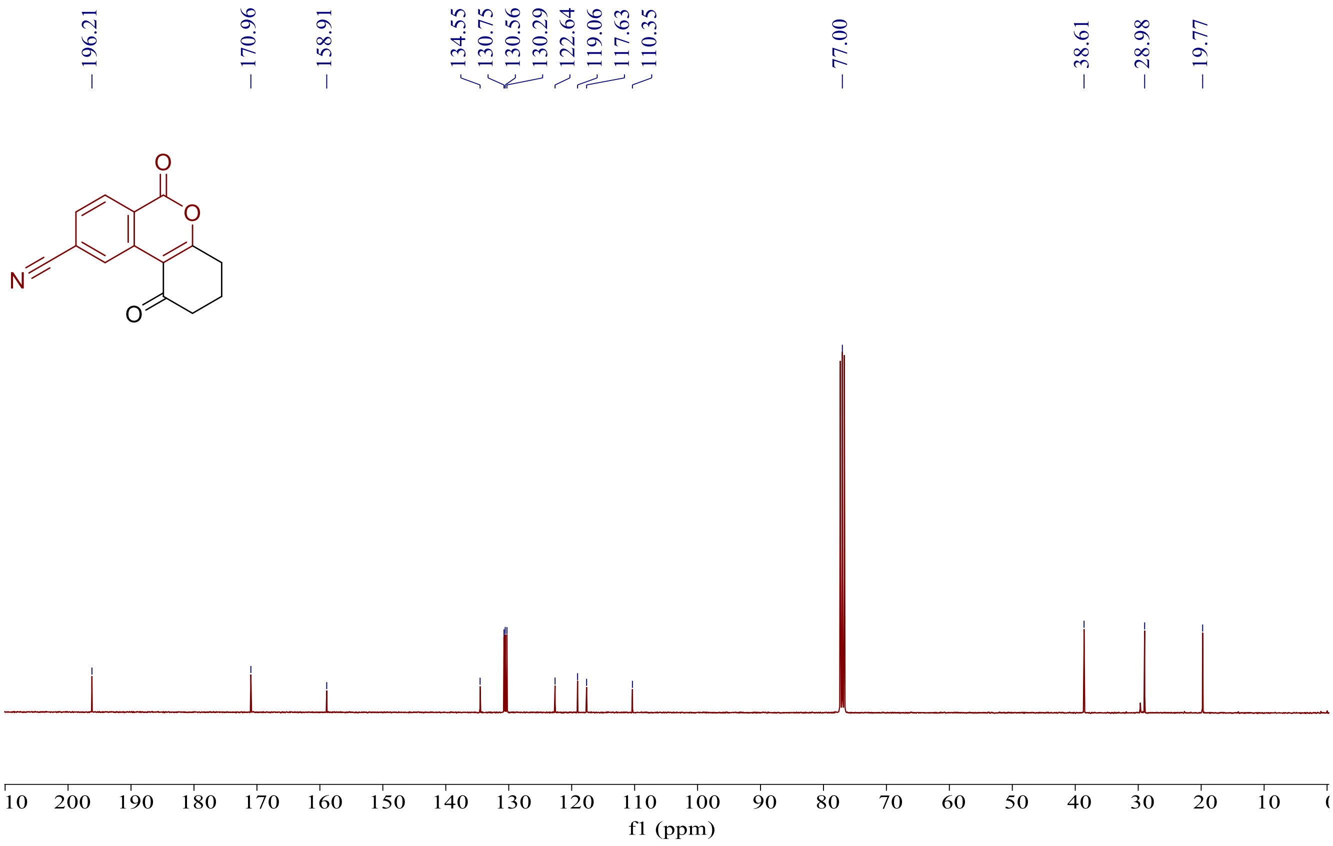

^{1}H NMR (400 MHz, CDCl3) and ^{13}C NMR (100 MHz, CDCl3) spectra of product 3ma

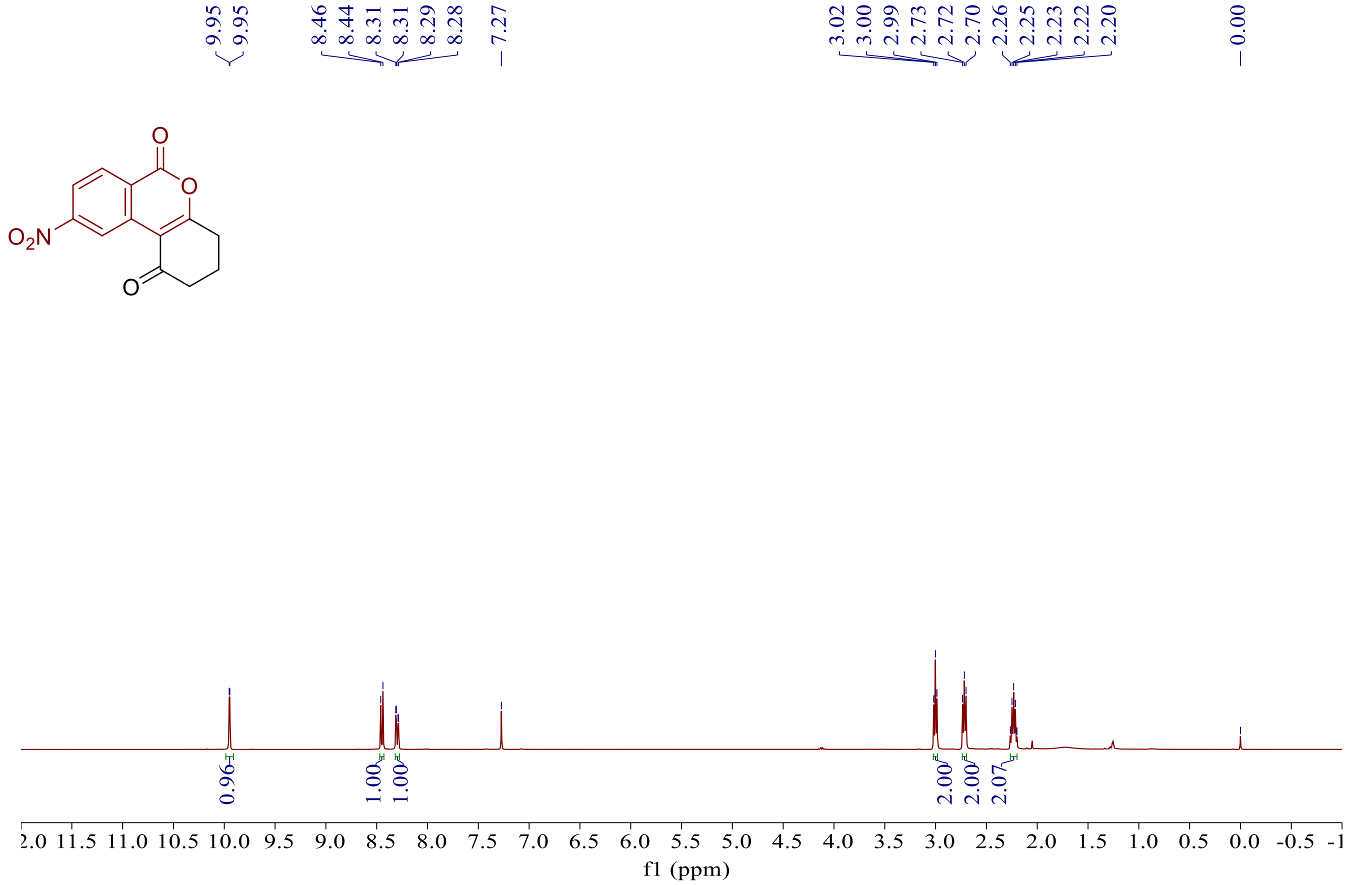

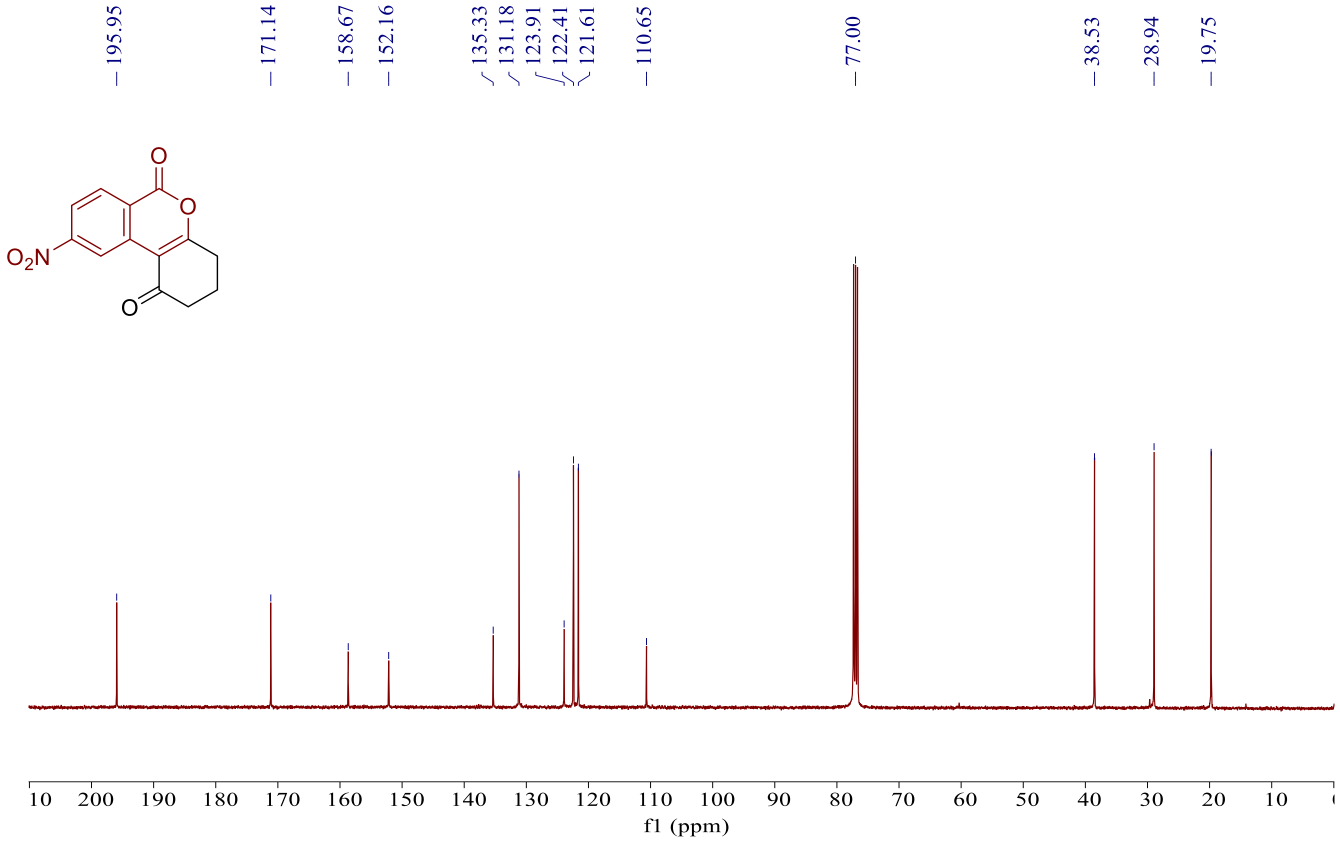

^{1}H NMR (400 MHz, CDCl3) and ^{13}C NMR (100 MHz, CDCl3) spectra of product 3na

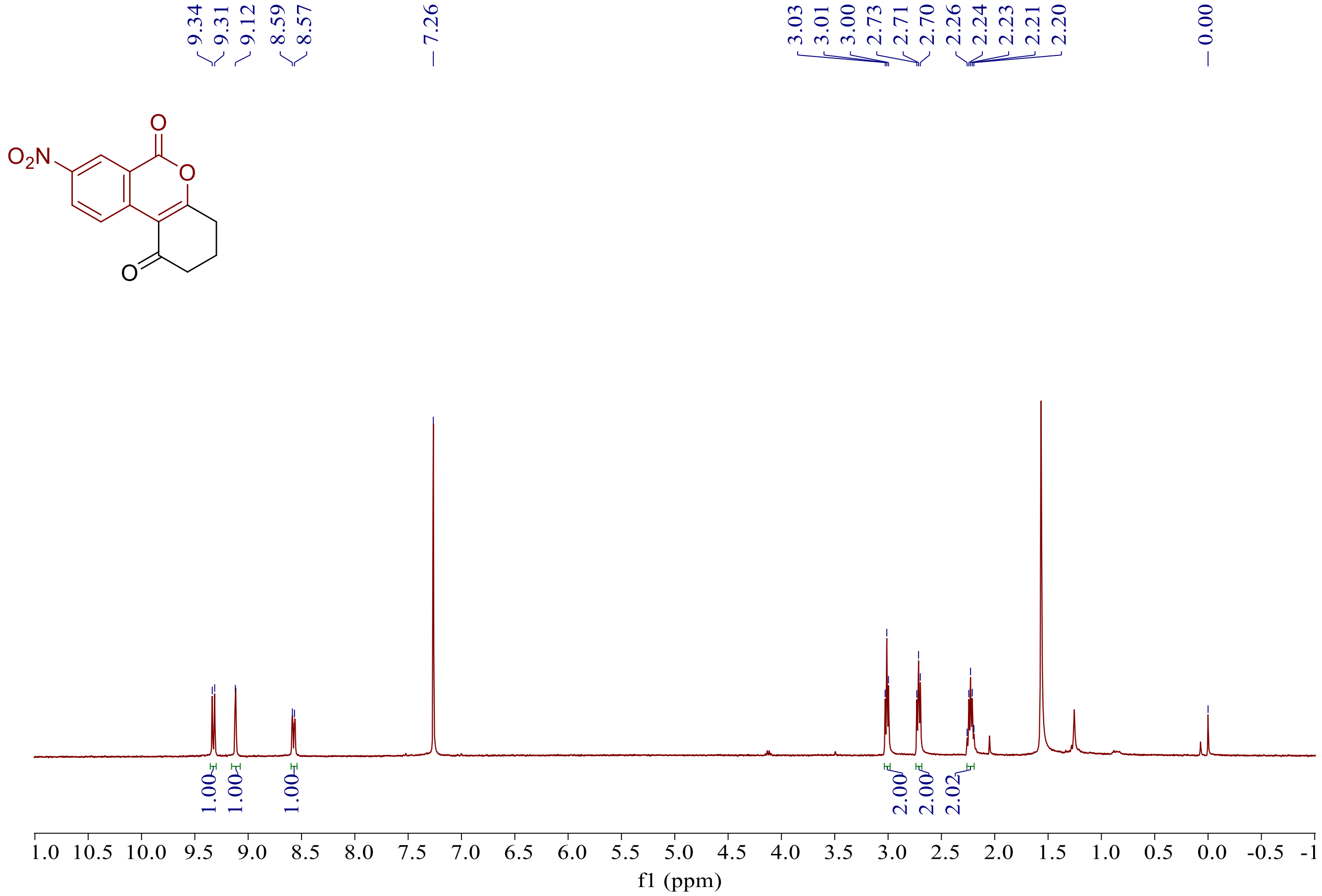

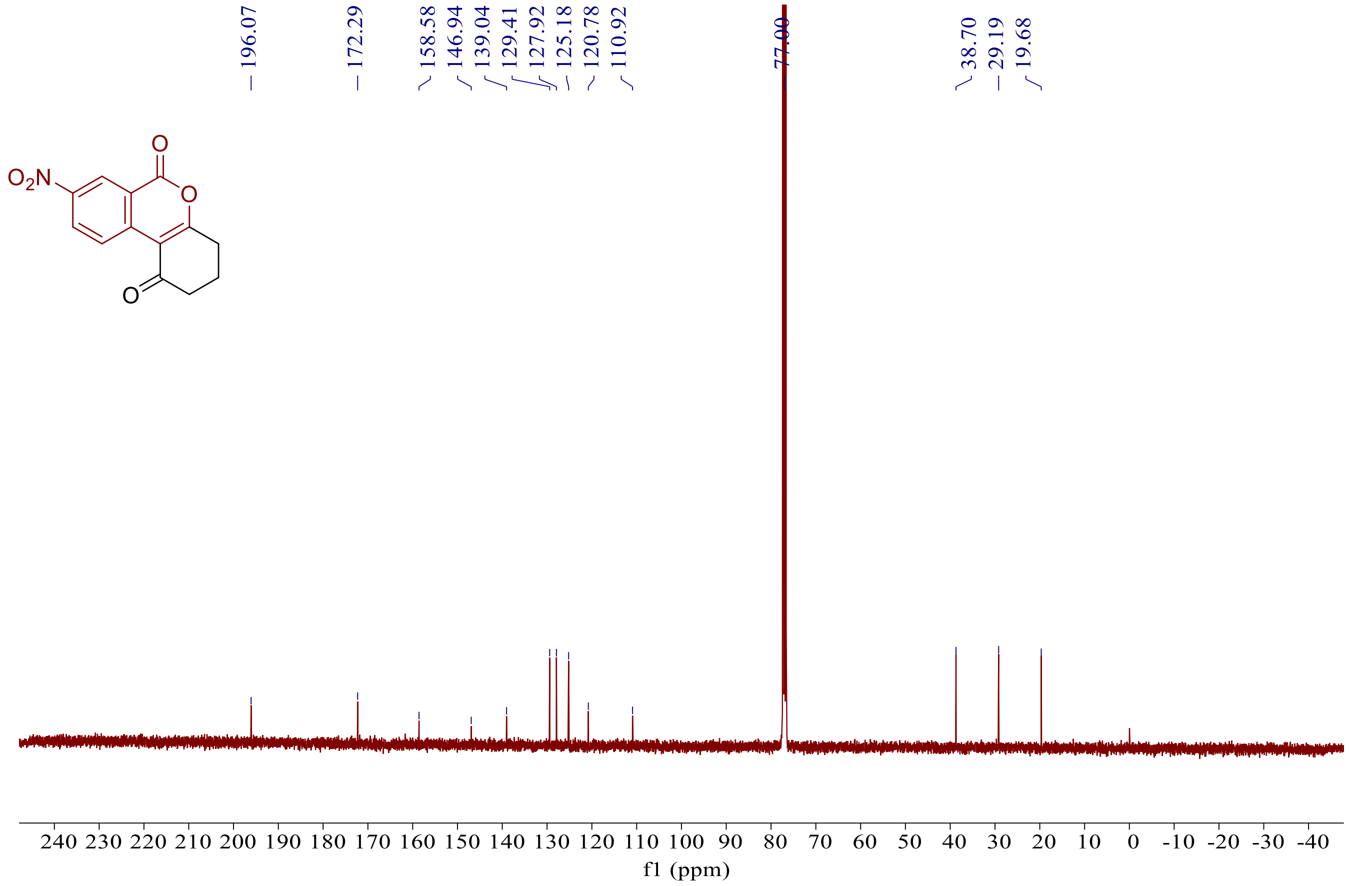

^{1}H NMR (400 MHz, $CDCl_3$) and ^{13}C NMR (100 MHz, $CDCl_3$) spectra of product 3oa

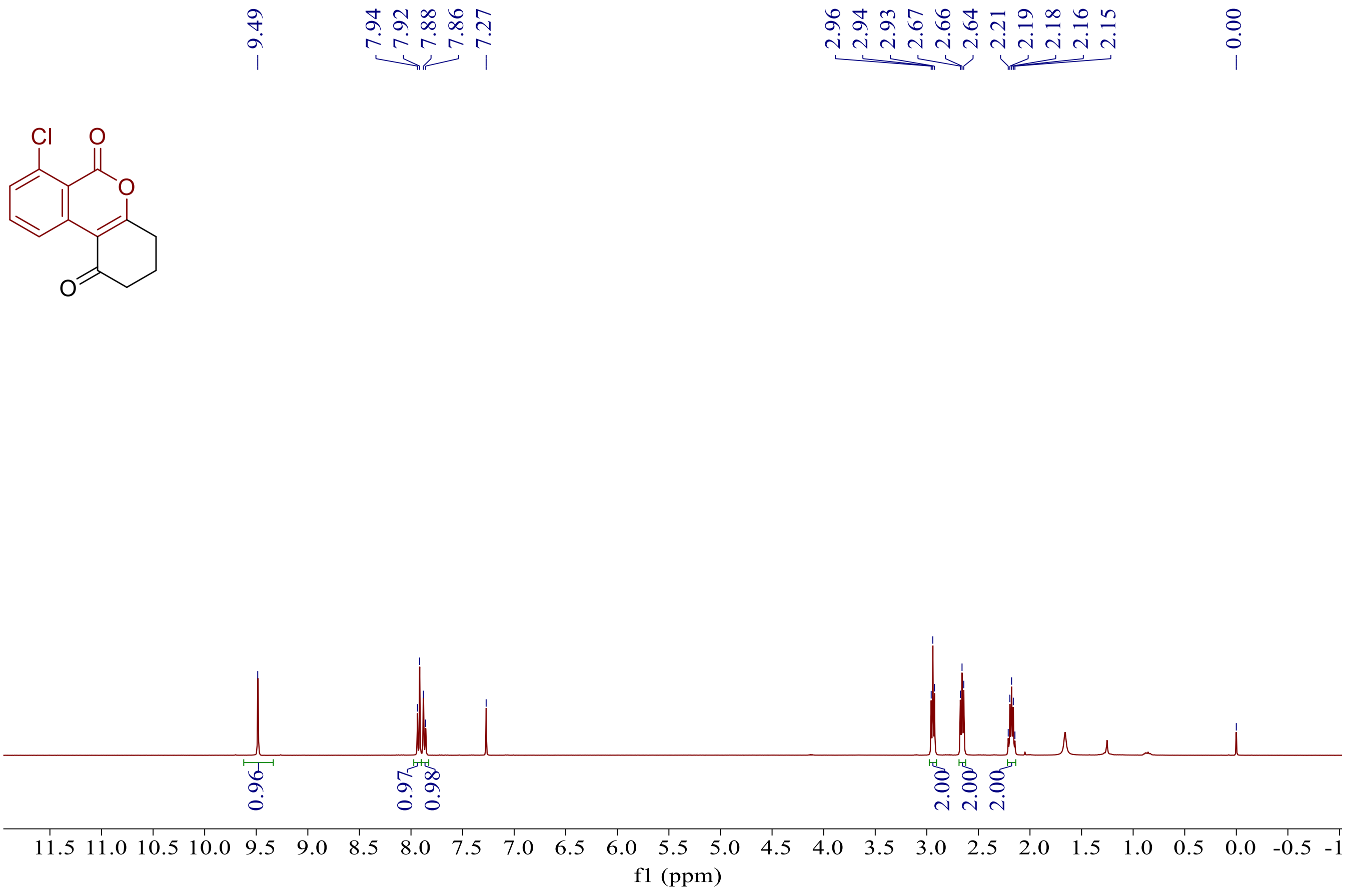

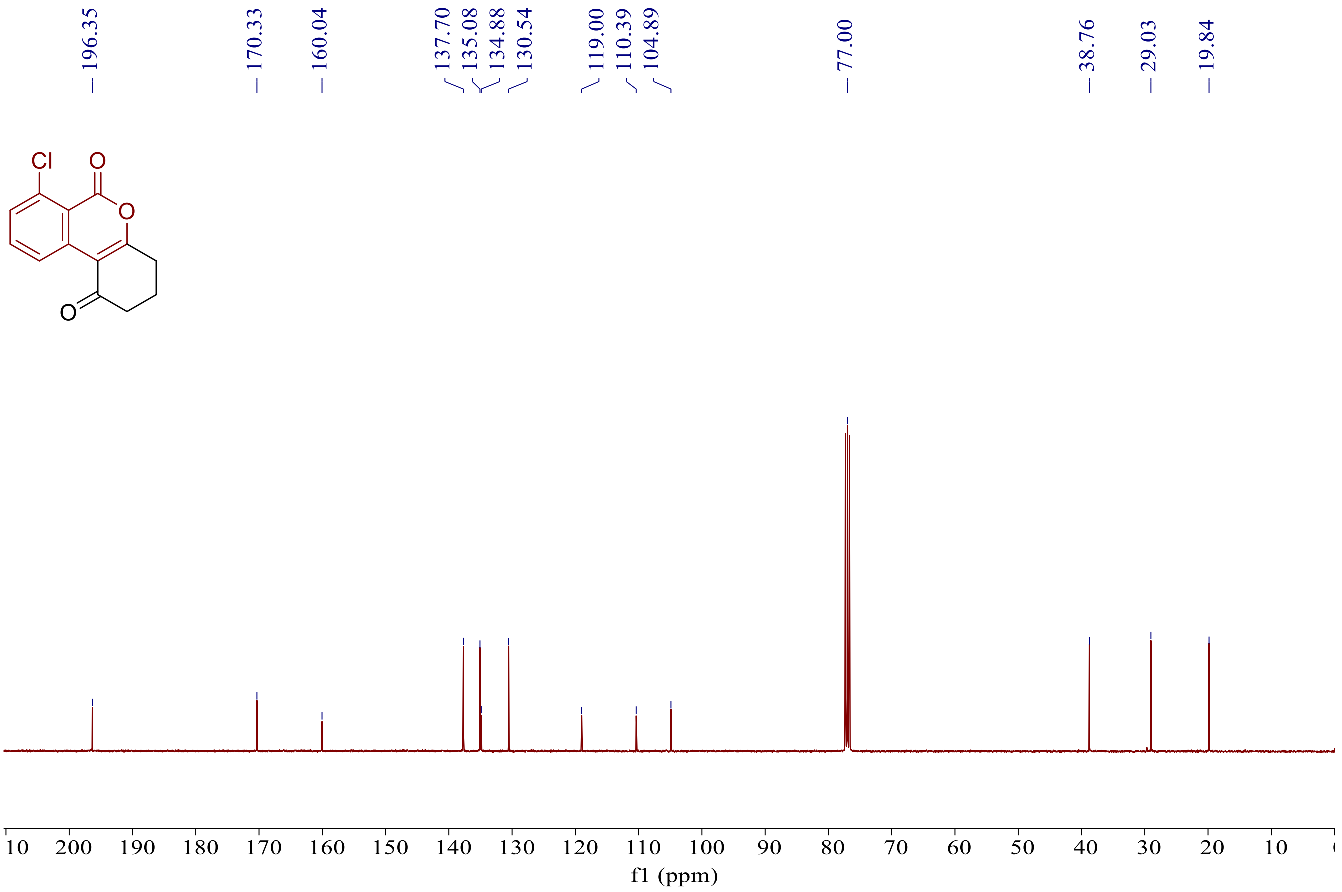

^{1}H NMR (400 MHz, CDCl3) and ^{13}C NMR (100 MHz, CDCl3) spectra of product 3pa

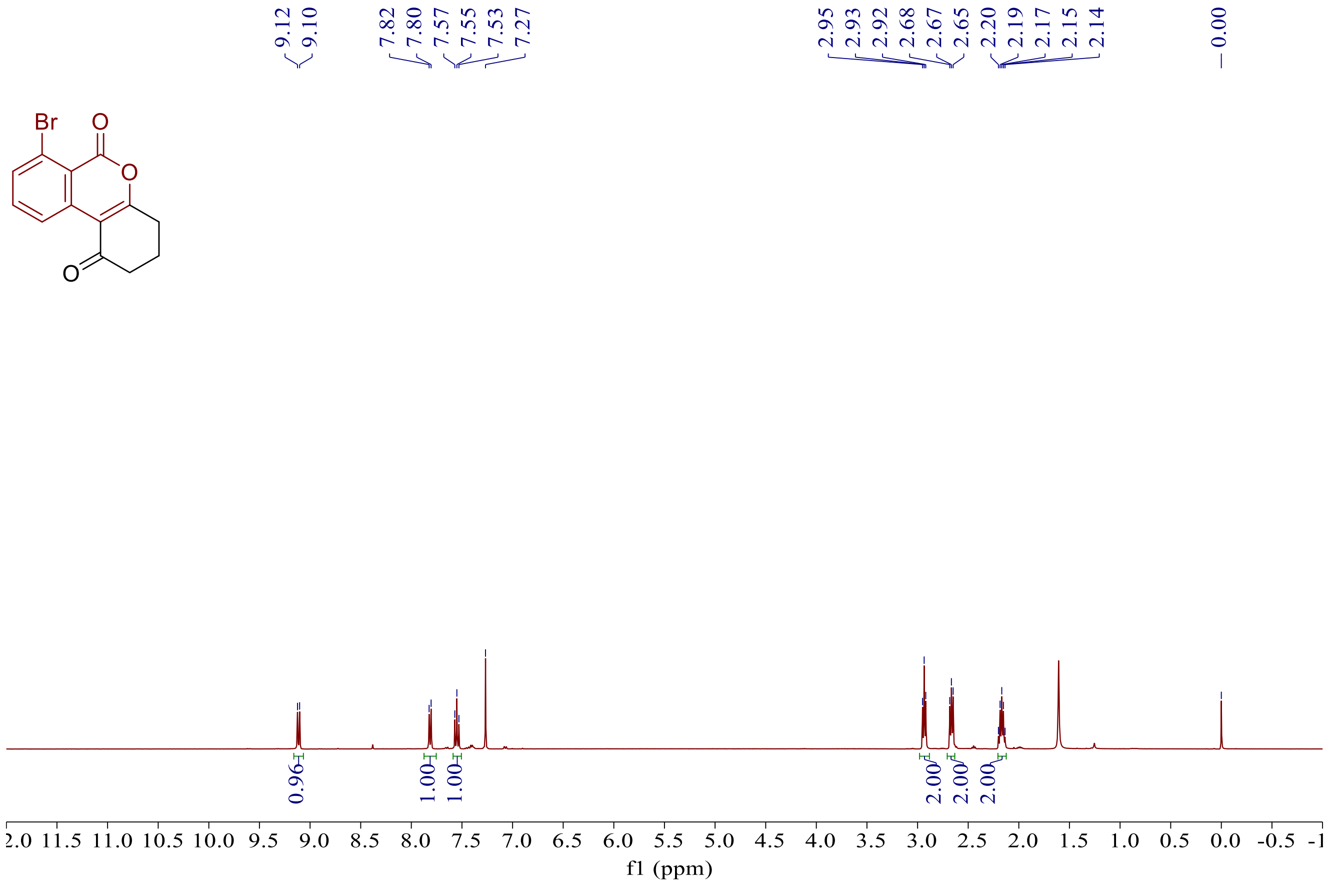

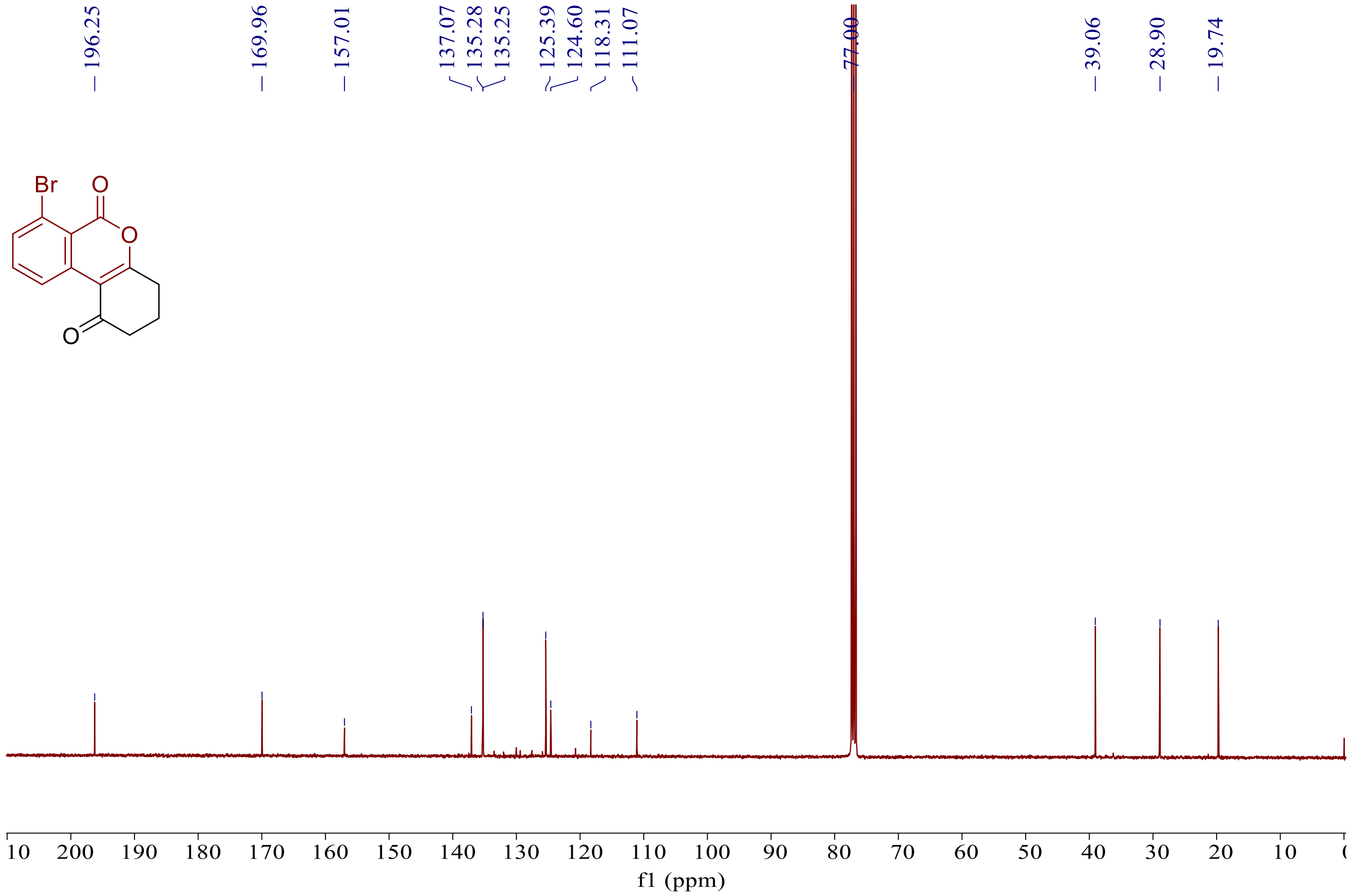

^{1}H NMR (400 MHz, CDCl3) and ^{13}C NMR (100 MHz, CDCl3) spectra of product 3qa

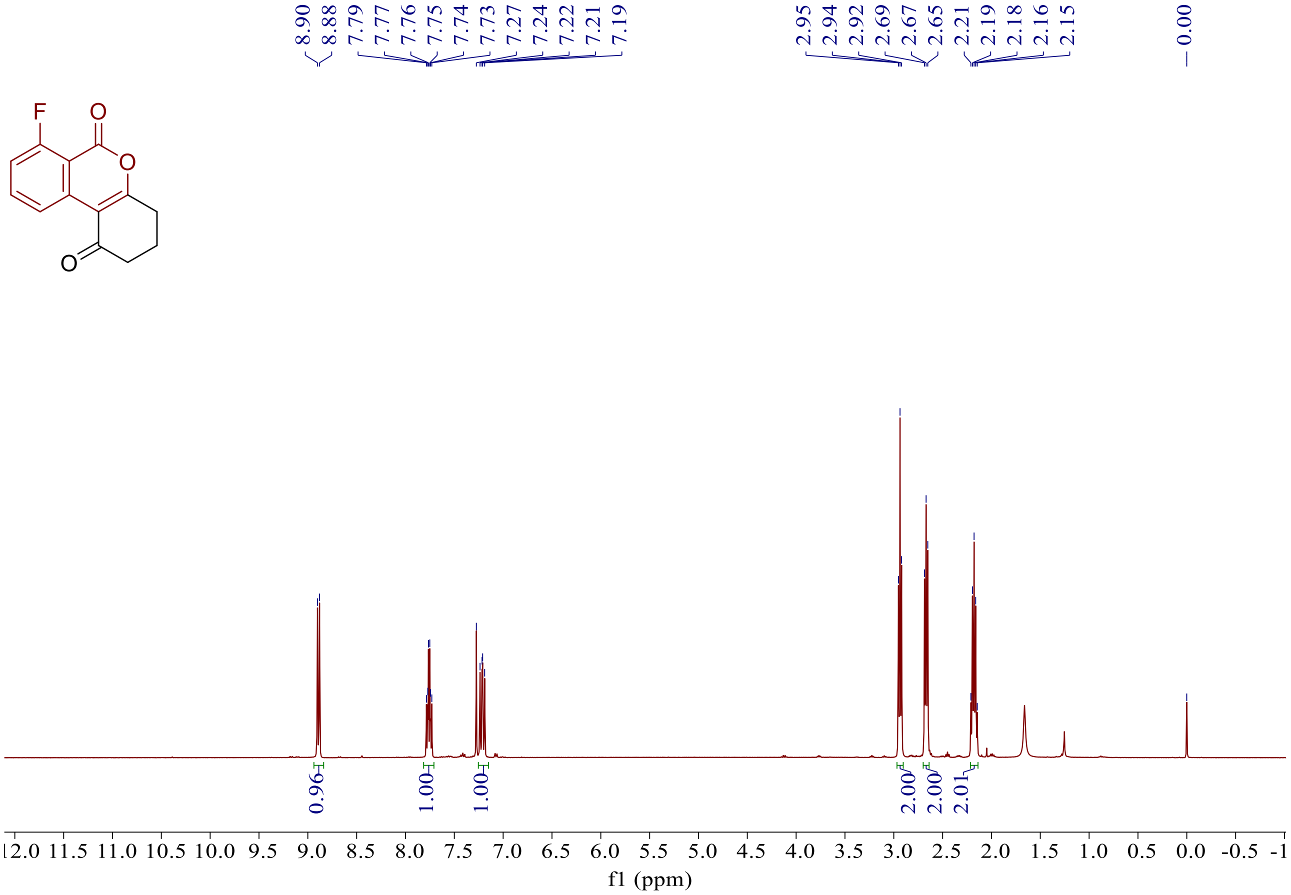

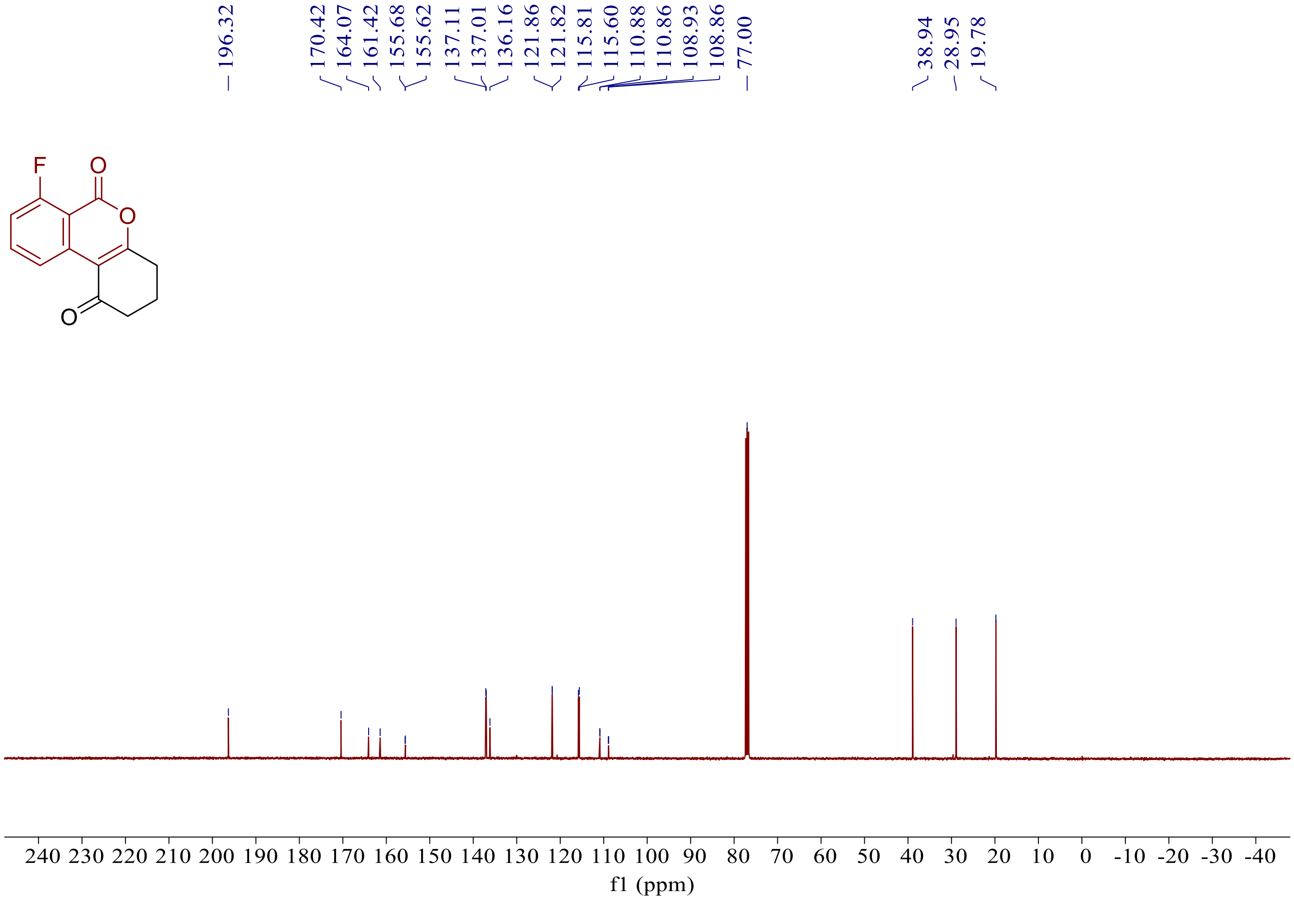

^{1}H NMR (400 MHz, CDCl3) and ^{13}C NMR (100 MHz, CDCl3) spectra of product 3ra

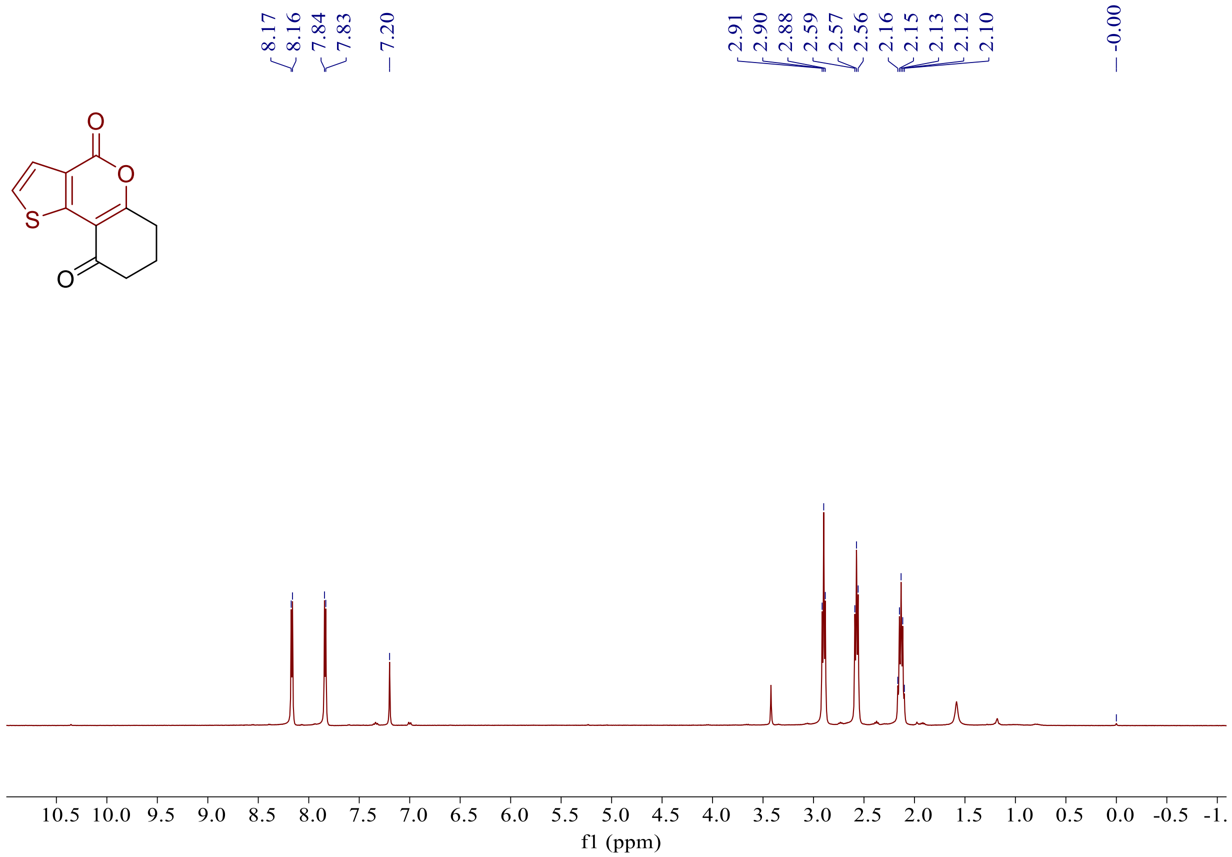

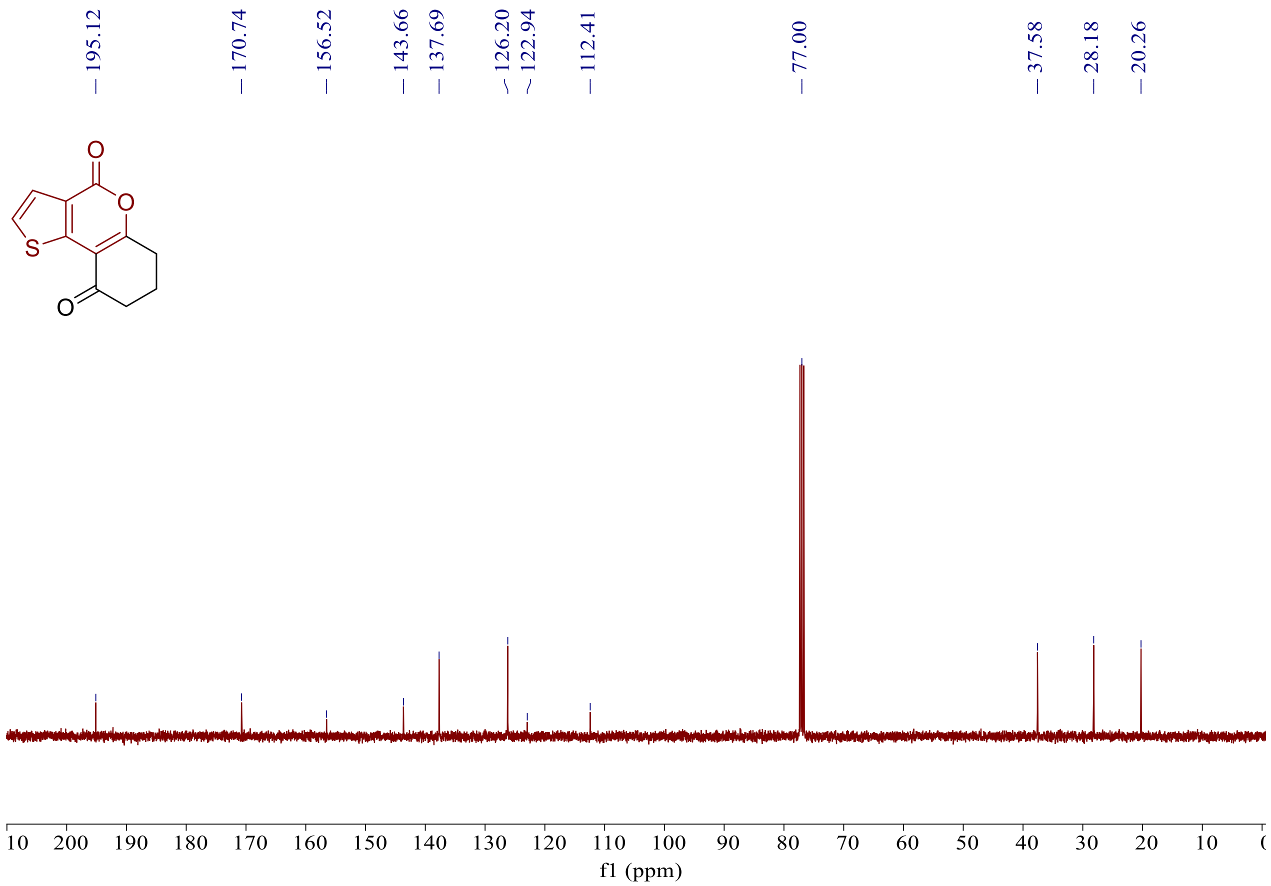

^{1}H NMR (400 MHz, CDCl3) and ^{13}C NMR (100 MHz, CDCl3) spectra of product 3sa

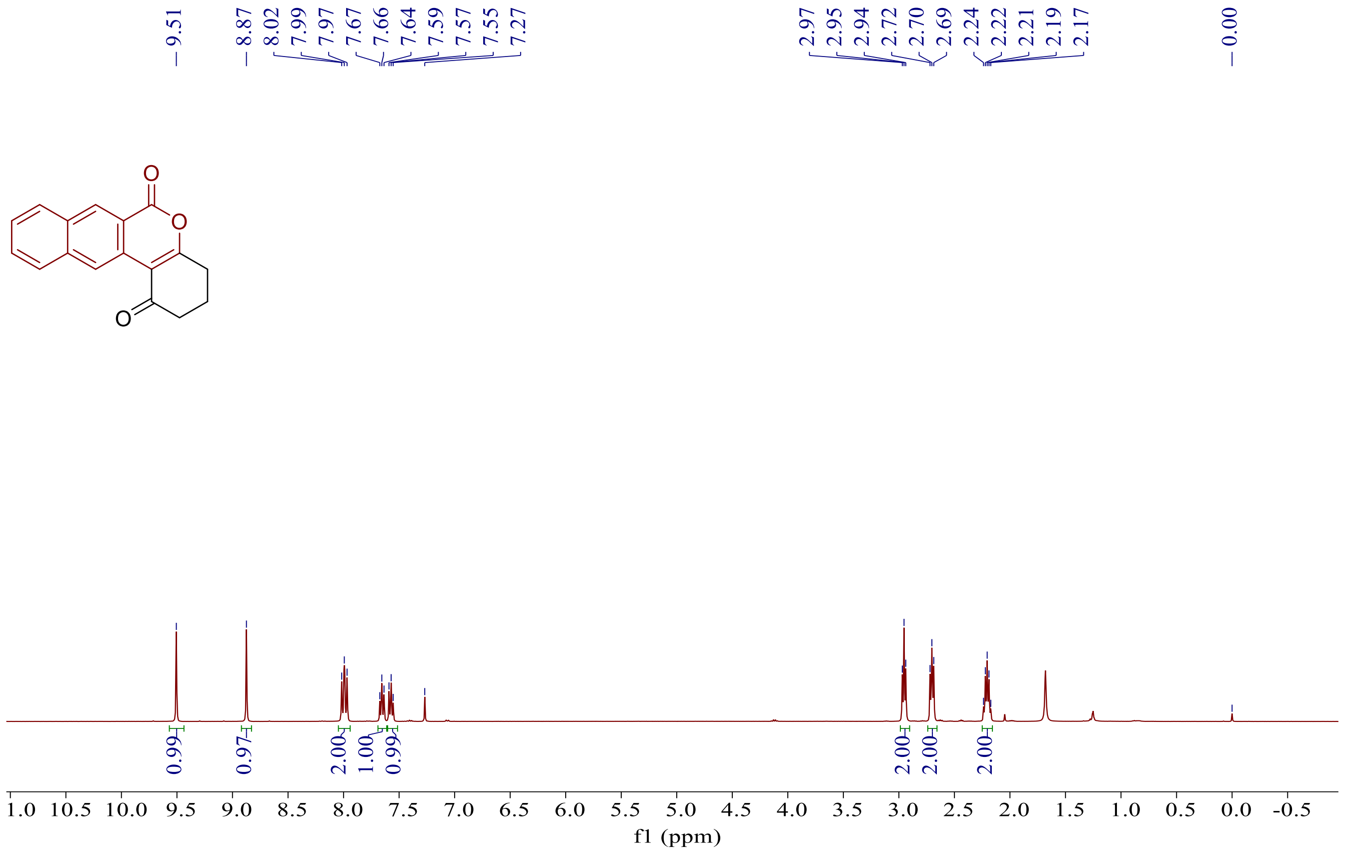

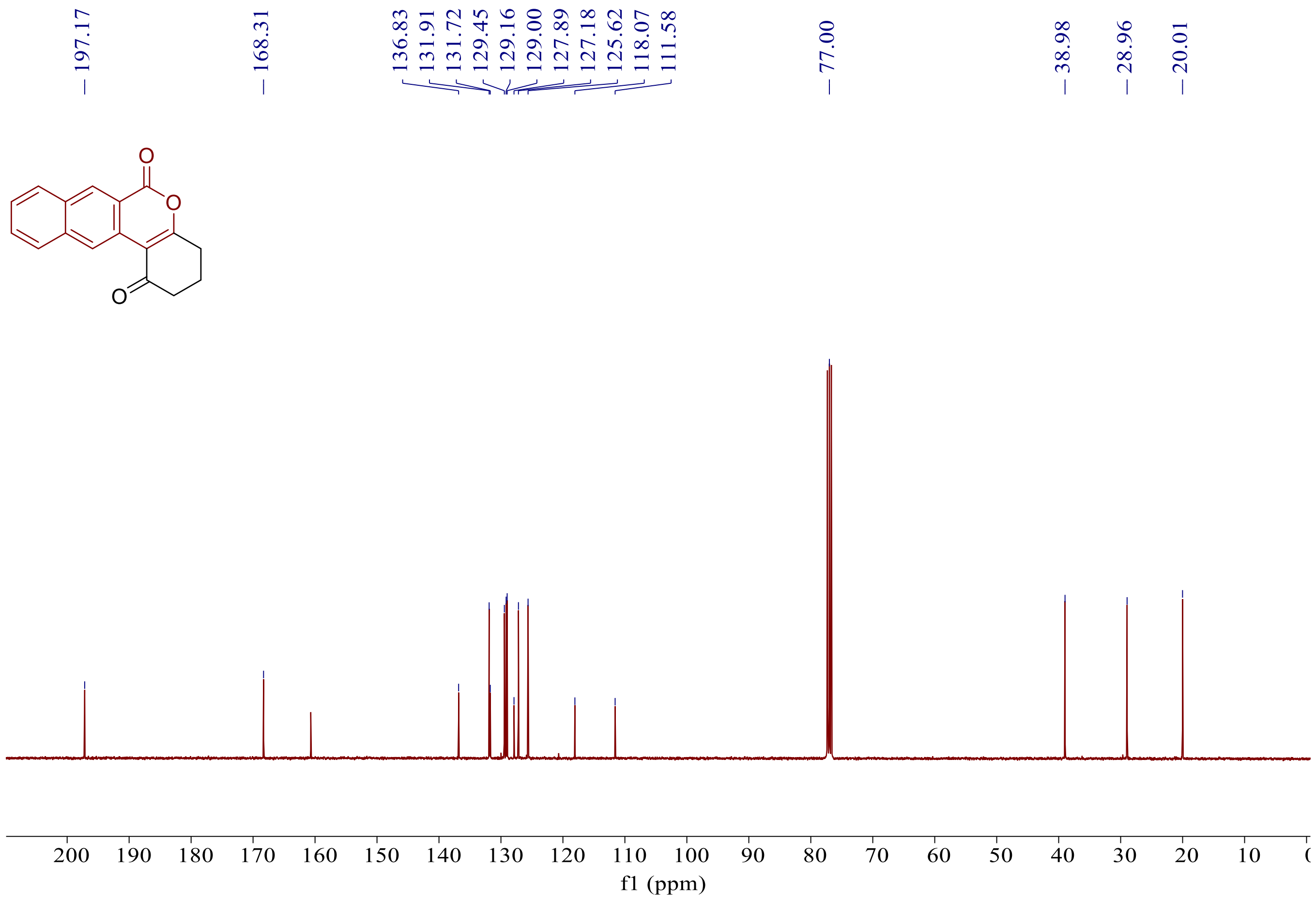

^{1}H NMR (400 MHz, CDCl$_3$) and ^{13}C NMR (100 MHz, CDCl$_3$) spectra of product 3ab

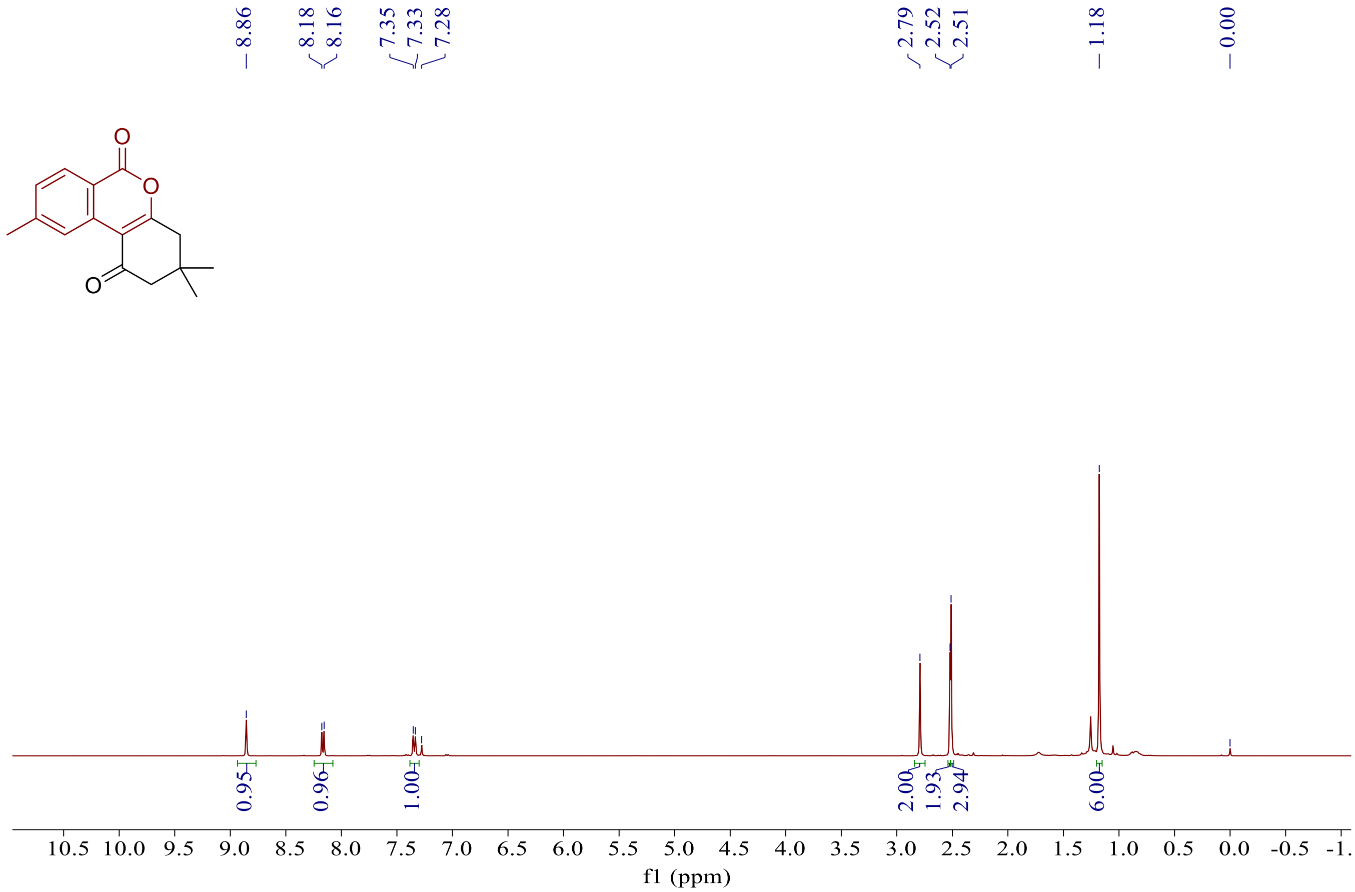

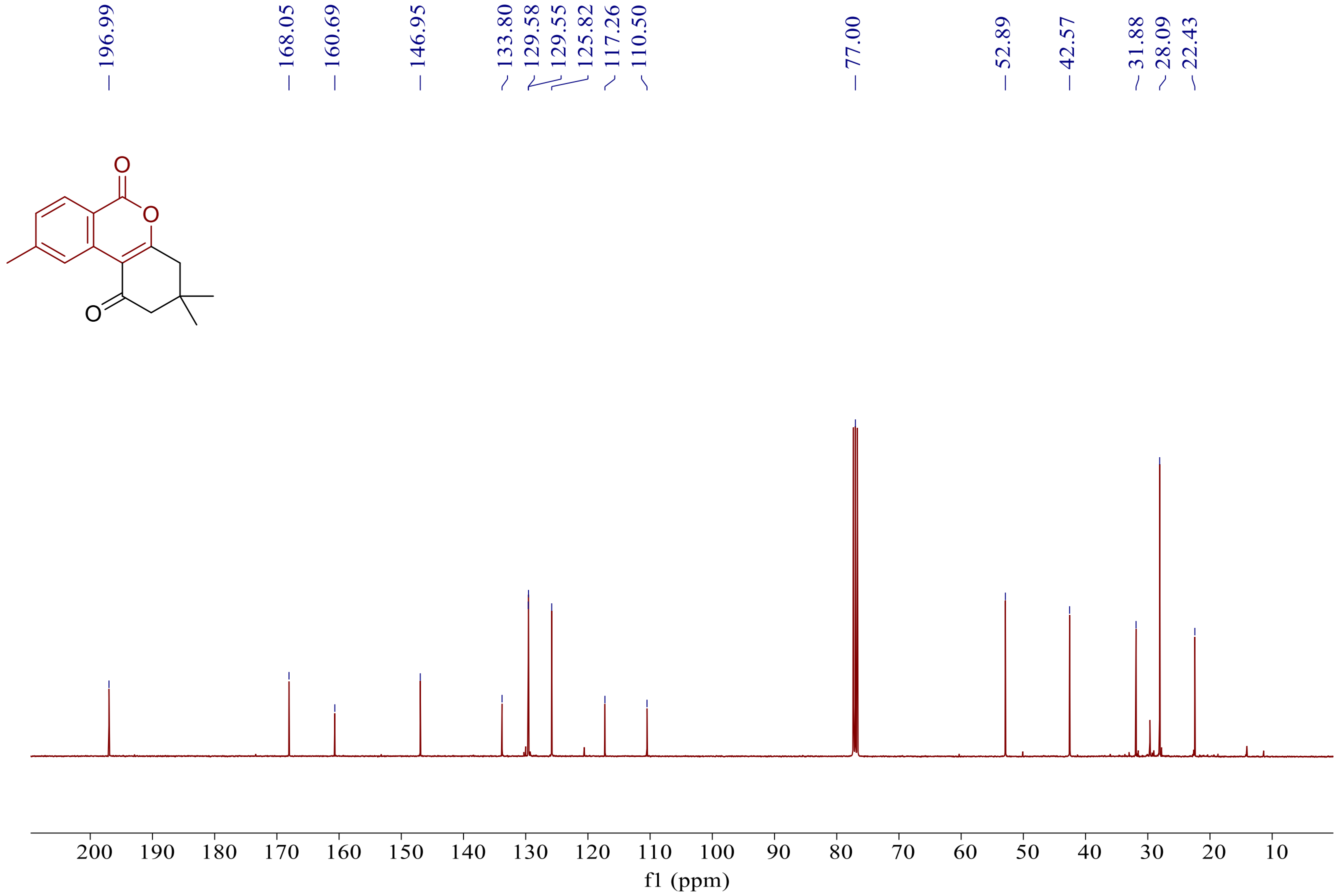

^{1}H NMR (400 MHz, CDCl3) and ^{13}C NMR (100 MHz, CDCl3) spectra of product 3ac

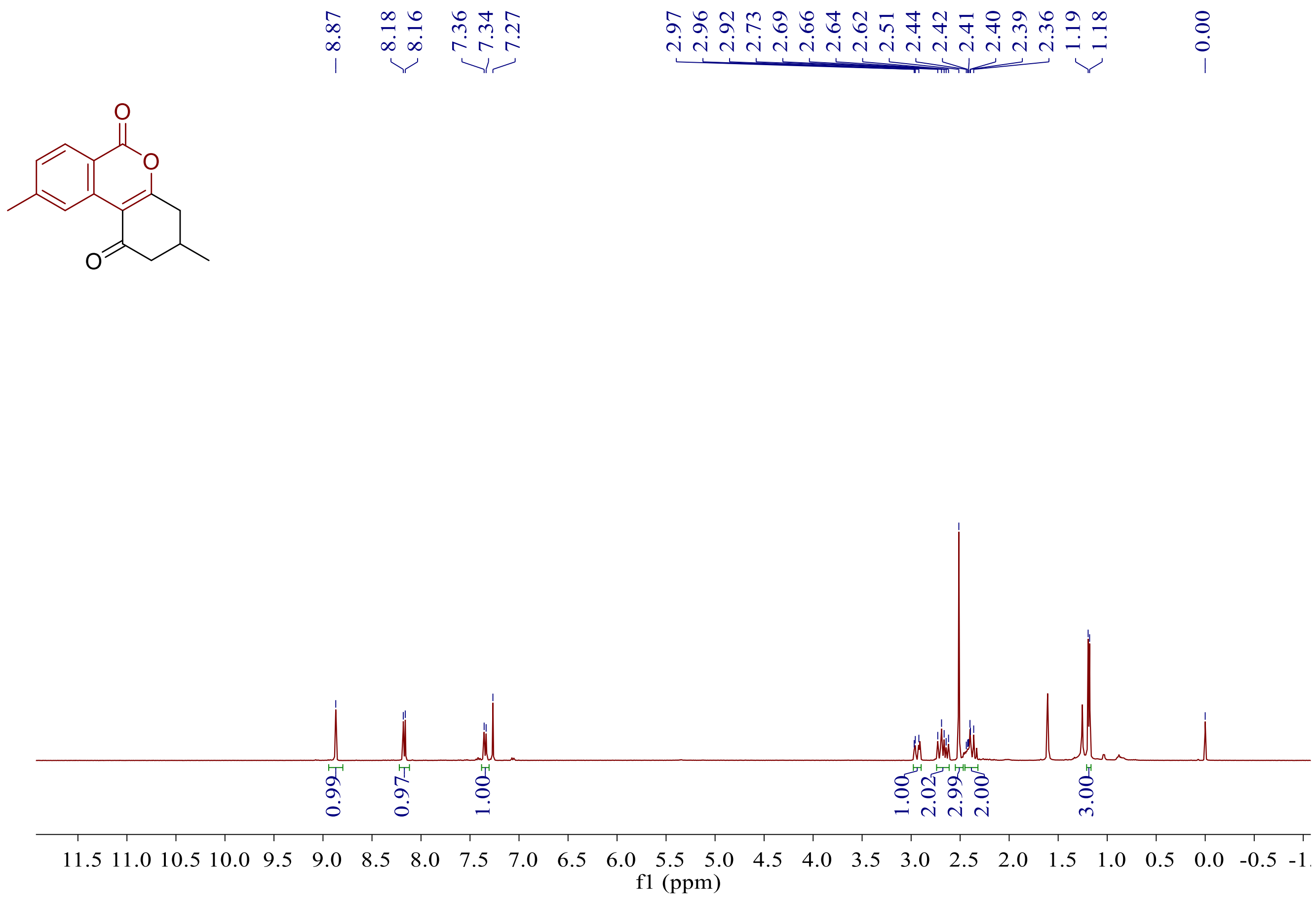

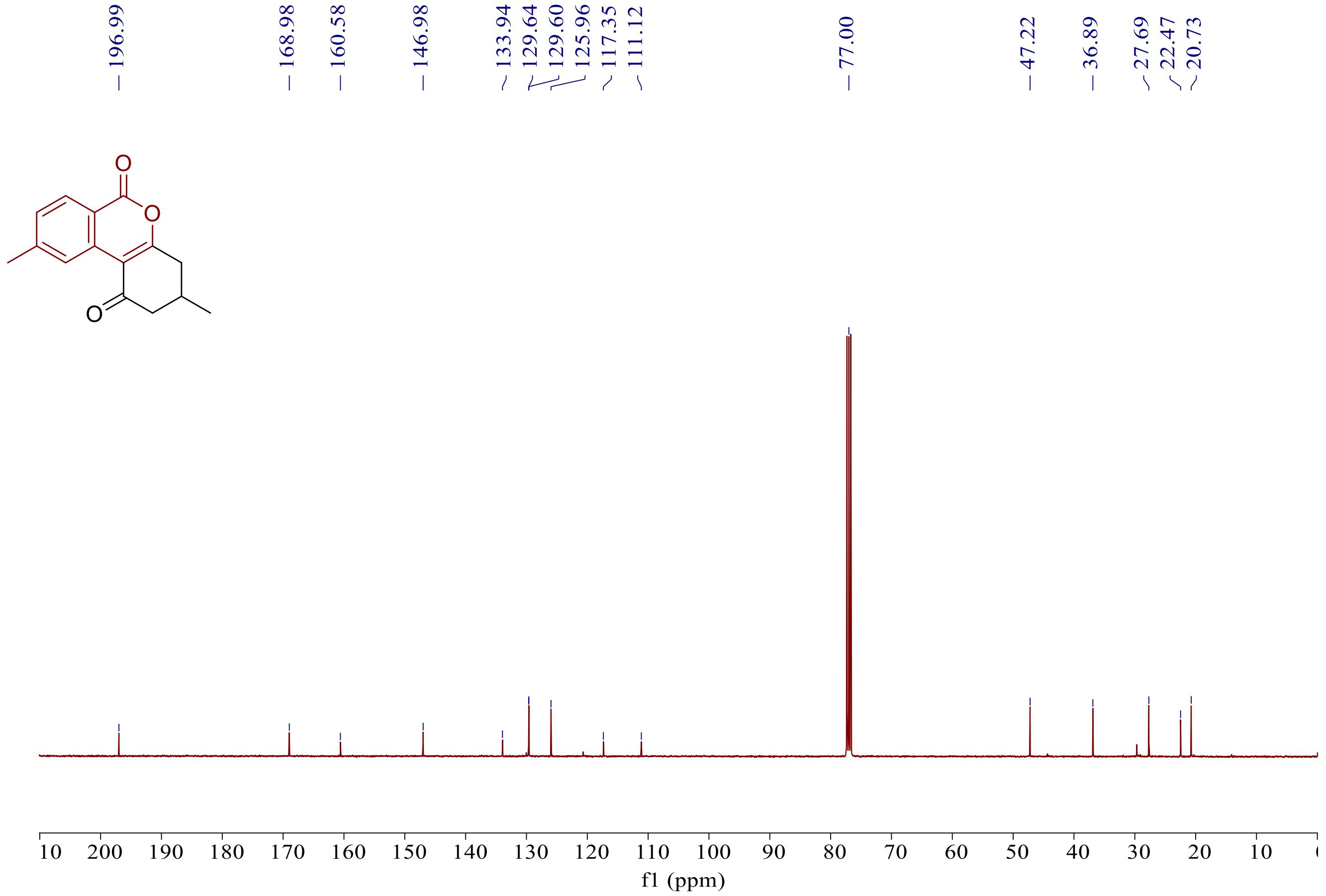

^{1}H NMR (400 MHz, CDCl$_3$) and ^{13}C NMR (100 MHz, CDCl$_3$) spectra of product 3ad

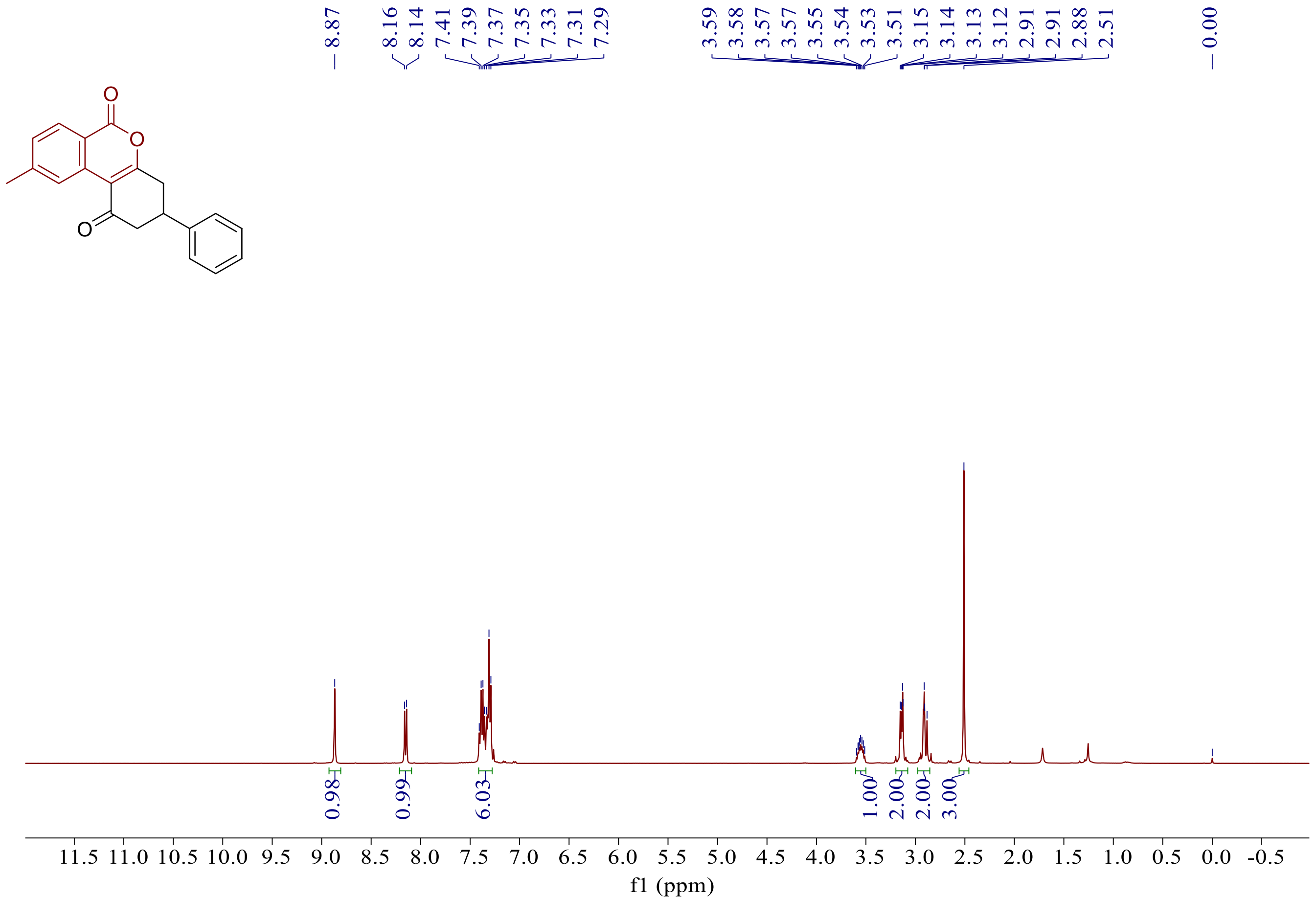

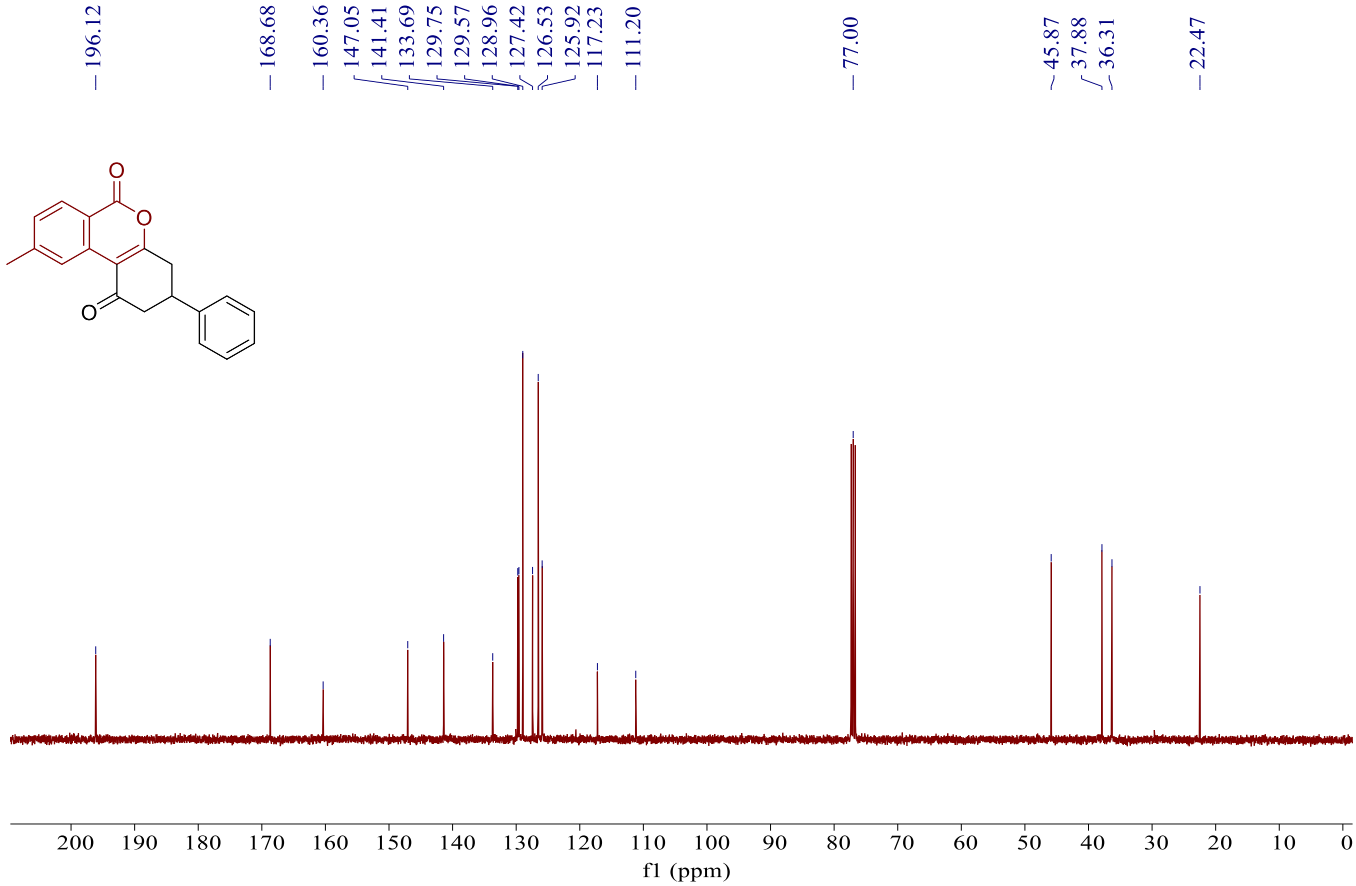

^{1}H NMR (400 MHz, CDCl3) and ^{13}C NMR (100 MHz, CDCl3) spectra of product 3ae

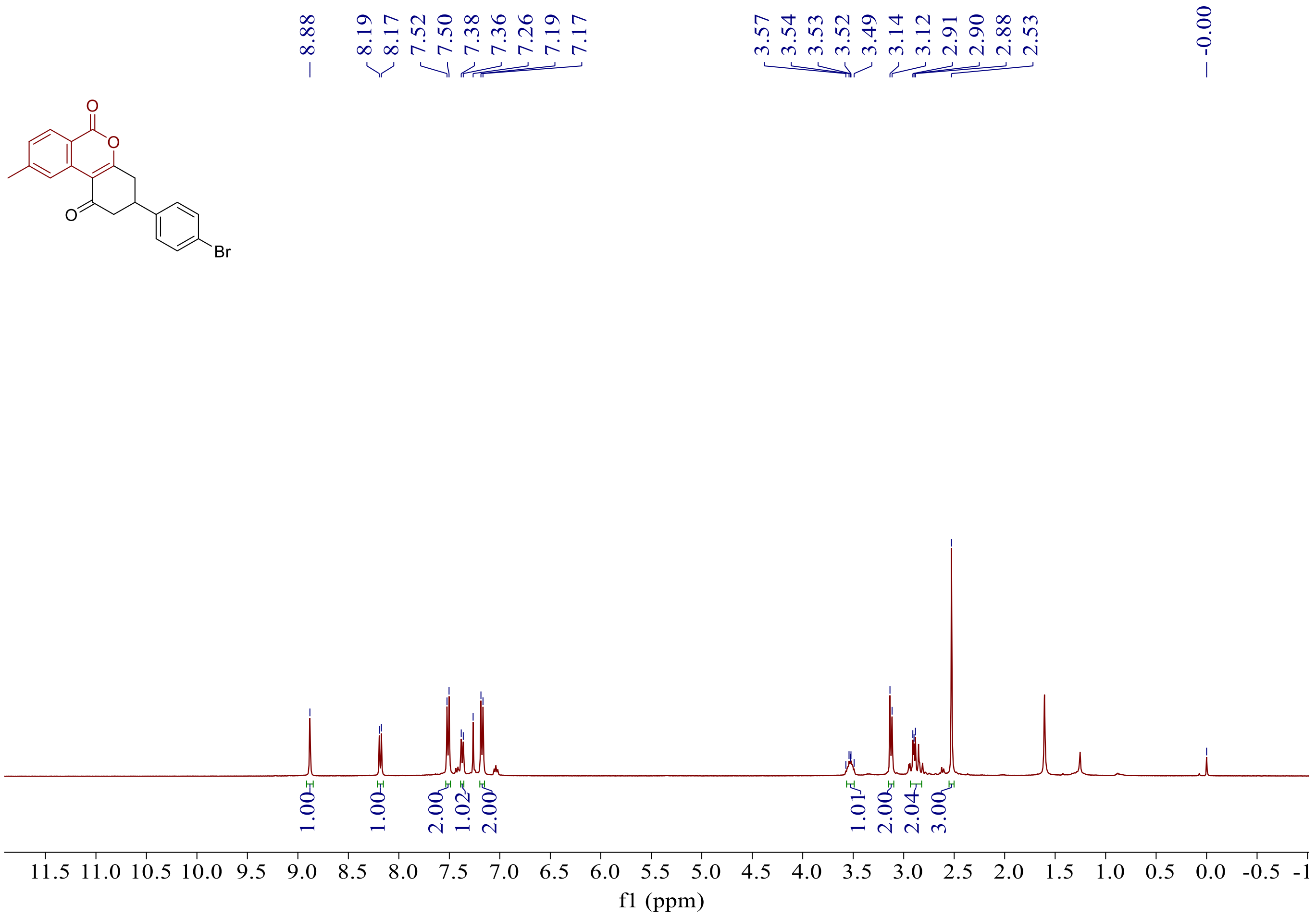

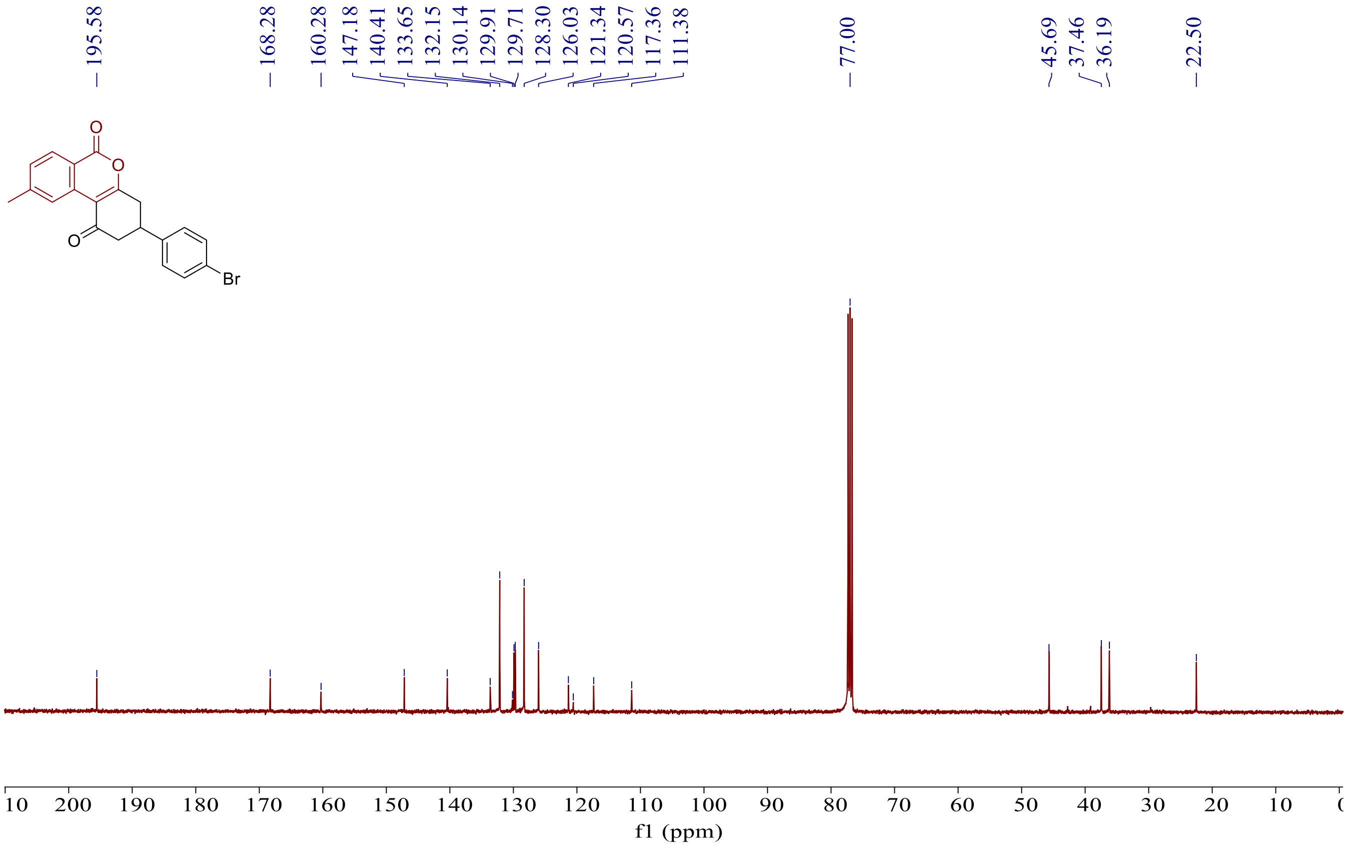

^{1}H NMR (400 MHz, DMSO-*d*6) and ^{13}C NMR (100 MHz, DMSO) spectra of product 4

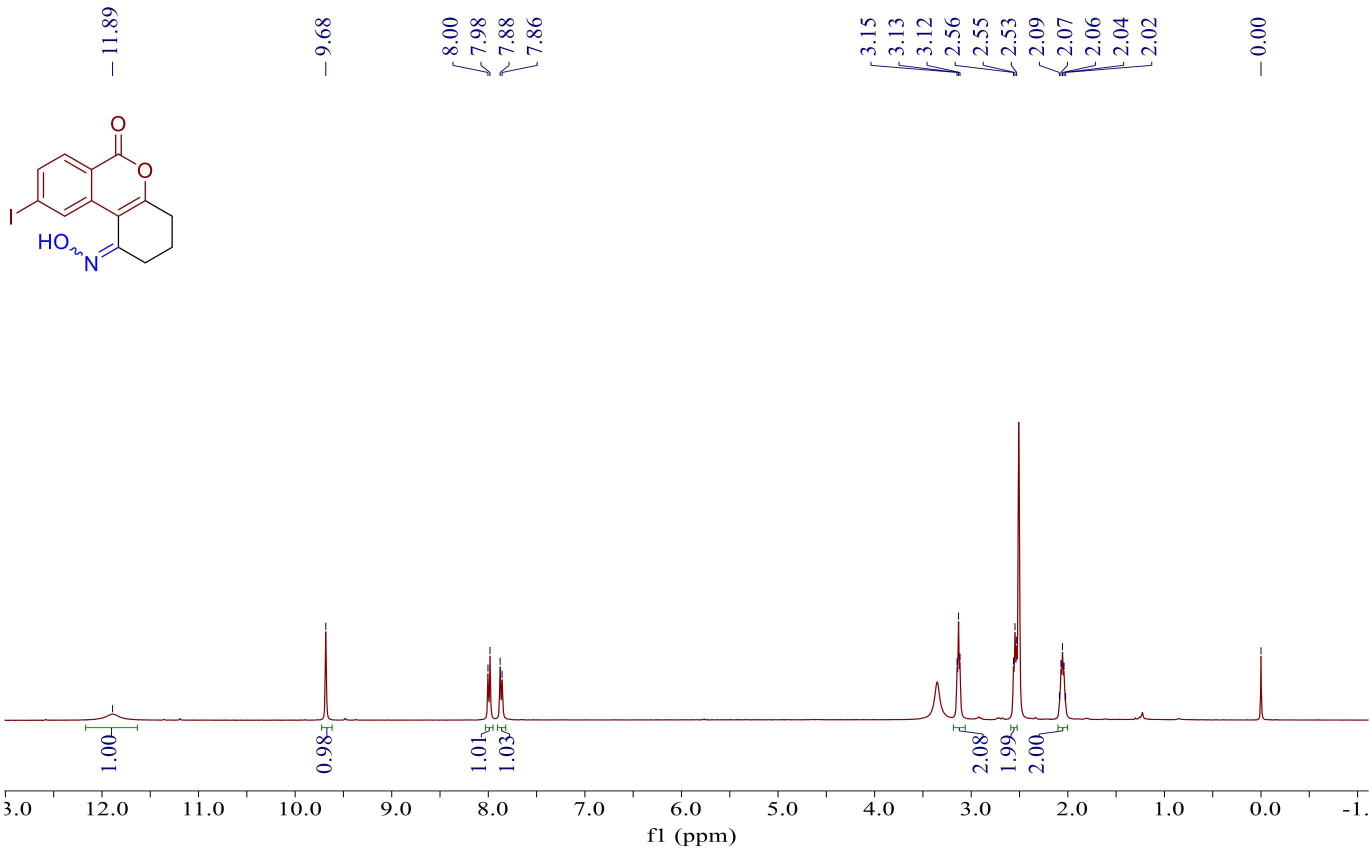

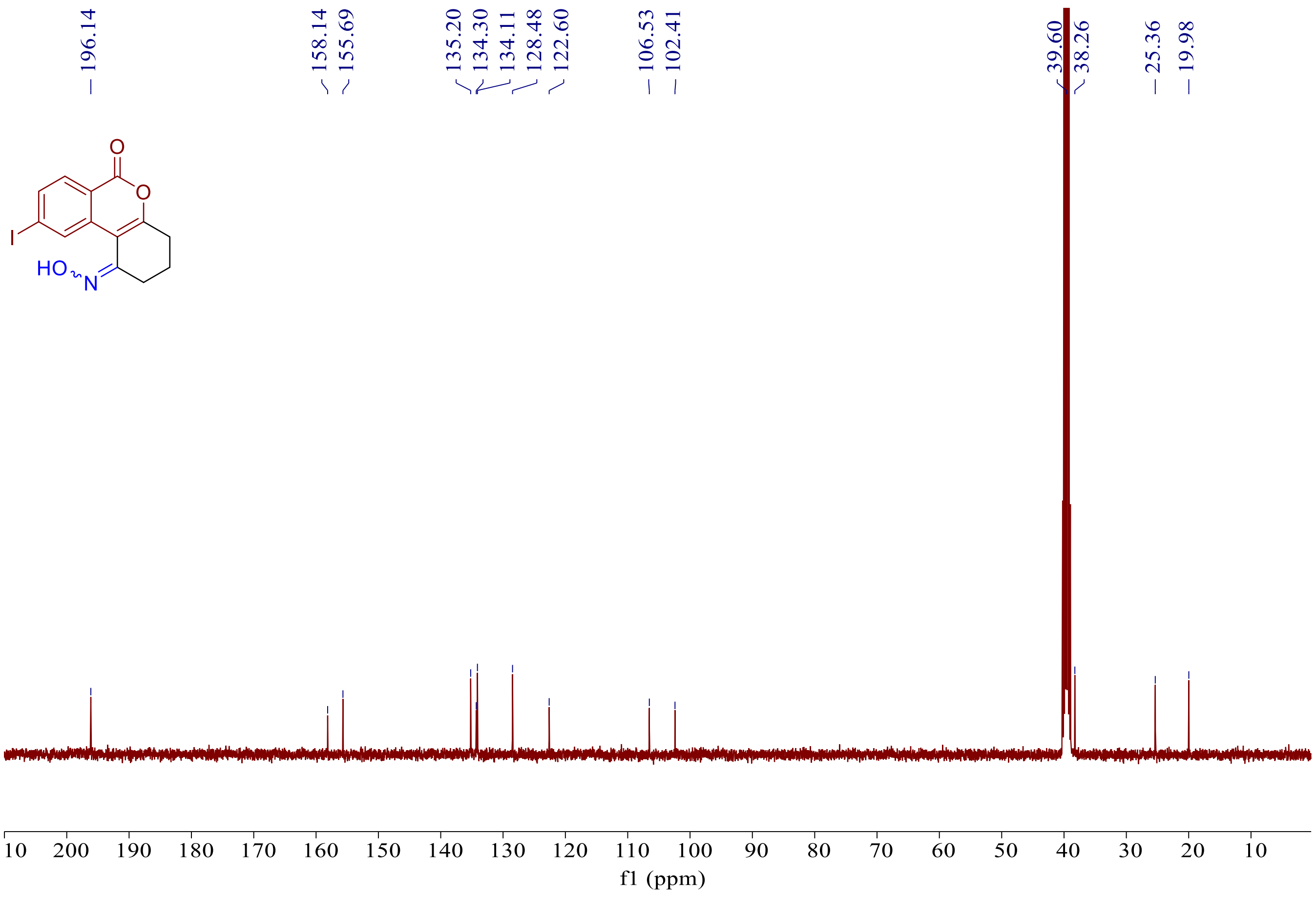

^{1}H NMR (400 MHz, DMSO-d_6) and ^{13}C NMR (100 MHz, DMSO) spectra of product 5

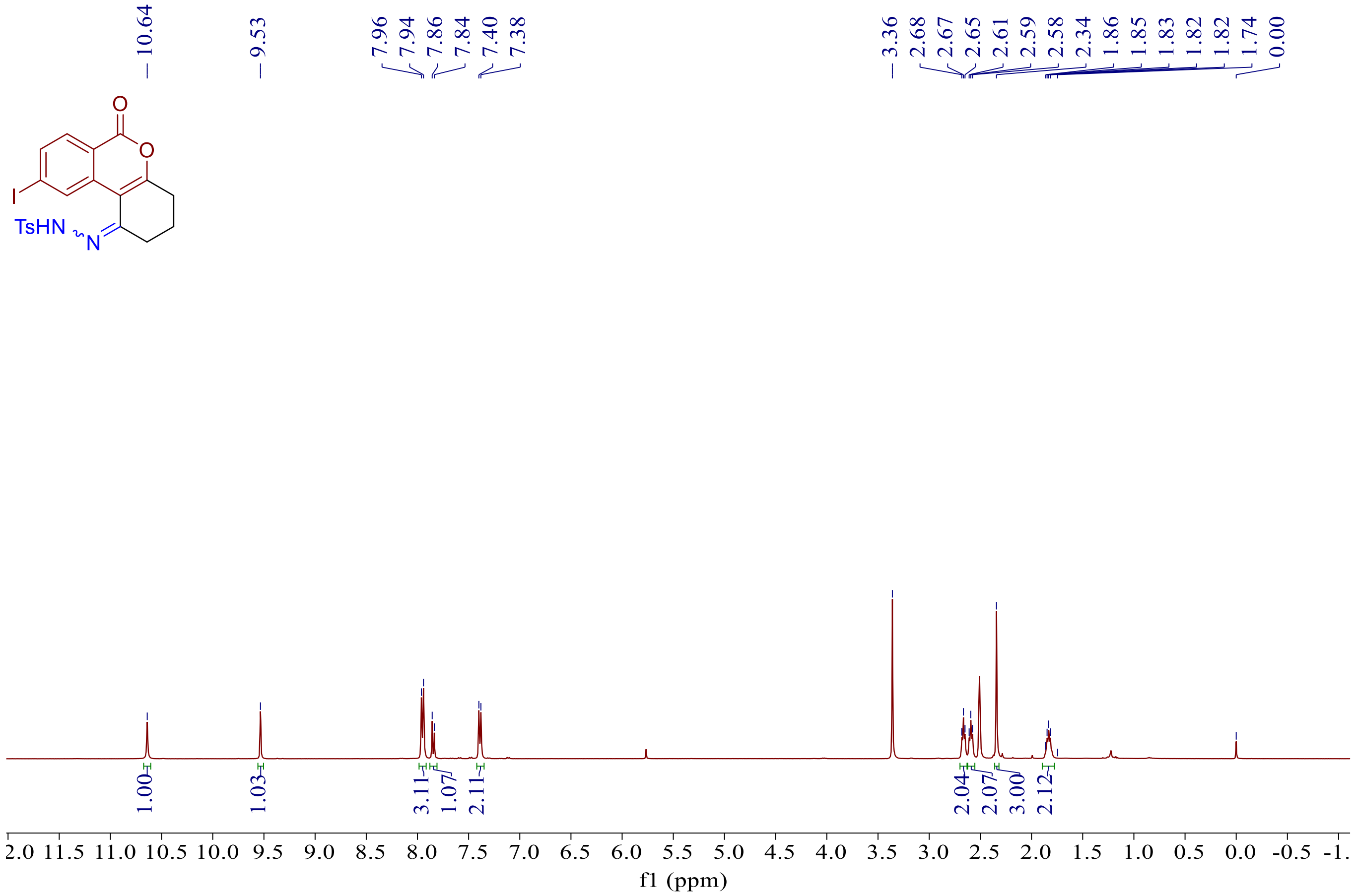

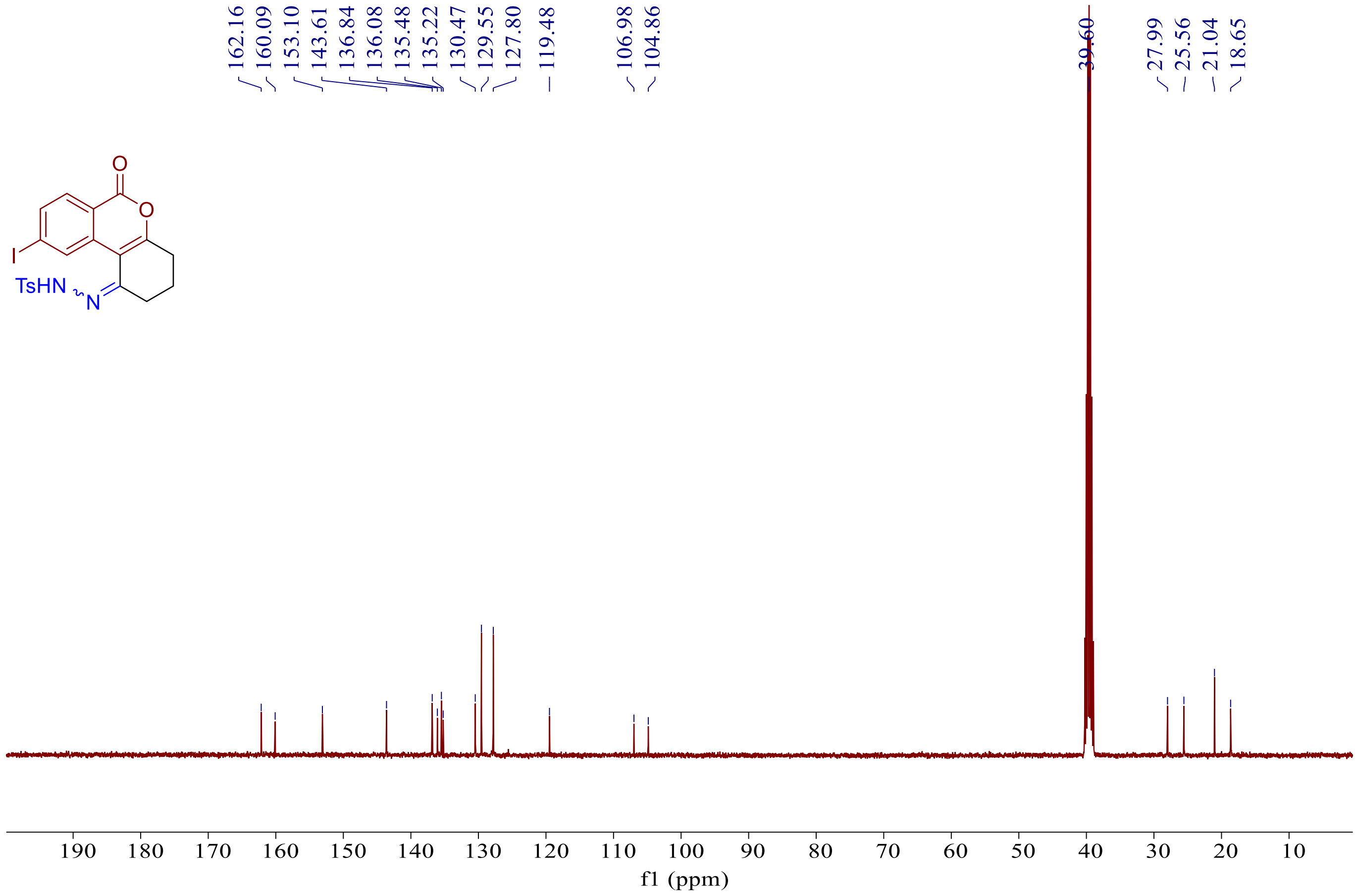

Supplement: File 1 — Experimental and copies of spectra. [file Beilstein_J_Org_Chem-19-100-s001.pdf]
